# Single-Stranded Annealing Induced by Re-Initiation of Replication Origins Provides a Novel and Efficient Mechanism for Generating Copy Number Expansion via Non-Allelic Homologous Recombination

**DOI:** 10.1371/journal.pgen.1003192

**Published:** 2013-01-03

**Authors:** Kenneth J. Finn, Joachim J. Li

**Affiliations:** 1 Department of Biochemistry and Biophysics, University of California San Francisco, San Francisco, California, United States of America; 2 Department of Microbiology and Immunology, University of California San Francisco, San Francisco, California, United States of America; Duke University, United States of America

## Abstract

Copy number expansions such as amplifications and duplications contribute to human phenotypic variation, promote molecular diversification during evolution, and drive the initiation and/or progression of various cancers. The mechanisms underlying these copy number changes are still incompletely understood, however. We recently demonstrated that transient, limited re-replication from a single origin in *Saccharomyces cerevisiae* efficiently induces segmental amplification of the re-replicated region. Structural analyses of such re-replication induced gene amplifications (RRIGA) suggested that RRIGA could provide a new mechanism for generating copy number variation by non-allelic homologous recombination (NAHR). Here we elucidate this new mechanism and provide insight into why it is so efficient. We establish that sequence homology is both necessary and sufficient for repetitive elements to participate in RRIGA and show that their recombination occurs by a single-strand annealing (SSA) mechanism. We also find that re-replication forks are prone to breakage, accounting for the widespread DNA damage associated with deregulation of replication proteins. These breaks appear to stimulate NAHR between re-replicated repeat sequences flanking a re-initiating replication origin. Our results support a RRIGA model where the expansion of a re-replication bubble beyond flanking homologous sequences followed by breakage at both forks in *trans* provides an ideal structural context for SSA–mediated NAHR to form a head-to-tail duplication. Given the remarkable efficiency of RRIGA, we suggest it may be an unappreciated contributor to copy number expansions in both disease and evolution.

## Author Summary

Duplications and amplifications of chromosomal segments are frequently observed in eukaryotic genomes, including both normal and cancerous human genomes. These copy number variations contribute to the phenotypic variation upon which natural selection acts. For example, the amplification of genes whose excessive copy number facilitates uncontrolled cell division is often selected for during tumor development. Copy number variations can often arise when repetitive sequence elements, which are dispersed throughout eukaryotic genomes, undergo a rearrangement called non-allelic homologous recombination. Exactly how these rearrangements occur is poorly understood. Here, using budding yeast to model this class of copy number variation, we uncover a new and highly efficient mechanism by which these variations can be generated. The precipitating event is the aberrant re-initiation of DNA replication at a replication origin. Normally the hundreds to thousands of origins scattered throughout a eukaryotic genome are tightly controlled such that each is permitted to initiate only once per cell cycle. However, disruptions in these controls can allow origins to re-initiate, and we show how the resulting DNA re-replication structure can be readily converted into a tandem duplication via non-allelic homologous recombination. Hence, the re-initiation of DNA replication is a potential source of copy number variation both in disease and during evolution.

## Introduction

Duplication or amplification of chromosomal segments is important for evolution, phenotypic variation, human genetic disorders, and cancer [1]–[5]. Many of these duplications or amplifications are arranged in direct tandem repeat and have homologous sequence elements at their boundary, suggesting they were formed through recombination between non-allelic homologous sequences. Evidence for such non-allelic homologous recombination (NAHR) events is found in the genomes of nearly all species, including humans, where almost half of the human genome is comprised of low or high copy number repeat sequences [6]–[8].

The mechanisms responsible for these duplications or amplifications have been difficult to discern because these events are usually too rare to characterize their molecular intermediates. Nonetheless, studies primarily in microorganisms have led to a number of models for how these duplications/amplifications might arise. The most established model assumes that these NAHR events occur through the same fundamental mechanism as allelic homologous recombination [9]–[11]. In this model NAHR is initiated by a simple DNA double-strand break (DSB) in a repeat sequence, which normally provokes a homology search for the intact allelic counterpart as a repair template. An imperfect search arising from misalignment of sister chromatids or homologs, however, would lead to establishment of a double Holliday junction structure between non-allelic homologous sequences that can resolve into an unequal crossover. Evidence of the reciprocal copy number expansions and contractions expected to arise from such unequal crossing over is limited, having only been observed in the context of large tandem arrays of rDNA [12], [13] or *CUP1* repeats [14], at subtelomeric repeats [15], and in some human genetic disorders [16]. A recent variation on this model suggests that NAHR-mediated tandem duplications/amplifications may be generated by break-induced replication (BIR) [17]. In this model, a broken chromosomal end initiates strand invasion and replication fork assembly at a non-allelic homologous sequence. The fork then duplicates the chromosomal segment between the homologous sequences before proceeding to the end of the chromosome. Again, direct support for this model is minimal.

Recently, we demonstrated that re-replication of a chromosomal segment due to dysregulation of replication controls can efficiently induce NAHR-medicated tandem duplication/amplification of that segment in the budding yeast *Saccharomyces cerevisiae* [18]. Importantly, introduction of simple DSBs failed to induce duplication/amplification with similar efficiency. Our findings raise the possibility that an alternative mechanism initiated by the loss of replication control might be responsible for some NAHR-mediated tandem duplications/amplifications. We have thus been eager to elucidate the mechanism of re-replication induced gene amplification (RRIGA).

The initiation of eukaryotic DNA replication is controlled by a battery of overlapping mechanisms that prevent re-initiation of DNA replication from the hundreds to thousands of replication origins in eukaryotic genomes. Replication initiation normally occurs at these origins in a two-stage process [19], [20]. In G1 phase, origins are licensed for initiation by loading the Mcm2-7 core replicative helicase onto them, a process that requires the origin recognition complex (ORC), Cdc6, Cdt1, and Mcm2-7. During S-phase, licensed origins are triggered to initiate DNA replication. To ensure that none of these origins re-initiate, multiple mechanisms inhibit ORC, Cdc6, Cdt1, and Mcm2-7 to minimize the chance that origins will be relicensed after they have initiated [19]–[21]. Consistent with the non-redundant nature of these controls, experimentally inactivating increasing numbers of these mechanisms leads to progressively increasing amounts of re-initiation and re-replication in budding yeast [22], [23].

These replication controls are critical for cell viability and genome stability. When sufficient controls are disrupted to cause overt re-replication (i.e. an increase in genomic DNA content detectable by flow cytometry), extensive DNA damage and a major DNA damage response is observed [24]–[31]. While the source of damage is not well understood, the amount of damage apparently overwhelms the DNA damage response and leads to massive cell death. In budding yeast, we developed the ability to induce and detect much lower levels of re-replication compatible with cell viability [23]. Retention of viability allowed us to examine the effect of re-replication on genome stability [18]. We found that limited, transient re-replication of a chromosomal segment induced tandem duplication and occasionally higher order amplification of that segment at a rate approximating 10^−2^ per cell division, about five orders of magnitude higher than spontaneous duplication rates [17]. The tandem duplications were bounded by Ty retrotransposon elements, a class of repetitive elements scattered throughout the yeast genome [32].

Here we uncover the mechanism of this re-replication induced gene amplification. These studies show that re-replication induces DNA damage because re-replication forks are highly susceptible to breakage. Our data support a model for RRIGA in which the two forks of a re-replication bubble both proceed beyond repetitive sequence elements flanking the re-initiating origin and then break. Should these breaks occur in *trans* with respect to the chromosome axis, normal 5′ to 3′ strand resection at each break will expose complementary strands of the non-allelic repetitive elements, providing a ready substrate for recombination by a single strand annealing (SSA) mechanism. Such repair of these broken re-replication bubbles will result in a tandem duplication arranged in direct repeat. In this model both the susceptibility of re-replication forks to breakage and the special structural context provided by the re-replication bubble contribute to the extraordinary efficiency of RRIGA. Importantly, the critical event triggering the formation of these tandem direct duplications is re-initiation of DNA replication within the duplicated segment. The remarkably efficient channeling of these re-initiation events into tandem direct duplications raises the possibility that even rare spontaneous re-initiation events may be a potent source of copy number variation in evolution and disease.

## Results

### Homology at Amplicon Boundaries Is Necessary and Sufficient for RRIGA

**Figure 1:**
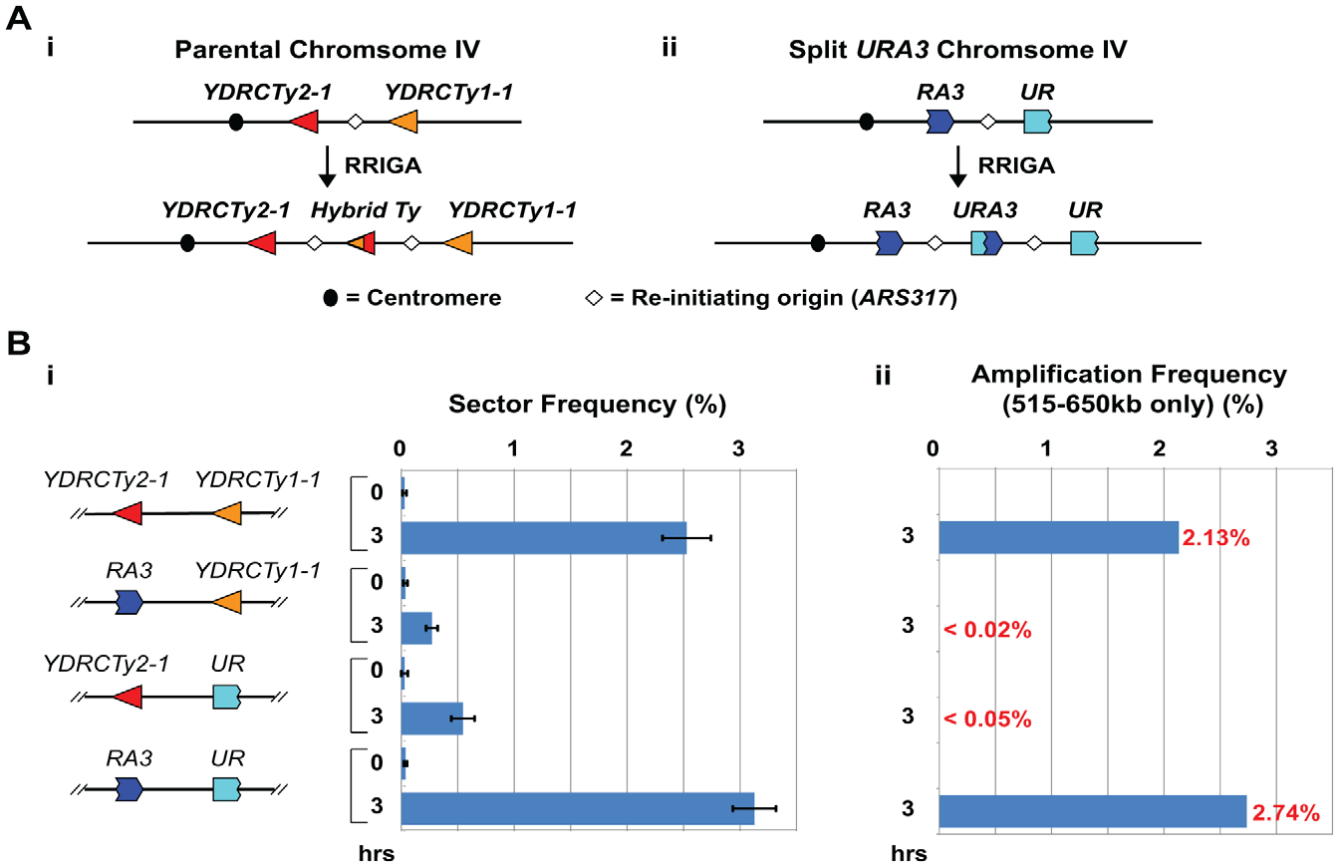
Homologous sequences are necessary and sufficient in *cis* to support RRIGA. A) (i) Schematic of RRIGA arising from NAHR between *YDRCTy2-1* at 515 kb and *YDRCTy1-1* at 650 kb on Chromosome IV; (ii) Schematic of RRIGA arising from NAHR between *3′URA3* (*RA3*) and *5′URA3* (*UR*) fragments replacing *YDRCTy2-1* and *YDRCTy1-1*, respectively (see Figure S1). Frequencies shown are for replacement of Ty and all adjacent LTR and tRNA sequences (version 2). B) (i) Sectoring frequencies (mean ± SEM, n = 3 to 5) before (0 hr) and after (3 hr) induction of re-replication in strains with endogenous Ty elements at 515 kb and 650 kb (YJL8100), with *YDRCTy2-1* replaced by *RA3* (YJL8355), with *YDRCTy1-1* replaced by *UR* (YJL8359), or both Ty elements replaced with the respective *URA3* fragments (YJL8363); (ii) Re-replication induced amplification frequency estimated by multiplying 3 hr sector frequency by fraction of sectors containing 515–650 kb amplification (see Figure S3).

RRIGA generates gene duplications and amplifications arrayed in head-to-tail orientation at the original chromosomal locus with boundaries corresponding to Ty retrotransposable elements [18]. We previously reported that the inter-amplicon junctions generated by RRIGA had hybrid sequences consistent with a non-allelic homologous recombination event between Ty retrotransposable elements that flank the re-initiating origin (Figure 1A(i)). The two Ty elements most frequently involved in our RRIGA experiments share a 1.3 kb region of 99% sequence identity where the recombination events occurred (Figure S1A).

What we did not know was whether the homology between Ty elements is sufficient to promote RRIGA or whether other Ty-associated elements or biology are also important. Most Ty elements, including those involved in our RRIGA studies, are surrounded by tRNA genes and long terminal repeats (LTRs) in inverted orientation. These associated elements are known to cause replication forks to pause and possibly to break [33], [34], disruptions that could stimulate recombination. Hence, if such associated elements are important for RRIGA, it might constrain RRIGA to specific repetitive elements in budding yeast. On the other hand, if homology is sufficient for sequences to serve as RRIGA boundary elements, RRIGA could offer a potential mechanism for a broad range of NAHR-mediated copy number variations.

To address this question we used our previously described RRIGA assay, which exploits colony sectoring [35] to screen for amplification events [18]. In this assay, an origin particularly prone to re-initiate (*ARS317*) when Cdc6, Orc6, and the MCM complex are deregulated is integrated at 567 kb on Chromosome IV, along with a color based copy number reporter gene (*ade3-2p*). Cells with a single copy of *ade3-2p* are pink, while those with two or more copies are red. After transiently inducing re-initiation at *ARS317* during a nocodazole arrest (G2/M), cells are plated for single colonies and possible amplification events are identified from pink colonies with red sectors that comprise 1/2–1/8 of the colony. We then verify and characterize amplifications in the red sectors by array Comparative Genomic Hybridization (aCGH). The vast majority of amplifications identified using this assay span the region from 515–650 kb on Chromosome IV, with *YDRCTy2-1* and *YDRCTy1*-1 at the left and right boundaries, respectively. These are the closest Ty elements flanking the re-initiating *ARS317* origin at 567 kb, and both are surrounded by tRNA genes and LTRs (Figure S1A).

To determine whether homology is sufficient to support RRIGA, we constructed strains in which: (1) *YDRCTy2-1* was replaced by a 3′ portion of the *URA3* gene; (2) *YDRCTy1-1* was replaced by a 5′ portion of the *URA3* gene; or (3) both Ty elements were replaced by their respective *URA3* gene fragments (Figure S1). Two versions of these strains were generated. In version 1 some of the adjacent LTRs were replaced along with each Ty element, but tRNA genes and inverted LTR repeats were preserved (Figure S1B). In version 2, all of the adjacent tRNA genes and LTRs were replaced along with each Ty element (Figure S1C). Importantly, the *URA3* fragments share a 390 bp overlapping region of 100% sequence identity. Thus, sequence homology was present at positions 515 kb and 650 kb on Chromosome IV in the strains in which both endogenous Ty elements were intact, as well as the strains in which both Ty elements were replaced by *URA3* fragments. In contrast, no significant homology was present at these loci in strains in which only one Ty element was replaced by a *URA3* fragment, and we refer to these as non-homologous boundary strains.

The non-homologous boundary strains showed a 5- to 10-fold decrease in sector frequency (Figure 1B(i), Figure S2A, Table S1). Subsequent aCGH analysis of a dozen residual sectors induced in version 2 of each of these non-homologous boundary strains failed to detect any amplifications with endpoints at 515 kb and 650 kb on Chromosome IV (Figure S3, Table S2). Thus, when RRIGA frequencies for the 515–650 kb segment were estimated by multiplying sector frequencies by the percent of sectors that amplified this segment, there was at least a 50- to 100-fold reduction in frequency (Figure 1B(ii)). We note that many red sectors derived from the strain without the right hand Ty element at 650 kb (*YDRCTy1-1*) did have an extra copy of the *ade3-2p* reporter, but achieved this either by using Ty elements further to the right as the right hand RRIGA boundary element, or through translocation or aneuploidy. In contrast, most red sectors derived from the strain without the left hand Ty element at 515 kb (*YDRCTy2-1*) did not have an extra copy of the *ade3-2p* reporter, presumably because there are no other Ty elements on Chromosome IV to serve as left-hand RRIGA boundaries (these red sectors presumably arose from other genomic changes that altered the rate of red pigment accumulation in *ade3-2p* cells). These findings confirmed that homology at the boundaries of amplicons is necessary for efficient RRIGA in budding yeast.

More importantly, when sequence homology was restored by replacing the remaining Ty element with the appropriate *URA3* fragment, RRIGA frequencies were also restored. In strains with Ty elements at both 515 kb and 650 kb replaced by either version 1 or version 2 of the overlapping *URA3* fragments, sectoring occurred at a frequency comparable to the strain with endogenous Ty elements intact (Figure 1B(i), Figure S2A, Table S1). Furthermore, most (14/16) of the red-sectors that were examined by aCGH bore an amplification of the 515 kb to 650 kb region of Chromosome IV (Figure S3, Table S2). Importantly, RRIGA frequency in the context of overlapping *URA3* fragments was unaffected by the presence or absence of the tRNA genes or LTRs (compare Figure 1B and Figure S2A, Figure S2B). Thus, sequence homology at the boundaries of amplicons is sufficient to support RRIGA. Given the prevalence of homologous repetitive elements in eukaryotic genomes [36], this finding implies that these genomes are a potentially rich source of substrates for re-replication induced gene amplification.

### Development of a Selection Assay for RRIGA

**Figure 2:**
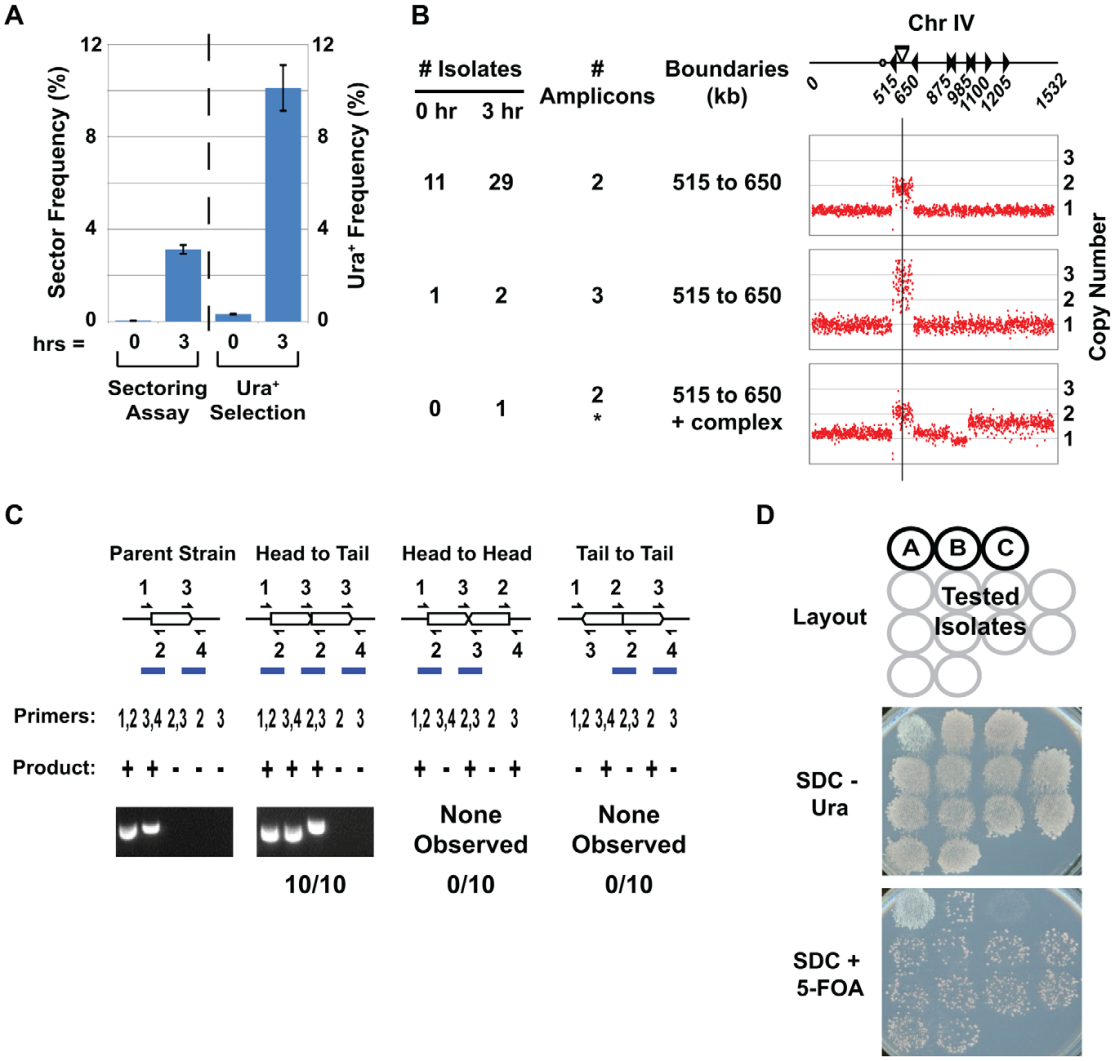
A selection-based assay for detecting RRIGA events. A) Comparison of RRIGA frequencies (mean ± SEM) measured using sectoring assay (n = 4) or *URA3* selection assay (n = 5) in strains (YJL8363/8364) with *YDRCTy2-1* and *YDRCTy1-1* replaced by *URA3* fragments as described for Figure 1B. B) Isolates from *URA3* selection assay have amplifications spanning the segment between *URA3* fragments (Chromosome IV 515–650 kb). aCGH copy number analysis of Chromosome IV shown for 12 isolates selected before (0 hr) and 32 isolates selected after (3 hr) re-replication from YJL8112/8113 and YJL8363/8364. Chromosome IV schematic shows position and orientation of Ty elements (triangles), centromere (circle), and *ARS317-ade3-2p* re-initiation cassette (bar and vertical line). C) Isolates from *URA3* selection assay have amplifications tandemly arrayed *in loco* in direct repeat. The unamplified parental amplicon and three possible orientations for tandem duplications *in loco* are shown schematically. Predicted PCR junction fragments are shown for five sets of primers that flank amplicon boundaries (+, PCR product expected; −, no PCR product expected). Representative PCR products are shown for parental strain YJL8363 and 10 re-replication-induced isolates from B. D) Amplicons appear to be chromosomally integrated but excisable. The 10 isolates tested by PCR in C were grown on non-selective media (YEPD), then replica plated to media lacking uracil (SDC-Ura) or media containing 5-FOA (SDC+5-FOA). Patch A, YJL8698 with extrachromosomal copy of *URA3*. Patch B, YJL6974 with integrated but excisable copy of *URA3*. Patch C, YJL8344 with integrated and un-excisable *URA3*.

The fact that RRIGA in budding yeast results in NAHR between homologous sequences flanking a re-initiating origin allowed us to develop a more rapid and sensitive selection-based assay for quantifying RRIGA. We designed the *URA3* fragments replacing the Ty elements at 515 kb and 650 kb such that NAHR between the fragments during RRIGA reconstitutes a full length, functional *URA3* gene at the inter-amplicon junction (Figure 1A(ii)). Thus, in addition to scoring RRIGA between these two endpoints by colony sectoring, we could select for these events on media lacking uracil (Figure 2A, Table S1, Table S3). We note that the selection assay consistently gave a higher frequency than the sectoring assay, most likely because our visual criterion restricted the sectoring assay to capturing amplification events that occurred within 2–3 generations of cell plating (see Text S1).

We characterized the genetic alterations in the *URA3* prototrophs recovered from our selection assay to ensure that they structurally resembled the RRIGA amplifications previously recovered from the sectoring assay. aCGH demonstrated that all prototrophs did indeed bear an amplification that spans the region from 515–650 kb on Chromosome IV (Figure 2B, Table S4). PCR across potential amplicon junctions confirmed that the original amplicon boundaries were intact and that the regenerated *URA3* gene was created from a new head-to-tail amplicon junction (Figure 2C). Such a junction could arise from tandem intrachromosomal amplicons in head-to-tail orientation, as previously observed for RRIGA, but could also arise from circularization of an extrachromosomal amplicon via NAHR between the two *URA3* fragments. These two possibilities can be distinguished by the spontaneous loss rate of the regenerated *URA3* gene, because the latter will be lost at a much higher rate than the former. This loss rate can be estimated by the frequency of cells lacking *URA3* (and thus resistant to the drug 5-fluoroorotic acid (5-FOA)) that accumulate in a population when selection for the gene is removed. As shown in Figure 2D, all the *URA3* prototrophs obtained from our selection assay accumulated 5-FOA resistance at a frequency expected for an intrachromosomal amplification. Thus, the amplifications detected using the *URA3* selection assay were structurally identical to those observed using the sectoring screen.

### RRIGA Occurs through a Single-Strand Annealing Mechanism

**Figure 3:**
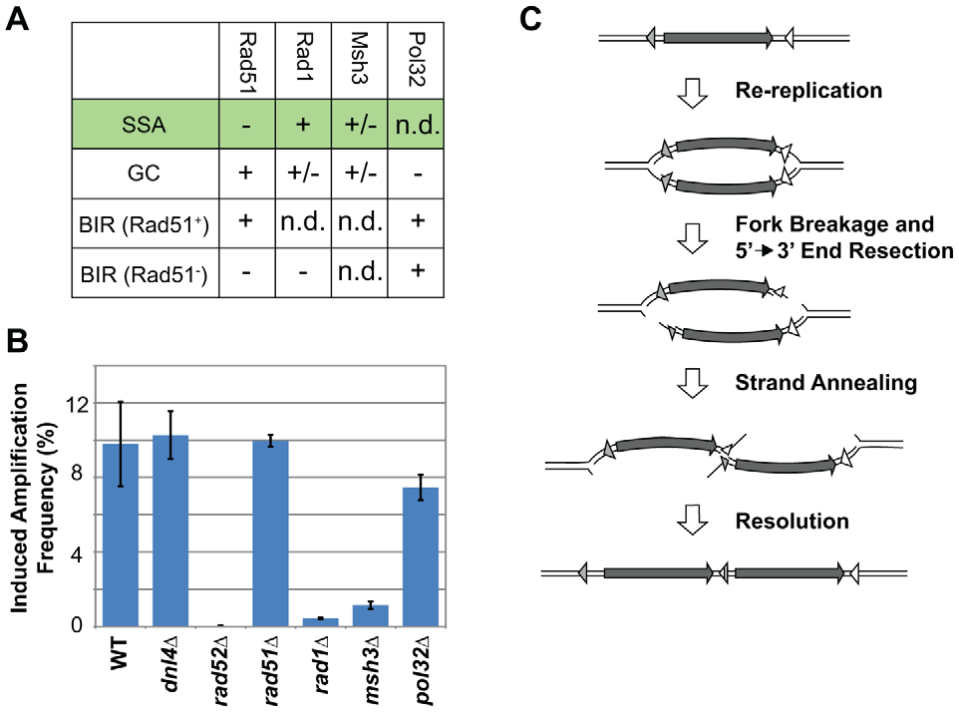
RRIGA is primarily mediated by single-stranded annealing (SSA). A) Summary of genetic requirements for the major sub-types of homologous recombination. SSA = Single Stranded Annealing; GC = Gene Conversion; BIR = Break Induced Replication. “+” = required; “−” = not required; “+/−” = required in some but not all cases; “n.d.” = not determined. B) RRIGA amplification frequencies for WT (YJL8363/8364), *dnl4Δ* (YJL8407/8408), *rad52Δ* (YJL8409/8410); *rad51Δ* (YJL8412/8413), *rad1Δ* (YJL8415/8416), *msh3Δ* (YJL8418/8419), and *pol32Δ* (YJL8421/8422) strains using the *URA3* selection assay. Difference in frequency after 3 hr and 0 hr induction of re-replication was normalized for differences in the amount of re-initiation (see Text S1 and Figure S4). Data are presented as the mean ± combined error (see Text S1). C) Model for RRIGA involving SSA-mediated NAHR. Arrow, amplified segment. Triangles, non-allelic homologous sequences.

Three major forms of homologous recombination have been characterized in budding yeast and shown to have distinct genetic dependencies (Figure 3A): gene conversion (GC), break induced replication (BIR), and single-strand annealing (SSA) [37]. BIR can be further subdivided into a form that requires the RecA homolog Rad51 and one that is independent of Rad51 [38]. We could thus narrow down the form of homologous recombination responsible for RRIGA by using the *URA3* selection assay to quantify the dependence of RRIGA on various recombination genes. Because the frequency of RRIGA is dependent on the amount of induced re-replication, we normalized the measured frequency against the height of the induced re-replication peak (Figure S4A)

The genetic dependencies for RRIGA most closely resembled those for SSA (Figure 3B, Table S3). First, RRIGA was independent of Rad51, which is required for strand invasion in GC and Rad51-dependent BIR but is not required for SSA [39]–[44]. Second RRIGA was dependent on Rad1 and Msh3. The former functions as part of the Rad1-Rad10 structure specific endonuclease, which removes non-homologous 3′ tails during SSA. The latter functions as part of the Msh2-Msh3 complex to stabilize the SSA structure that is recognized by Rad1-Rad10 [45]–[47]. Finally, RRIGA was independent of Pol32, a non-essential subunit of DNA Polymerase δ that is important for BIR [48]. Similar results for *RAD51* and *RAD1* were observed using the colony sectoring assay, although a partial dependence on Rad51 suggests that a subset of these RRIGA events may require this protein (Figure S4B, S4C, Table S1). Taken together, these results indicate that most of the NAHR observed in RRIGA is mediated by SSA.

Such a central role for SSA both restricts the possible mechanisms for RRIGA and expands the genetic alterations associated with SSA. SSA is almost always associated with deletion of chromosomal segments that lie between flanking homologous sequences [37]. A break between those sequences followed by 5′ end resection past both sequences allows them to anneal and initiate repair through NAHR, but at the cost of deleting the intervening segment. In the context of a re-replication bubble, however, SSA could generate a tandem duplication if both forks of the bubble travel beyond flanking homologous sequences and break in *trans* relative to the chromosome axis. The dual fork breaks would cleave the re-replicated sister chromatid in two, leaving a copy of its re-replicated portion at each broken end. Subsequent 5′ end resection back toward the re-replicated homologous sequences closest to each end would then expose complementary strands of these non-allelic sequences for annealing and SSA repair, resulting in a head-to-tail tandem duplication *in loco* (Figure 3C). Such a model provides the most straightforward explanation for how SSA can be responsible for RRIGA. Moreover, in this model, the special context provided by the re-replication bubble to exploit SSA for tandem duplications suggests one reason why re-replication is such a potent inducer of gene amplification.

### Re-Replication Generates DSBs Distal to Both Flanking Repetitive Elements

A key requirement in our SSA model for RRIGA is that each re-replication fork must break origin-distal to the homologous sequence element that will undergo NAHR (Figure 3C). Although re-replication is known to induce double-strand breaks (DSBs), or at least a robust DNA damage response, in most cases the source of those breaks is unknown and actual breakage of re-replication forks has not been directly implicated [24]–[31]. We therefore asked whether there is a correspondence between the position of DSBs and re-replication forks and whether DSB do in fact arise distal to both flanking repetitive elements.

**Figure 4:**
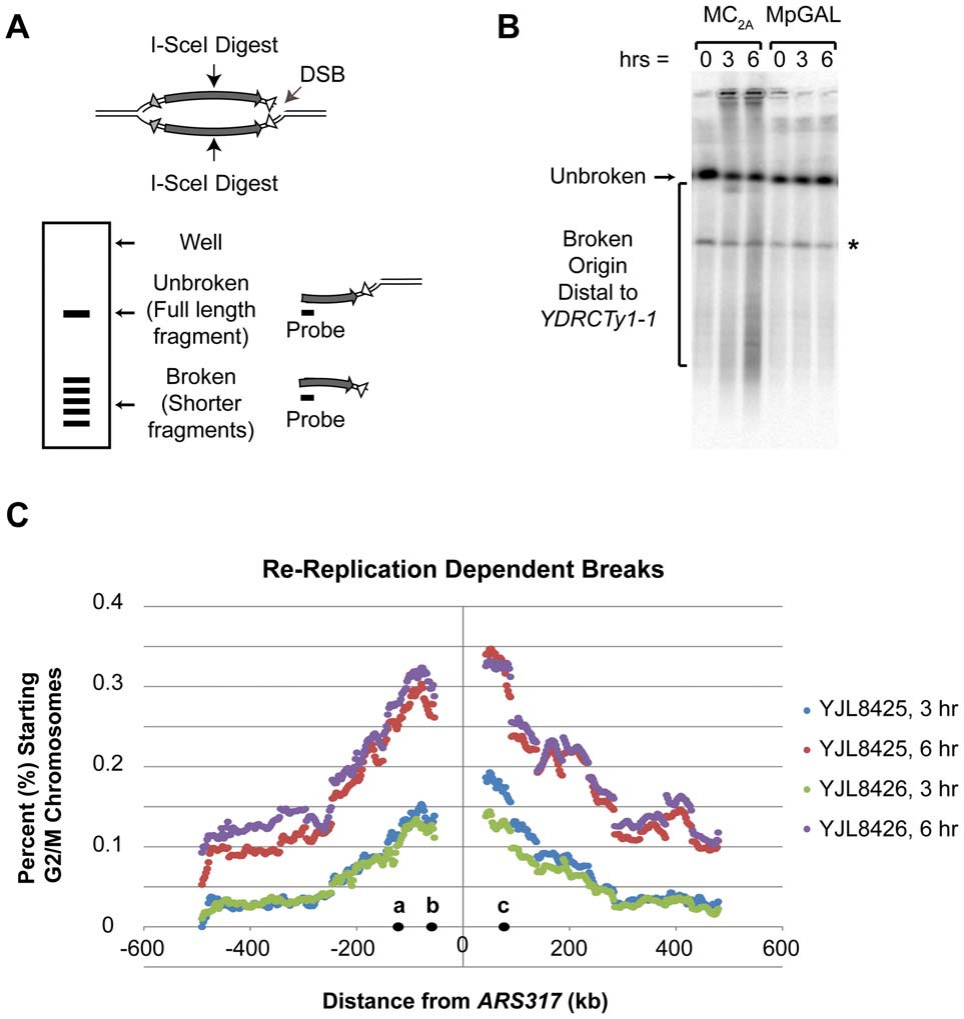
Re-replication induces double-stranded DNA breaks distal to flanking repetitive elements. A) Strategy for mapping DSBs arising during re-replication using an I-SceI site near the re-initiating origin *ARS317* as a physical reference point (see Text S1). The lengths of the small linear fragments generated by DSBs indicate the position of the DSBs relative to the I-SceI cut site. B) Representative Southern blot for DSBs induced by rightward moving re-replication forks. Re-replicating MC_2A_ strains (YJL8425/YJL8426) were induced to re-replicate and at the indicated times chromosomal DNA was prepared, digested with I-SceI, size-separated by PFGE, Southern blotted, and probed for fragments extending rightward from the I-SceI site. Genomic DNA from non-re-replicating Mp_GAL_ control strains (YJL8427/8428) were processed in parallel. Unbroken: full-length Chromosome IV fragment from I-SceI site to right telomere. Bracket: fragments due to DSBs that map origin-distal to YDRCTy1-1. * unidentified DNA fragment present independent of re-replication. C) Distribution of DSBs induced by re-replication from *ARS317* (see Text S1 and Figure S5). For each 2425 bp size range, the amount of re-replication induced fragmentation within that size range is displayed as a percent of the total amount of G2/M chromosomes before re-replication was induced. Positions are relative to *ARS317* (at 0 kb) with positions of CEN4 (a), *YDRCTy2-1* (b), and *YDRCTy1-1* (c) indicated.

To map the location of DSBs that arise during re-replication from *ARS317*, we sized chromosomal fragments generated by these breaks using pulsed field gel electrophoresis (PFGE) (Figure 4, Figure S5). By preparing genomic DNA from cells embedded within agar plugs, this technique minimizes breakage from *in vitro* manipulations. Cells were harvested for PFGE after inducing re-replication for 0, 3, or 6 hr; as a control we harvested cells from a congenic non-re-replicating strain at the same time points. Prior to PFGE, chromosomal DNA was digested with the I-SceI endonuclease, which cuts a single unique I-SceI recognition site engineered very close to *ARS317*. This digest divides Chromosome IV into two fragments containing sequences to the left and right of *ARS317*, respectively. For those molecules that re-initiated from *ARS317*, the digestion will convert the resulting bubble intermediates into left and right Y-shaped chromosome fragments with *ARS317* near the arm tips, telomere at the stem base, and re-replication fork at the branch point. Hence, a DSB in the re-replicated segment will cleave off an arm of the Y, generating a truncated chromosomal fragment whose length defines the position of the break relative to *ARS317* (Figure 4A). After size separation by PFGE, these fragments were detected by southern analysis using probes just to the left or right of the I-SceI cut site (Figure 4B, Figure S5).

Using this approach, we found that re-replication dependent DSBs did indeed arise with significant frequency on both sides of *ARS317* (Figure 4B, Figure S5). Full-length right and left fragments from I-SceI-digested Chromosome IV were detected as discrete bands. Truncated fragments arising from DSBs migrated as a smear representing a range of sizes below the full-length fragments. These truncated fragments were specific to the re-replicating strain and became more abundant with longer induction of re-replication. Quantifying the amount of each fragment length relative to the starting amount of G2/M chromosomes before re-replication (0 hr) allowed us to estimate the percent of these chromosomes that acquired a DSB at each chromosomal position as a consequence of re-replication (see Text S1). Figure 4C shows a plot of this DSB percentage as a function of distance from *ARS317*. The distribution formed a broad peak centered about the origin similar to the distribution of re-replication forks around *ARS317* (Figure S4A: WT). The similarity of these distributions is consistent with the notion that the DSBs arise from breakage of re-replication forks.

Importantly, many of the DSBs we mapped arose origin-distal to the two flanking repetitive Ty elements that are closest to *ARS317* and that participate most frequently in RRIGA. We suspect our analysis undercounts DSB formation because some re-replication forks may not break until after the re-replication induction period, and some forks that break early in this period may already have been repaired. Nonetheless, the data provide a ballpark estimate of the percent of G2 chromosomes that acquire a double strand break beyond the most proximal Ty element as a consequence of re-replication. After 3 hr of re-replication this estimate is roughly 10–15% for either side of *ARS317* (see Text S1). After 6 hr of re-replication the estimate is roughly 30–45%. Thus, these breaks are not rare, and there is a reasonable probability that a re-replication bubble will break at both forks at the positions needed to stimulate the use of homologous sequences in our model for SSA-mediated RRIGA.

### Re-Replication Forks Must Proceed beyond Flanking Repetitive Elements for RRIGA

**Figure 5:**
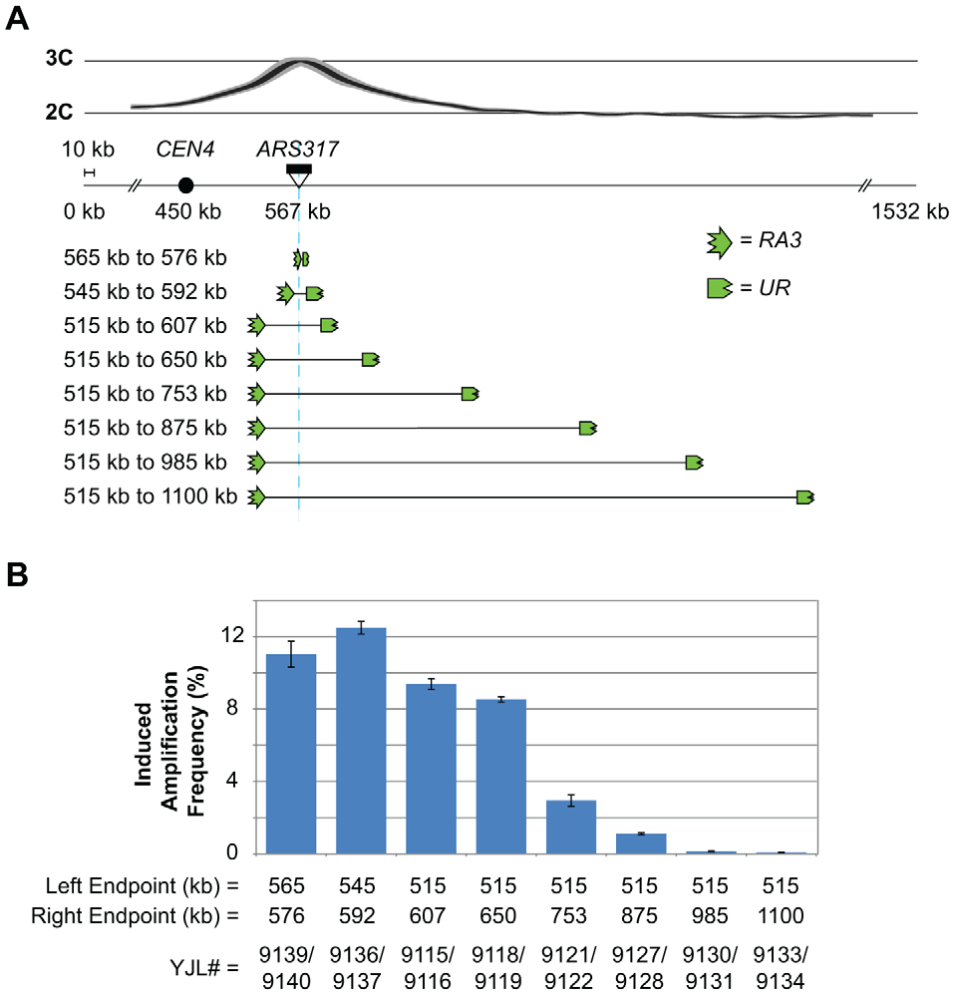
Flanking repetitive elements must be re-replicated in order for RRIGA to occur. A) Schematic showing relocation of the *RA3* and *UR* elements (3′ and 5′ portions of *URA3*, respectively) used in the *URA3* RRIGA selection assay to change their position relative to the re-initiating origin (*ARS317*, light blue line) and the distribution of re-replication forks (as inferred from the re-replication profile). B) Induced amplification frequencies (mean ± SEM, n = 3) for strains with the indicated amplicon boundaries as defined by the position of the *URA3* fragments. Induced frequency is frequency after 3 hr re-replication minus frequency after 0 hr re-replication.

Our model predicts that the further away a homologous sequence is from the origin, the lower the frequency of RRIGA involving that sequence, as fewer re-replication forks will be able to reach that sequence and break beyond it. The fact that RRIGA amplicons preferentially arise from NAHR between the two closest Ty elements flanking the re-initiating origin as endpoints is consistent with this prediction. However, to test this prediction directly we used the *URA3* selection assay to quantify the frequency of RRIGA in a series of strains where the flanking *URA3* fragments were placed at increasing distance from *ARS317* (Figure 5A).

The overall trend supports the prediction. As the flanking URA3 fragments are moved further away from *ARS317*, RRIGA frequencies drop (Figure 5B, Table S3). RRIGA isolates from each starting strain were examined by aCGH to confirm that their amplicons did indeed extend from one *URA3* fragment to the other (Figure S6A, Table S4). Importantly, despite the large range of amplicon sizes (11 kb to 585 kb) there was no detectable difference in the growth rates of these isolates (Figure S6B). Hence, the decrease in RRIGA frequencies cannot be explained by a decrease in fitness of those RRIGA isolates with larger amplicons. Instead these results support the requirement for forks to replicate and break beyond flanking homologous sequences.

### RRIGA Proceeds Most Efficiently with the Re-Initiating Origin within the Amplicon

In the SSA model of RRIGA, after a re-replication fork breaks origin-distal to a homologous sequence element, 5′ end resection from the break must proceed back to the homologous sequence to make it available for SSA. With resection rates in *S. cerevisiae* estimated at 4 kb per hour [49], breaks that arise tens of kilobases past the homologous sequence will require many hours of resection before they can facilitate RRIGA, increasing the likelihood that the break will be repaired by an alternative mechanism or fail to occur before chromosomes finally segregate. Hence, one might expect some constraint on how far a fork break can occur beyond a homologous sequence and still stimulate the use of that sequence for RRIGA.

**Figure 6:**
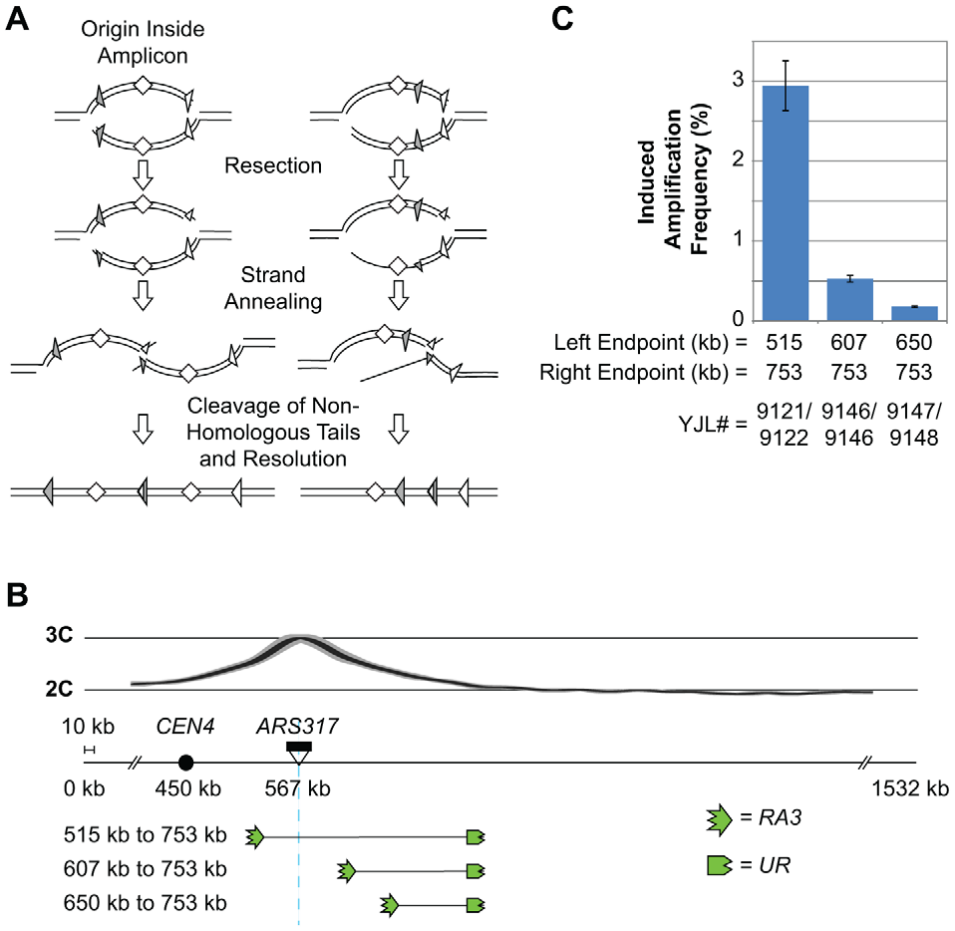
RRIGA proceeds most efficiently when the re-initiating origin lies within the amplicon. A) Schematic comparing SSA models for RRIGA when the re-initiating origin is within the amplicon versus when the origin is outside. The latter case requires long range strand resection back beyond the re-initiating origin in order to expose homologous sequences for NAHR. B) Schematic showing relocation of the *RA3* element to change its position relative to the re-initiating origin (ARS317, light blue line). The re-replication profile is shown above. C) Induced amplification frequencies (mean ± SEM, n = 3) for strains with the indicated amplicon boundaries. Induced amplification frequency calculated as described for Figure 5B.

Such a constraint would influence the optimum position of a re-initiating origin relative to homologous boundaries of a potential amplicon. One would predict that RRIGA should be more efficient when the origin is within the amplicon than when it is outside (see Figure 6A). In the latter case, any distance traveled by the re-replication fork that initially moves away from the amplicon will have to be completely retraced during resection followed by further resection from the origin to the closest boundary.

To test this prediction, we generated a series of re-replicating strains in which the right amplicon boundary was held fixed while the left amplicon boundary lay either to the left of *ARS317* (positioning the origin within the amplicon) or at two sites to the right of *ARS317* (positioning the origin outside of the amplicon) (Figure 6B). In this series, the rightward re-replication fork has to travel the longest distance to reach the right homologous sequence boundary, and this distance is unchanged. In contrast, the leftward re-replication fork has little or no distance to travel to get past the left homologous sequence boundaries, but wherever it might break the resection distance back to those boundaries increases. In accordance with the prediction, the RRIGA frequency tracks inversely with the anticipated resection distance. The frequency is highest for the strain with the origin contained within the amplicon and becomes progressively lower as the left amplicon boundary is positioned further to the right of the origin (Figure 6C, Table S3).

Because the size of the amplicons varied in this series of strains, we also compared strains with relatively constant amplicon size in which the re-initiating origin was effectively repositioned outside of the amplicon (Figure S7A). In one set of strains, amplicon boundaries approximately 140 kb apart were moved to the right of *ARS317*, causing a precipitous drop in RRIGA (Figure S7B, Table S3). Although part of this drop can be attributed to the increased distance re-replication forks have to travel to the rightmost boundary (see Figure 5B, strains YJL9118/9119 versus strains YJL9121/9122), the remainder is likely due to the repositioning of the origin outside of the amplicon (compare Figure S7B strains YJL9145/9146 versus Figure 5B strains YJL9121/9122). Similarly, in strains where the amplicon size is maintained at ∼100 kb there is a dramatic decrease in RRIGA frequency when the origin is positioned outside of the amplicon (see Figure S7B, strains YJL9115/9116 versus YJL9147/9148). Thus, as expected from the SSA model for RRIGA, a re-initiating origin is most efficient at inducing amplification if the origin lies between the homologous sequences that define the amplicon boundaries.

## Discussion

### A Model for Re-Replication Induced Gene Amplification

We have previously shown that re-replication in budding yeast is remarkably efficient at inducing NAHR events that result in tandem gene amplifications oriented in direct repeat [18]. A transient, localized, limited pulse of re-replication from a single origin induced segmental amplifications on the order of 10^−2^ per cell per generation. This efficient amplification appeared to be specific to re-replication, as disruption of S-phase replication with mutant replication proteins or hydroxyurea did not induce equivalent amplification frequencies. In this paper, we propose a model for re-replication induced gene amplification (RRIGA) that helps explain why this amplification is so efficient and that provides a new mechanism for NAHR-mediated copy number variation. Such efficiency makes it conceivable that rare or sporadic re-replication events might contribute to DNA copy number changes observed during oncogenesis or evolution.

In our model for RRIGA (Figure 3C), bidirectional re-replication forks proceeding outward from a re-initiating origin can stimulate an efficient NAHR event between flanking homologous sequence elements by replicating beyond them and generating DSBs. Normal processing of these breaks will involve 5′ to 3′ single-strand resection back toward the homologous sequences. In those cases where the two forks break in *trans*, this resection can expose complementary sequences in non-allelic homologous sequences, resulting in annealing and repair of the break by an SSA mechanism. The result is a head-to-tail tandem duplication at the endogenous chromosomal locus. Such tandem duplications can provide a stepping stone for higher order amplifications [50]. Expansion of the duplication might occur readily without further re-replication, as the initially duplicated segments provide a much larger NAHR substrate. On the other hand, if re-replication were to recur in subsequent generations, it could stimulate a series of stepwise expansions that would lead to multi-copy amplification.

### Re-Replication Fork Breakage Drives RRIGA

An important premise for our RRIGA model is the ability of re-replication to induce frequent chromosomal breaks. There are many reports associating the deregulation of replication initiation proteins with the generation of chromosomal breaks or the induction of a DNA damage response [24]–[31]. However, in most cases, this deregulation has been imposed constitutively throughout the cell cycle, making it hard to distinguish whether these breaks are due to re-replication per se or arise from possible disruption of S-phase replication. Because we induced re-replication after completion of an intact S-phase, the chromosomal breaks we observed and mapped can be specifically attributed to re-replication. Importantly, the correspondence between the distribution of breaks and the distribution of re-replication forks along the chromosome suggests that these forks are the source of these DSBs.

Formally, it is possible that the DSBs we mapped were actually the free ends of newly synthesized DNA fragments extruded by head-to-tail fork collisions during multiple rounds of re-replication, as has been proposed to explain induction of a DNA damage response during re-replication in Xenopus extracts [51]. However, this scenario is unlikely in our gene amplification studies, where we induced on average only half a round of re-replication near *ARS317* (i.e. copy number increase from 2C to 3C). Hence, it appears that the re-replication forks themselves are breaking, leading to chromosome fragmentation.

The distribution of re-replication-induced breaks did not reveal any striking hotspots, indicating that these breaks do not depend on special DNA elements or structures that are suspected of potentiating DSB formation by promoting fork stalling and/or collapse [52]–[54]. The independence of these breaks from the inverted LTR repeats and multiple tRNA genes that often surround Ty elements is consistent with our ability to replace entire clusters of these elements with simple homologous sequences and still observe high frequency RRIGA.

Our results therefore raise the possibility that re-replication forks are particularly susceptible to breakage. Supporting this notion is our previous observation that the induction of re-replication can lead to a rapid and massive Rad9-dependent DNA damage response, a response that is not seen during unperturbed S-phase [24]. In fact, even when S-phase was subjected to prolonged disruption from hydroxyurea, it did not generate the type of chromosome fragmentation that was readily detected during re-replication [24]. Hence, unlike S-phase, where breakage among thousands of replication forks is a rare accident, during re-replication fork breakage may be the rule rather than the exception.

Clearly, an important future question will be why forks are so susceptible to breakage during re-replication. Nonetheless, the fact that they are increases the likelihood that re-initiation will lead to a bubble with a break at both forks. Our rough order of magnitude estimate of chromosome breakage frequencies on a single side of *ARS317* (10–15% after 3 hr of re-replication and 30–45% after 6 hr) suggests that such dual fork breaks occur with significant frequency. Thus, even the slightest amount of re-replication may be a potent source of copy number variation.

### The Structural Context Provided by the Re-Replication Bubble Also Facilitates RRIGA

Another key feature of our model that contributes to the efficiency of RRIGA is the structural context provided by the re-replication bubble. First, this structure provides an extra, non-essential chromosomal segment in close proximity to the endogenous segment. Second, when both forks happen to break in *trans* relative to the chromosome axis, this structure channels recombinational repair of the broken ends toward the formation of tandem duplications. Consistent with the importance of this structural context, simply inducing DSBs alone is insufficient to induce amplifications in our assay [18]. DSBs have been implicated in promoting NAHR events that result in the formation of deletions, translocations, and isochromosome formation, but they rarely lead to the type of intrachromosomal amplifications so efficiently induced by re-replication [10], [55]. DSBs are also capable of initiating a mechanism of gene amplification referred to as breakage-fusion-bridge (BFB), but the resulting amplification structure is very different from RRIGA, with amplicons oriented in inverted repeat and a terminal deletion beyond the amplified locus [56]–[59]. Thus, without the context of the re-replication bubble, DSBs do not show the same propensity for forming tandem duplications as observed during RRIGA.

The broader context in which re-replication bubbles appear may also contribute to the efficiency of RRIGA. The multiple overlapping layers of replication controls used by eukaryotic cells [18]–[20] ensure that only a limited number of origins will re-initiate when some of these controls are disrupted. The isolation of the resulting re-replication bubbles increases the likelihood that both forks of a bubble will eventually stall as they run into problems or simply reach the limits of their processivity. Without converging forks from nearby re-replication bubbles to rescue them, these stalled forks will be even more susceptible to breakage. Thus, we anticipate that a large proportion of re-initiation events will form the broken bubble intermediate that can be channeled into tandem direct amplifications.

In contrast, although S-phase replication bubbles are structurally identical to re-replication bubbles, the redundancy of active and backup origins in S-phase [60], [61] ensures that replication bubbles rarely arise in isolation and any fork that happens to stall is readily rescued by a converging fork from a neighboring replication bubble. This may explain why, despite the proposed link between S-phase accidents and various genomic rearrangements [62]–[64], inhibiting origin function or stressing S-phase forks throughout the genome with mutant replication proteins or DNA synthesis inhibitors does not generate RRIGA-like amplification frequencies [18]. To generate isolated replication bubbles, the origin failure or fork stress would have to be so severe that replication would be catastrophically and lethally disrupted. Examples of more tolerated localized replication fork stress have been identified through the discovery of common fragile sites (CFS) [65], [66]. However, individual CFSs can only affect one of the two forks from an expanding replication bubble and, hence, would not generate the broken bubble intermediate central to our RRIGA model. Consistent with this expectation, although some CFSs have been associated with amplifications, the amplifications are not arranged in tandem direct repeat like RRIGA. Instead they are arranged as inverted repeats and are thought to arise through a BFB mechanism initiated by a CFS break [67]–[69].

There is one example of a mutation that disrupts S-phase replication and induces segmental duplications structurally similar to RRIGA amplifications. Deletion of *CLB5*, one of the S-phase cyclins that triggers origin firing in budding yeast, reduces or delays origin activity primarily in the later replicating regions of the genome [70]. This deletion significantly stimulates tandem direct duplications involving NAHR at one chromosomal locus [17]. The mechanism by which *clb5Δ* stimulates these duplications is unknown, but whether or not it occurs through a RRIGA-like mechanism, the duplication rate is still more than one hundred fold lower (7.3×10^−5^ per cell division [17]) than the rate of RRIGA induced by *ARS317* re-initiation. Altogether, these observations suggest that origin re-initiation may be particularly efficient at inducing amplifications in direct repeat because it is particularly efficient at generating isolated re-replication bubbles broken at both forks.

### A Wider Set of Amplification Structures from Re-Replication?

In principle, once re-replication has generated re-replication bubbles broken at both forks in *trans*, subsequent formation of tandem direct duplications can occur by a variety of available repair mechanisms. In budding yeast, where homologous recombination predominates, we have shown that this repair occurs primarily through SSA-mediated NAHR. However, in other organisms, repair could conceivably proceed through alternative mechanisms that don't require extensive sequence homology, such as non-homologous end-joining (NHEJ) or microhomology mediated end-joining (MMEJ). Interestingly, numerous tandem duplications with little or no sequence homology at their interamplicon junctions have been observed in both normal and cancerous human genomes [16], [71], [72]. Hence, we are currently investigating whether re-replication can induce these types of duplications as well.

One can also imagine that if both forks of a re-replication bubble break, they may often break in *cis*, releasing one arm of the re-replication bubble and a full length chromosome. In that event, re-circularization of the arm, whether by SSA-mediated NAHR or some other repair mechanism, would generate a circular extrachromosomal amplicon, such as those frequently seen in human tumors [73]. We did not see evidence of extrachromosomal amplification in our system (Figure 2D), but our amplicons lacked centromeres, and in budding yeast, such amplicons would both be very unstable and preferentially accumulate to high, potentially toxic, copy number in mother cells [74]. It will thus be interesting to see if re-replication can also efficiently stimulate extrachromosomal amplification of centromere containing segments, such as the amplifications reported by Libuda and Winston [75].

### Implications for Disease and Evolution

Our work provides a basis for understanding why re-replication arising from the loss of replication controls can generate duplications and amplifications with such remarkable efficiency. A much harder question to address is *does* re-replication contribute to gene amplifications and tandem duplications, such as those observed in cancers? Three observations suggest that this question is worth pursuing. First, dysregulation of replication initiation proteins has been observed in human cancer cells [4], [76]–[81]. Second, overexpression of replication initiation proteins in certain murine models can promote oncogenesis [81]–[83]. Third, a number of oncogene amplifications display amplicon structures with some or all of the features of yeast RRIGA structures: direct repeat, at the endogenous chromosomal locus, bounded by homologous sequence elements [84]–[87]. More recently, cancer genome sequencing efforts have detected the appearance of numerous tandem duplications in certain cancers [72]. Despite the lack of significant sequence homology at many of their boundaries, these duplications could still conceivably arise by some form of RRIGA, as discussed above. Importantly, the most popular model for gene amplification, the breakage-fusion-bridge mechanism [56], [57], cannot explain these amplifications/duplications in direct repeat, creating a need for alternative mechanisms.

Recently, it has been suggested that the deregulation of replication initiation might promote oncogenesis more directly through the induction of replication stress, resulting in extensive fork stalling, fork collapse, and DSBs [88]–[91]. Although the exact nature or source of this replication stress is not clear, our work documenting the extensive DNA damage and DSBs arising from re-replication forks makes re-replication a possible candidate.

Finally, we note that rare spontaneous tandem duplications and/or amplifications involving NAHR have been observed arising in yeast containing intact replication controls [17] (KJ Finn, unpublished results). These observations invite speculation that, despite intact controls, sporadic re-replication might still occur and cause gene copy number expansions that could promote evolution directly by generating phenotypic variation [11] or indirectly by removing constraints on molecular diversification [1]. It thus will be of interest to see whether these spontaneous copy number gains share some of the genetic dependencies of RRIGA established in this work.

## Materials and Methods

### Strains

All strains used in this study are listed in Table S5 are derived from YJL6974 and YJL6558 [18] using standard methods. Details of their construction, along with the plasmids (Table S6) and oligonucleotides (Table S5) used in their derivation, can be found in the Supplemental Material.

### Strain Growth and Induction of Re-Replication

Yeast cells were grown as previously described [18]. Induction of re-replication was performed as previously described [18]. Full details are available in the Supplemental Material.

### Colony Sectoring Assay

The colony sectoring assay was performed as previously described [18]. Full details are available in the Supplemental Material.

### Uracil Prototrophy Assay

Following induction of re-replication ∼5,000 cfu were plated onto each SDC-Ura plate (to isolate uracil prototrophs) and ∼250 cfu onto each SDC plate (to determine an accurate cfu plated onto the SDC-Ura plates). Plates were incubated at 30°C for 3–5 days, then colonies were counted. The frequency of uracil prototrophs was determined by dividing the total number of colonies on the SDC-Ura plates by the number of cfus plated on the SDC-Ura plates. This frequency was measured in at least two independent experiments and the mean and standard error of the mean (when 3 or more trials were conducted), or the mean and the standard deviation (when only 2 trials were conducted) are reported.

### Pulsed-Field Gel Electrophoresis and Southern Blotting

Cells were fixed and embedded in agarose for PFGE essentially as described [24], except that a proteinase K inactivation step with PMSF was included at the end of plug preparation. 1/3 of each plug was then treated with I-SceI to digest the embedded chromosomal DNA. Plugs were then loaded on a 1% SeaKem LE agarose (wt/vol) gel in 0.5× TBE. The gel was electrophoresed in 14°C 0.5× TBE on a CHEF DR-III system (Bio-Rad) with initial switch time of 50 sec, final switch time of 95 sec, run time of 26 hr, voltage of 6 V/cm, and angle of 120°. The DNA was transferred essentially as described [24], except UV-nicking was used instead of acid hydrolysis. The membrane was probed with a *MAK21* probe generated by PCR from yeast genomic DNA with oligonucleotides OJL2449 and OJL2450, and a *YOS9* probe generated by PCR from yeast genomic DNA with oligonucleotides OJL2231 and OJL2232. A Lambda probe was used to detect a sizing ladder. Images were collected using a Typhoon 9400 (GE Healthcare). Data analysis was carried out using Image J (NIH) and Excel (Microsoft Corp.) software. Full details are available in the Text S1.

### aCGH

DNA used for aCGH was prepared as essentially described [18], [92]. Labeling, hybridization, data acquisition, and data analysis were performed as described [18]. Full details are available in the Supplemental Material.

### Junction PCR

Primers used for junction PCR to determine amplicon orientation and preservation of parental junctions are listed in Table S7. PCR was performed using Phusion DNA polymerase (Finnzymes) according to the manufacturer's instructions. DNA used for junction PCR was prepared using a spheroplasting mini-prep method. Full details are available in the Supplemental Material.

### Accession Numbers

All arrayCGH data from this study have been deposited in the Gene Expression Omnibus (GEO) (http://www.ncbi.nlm.nih.gov/geo) database (Series Accession Number GSE41259).

## Supporting Information

Figure S1 Detailed schematics of *YDRCTy1-1*, *YDRCTy2-1*, and *URA3* gene fragment replacements. Schematic comparing the endogenous Ty elements to two versions of the *URA3* fragment replacements. A) Zoomed in view of *YDRCTy2-1* and *YDRCTy1-1*, along with the nearby LTRs and tRNA genes. The 1.3 kb region of 99% sequence identity shared by the two Ty elements is boxed in green. B) Zoomed in view of Version 1 of the *URA3* fragment replacements. The core Ty elements and some of the LTRs are replaced, but the tRNA genes and an inverted LTR repeat are undisturbed. The 390 bp of overlapping sequence identity is boxed in green. C) Zoomed in view of Version 2 of the *URA3* fragment replacements. All of the tRNA genes and LTRs shown in (A) are deleted by these *URA3* fragment replacements. The 390 bp of overlapping sequence identity is boxed in green.
(PDF)

Figure S2 RRIGA requires homology in *cis* and is not enhanced by the presence of inverted LTR repeats or tRNA genes. A) Sectoring frequencies for strains with the endogenous Ty elements at 515 kb and 650 kb (YJL8100), with *YDRCTy2-1* replaced by *RA3* (YJL8104), with *YDRCTy1-1* replaced by *UR* (YJL8108), or both Ty elements replaced (YJL8112). The *URA3* gene fragment replacements used here are Version 1 (see Figure S1). The sectoring frequencies before (0 hr) and after (3 hr) induction of re-replication are shown. Data are presented as the average ± SD of 2–5 trials for each strain. B) Comparison of amplification frequencies for the Version 1 (YJL8112/8113) and Version 2 (YJL8363/8364) *URA3* fragment replacements using the uracil prototrophy selection assay. A non-re-replicating strain (Mp_GAL_ = YJL9149-9151) is also included as a control. The amplification frequencies before (0 hr) and after (3 hr) induction of re-replication are shown. Data are presented as the average ± SD of 2–5 trials for each strain.
(PDF)

Figure S3 aCGH analysis of selected isolates from the sectoring assay. A subset of the post-induction (3 hr) isolates from the sectoring assay presented in Figure 1 were analyzed using aCGH. Representative aCGH profiles are shown with a tally of how frequently each profile was observed for each strain. *Ty-Ty* = YJL8100; *RA3-Ty* = YJL8355; *Ty-UR* = YJL8359; *RA3-UR* = YJL8363. Chromosome IV schematic shows positions of Ty elements (triangles, also showing orientation), centromere (circle), and *ARS317-ade3-2p* re-initiation cassette (bar and vertical line).
(PDF)

Figure S4 RRIGA is primarily mediated by single-stranded annealing (SSA). A) Re-replication profiles for strains with mutations in recombination factors. WT = YJL8363/8364; *dnl4Δ* = YJL8407/8408; *rad52Δ* = YJL8409/8410; *rad51Δ* = YJL8412/8413; *rad51Δ* = YJL8415/8416; *msh3Δ* = YJL8418/8419; *pol32Δ* = YJL8421/8422. Re-replication from a 3 hour induction was determined using aCGH for each strain. The black line shows the average from 3 independent trials (4 for wild-type). The thick gray band shows ±1 SD. Chromosome IV schematic shows positions of Ty elements (triangles, also showing orientation), centromere (circle), and *ARS317-ade3-2p* re-initiation cassette (bar). B) Amplification frequencies for various recombination mutants as determined by the sectoring assay. MC_2A_ = YJL6558; MC_2A_ *rad51Δ* = YJL7451; MC_2A_ *rad1Δ* = YJL7445; Mp_GAL_ = YJL6974. The sectoring frequencies before (0 hr) and after (3 hr) induction of re-replication are shown. Data are presented as the average ± SD of 2 independent trials for each strain. C) Corrected amplification frequencies for the 3 hr timepoint. A subset of sector isolates isolated after induction of re-replication for each strain (10, 36, 28, and 4, respectively) were tested by aCGH to determine whether or not there is an amplification including the reporter cassette. The average sectoring frequency was then multiplied by the fraction of aCGH tested isolates bearing an amplification.
(PDF)

Figure S5 Re-replication induces double stranded DNA breaks distal to flanking repetitive elements on both sides of the origin. A) Following digestion with I-SceI, DSBs at each fork can be mapped by PFGE and Southern blotting using a probe that anneals to sequences to the left of the cleavage site (shown in blue) or to the right of the cleavage site (shown in red). B) Mapping DSBs at the leftward moving fork. Unbroken, full length molecules are indicated at the position labeled “1”. Molecules with DSBs that arose origin distal to *YDRCTy2-1* lie within the bracketed area labeled “2”. C) Mapping DSBs at the rightward moving fork. Unbroken, full length molecules are indicated at the position labeled “3”. Molecules with DSBs that arose origin distal to *YDRCTy1-1* lie within the bracketed area labeled “4”. For (B) and (C), two independent trials using sister isolates are shown (MC_2A_ = YJL8425 and YJL8426; Mp_GAL_ = YJL8427 and YJL8428). Breaks are evident in the re-replicating strains (MC_2A_) induced to re-replicate, and these increase in number with increased length of induction. These breaks depend upon re-replication, as they are not observed in the non-re-replicating control strains (Mp_GAL_). The bands indicated with an * are unexplained major species which are not dependent upon re-replication.
(PDF)

Figure S6 Decreased frequency of amplification observed for relocation of the amplicon boundaries is not caused by fitness defects. A) Uracil prototroph isolates for each combination of amplicon boundaries considered in Figure 5, Figure 6, and Figure S7 were analyzed using aCGH. Each isolate bears the expected amplification for the combination of amplicon boundaries present. B) Each isolate shown in panel (A) was tested for growth defects by a serial dilution spot test on SDC-Ura at 30°C (all on the same plate, 5-fold dilutions). All isolates grow with similar fitness.
(PDF)

Figure S7 RRIGA proceeds most efficiently when the re-initiating origin lies within the amplicon. A) Schematic showing relocation of the *RA3* and *UR* elements (3′ and 5′ portions of *URA3*, respectively) used in the *URA3* RRIGA selection assay to change their position relative to the re-initiating origin (*ARS317*, light blue line) and the distribution of re-replication forks (as inferred from the re-replication profile). The upper group of amplicons are of a similar size, comparing a case where the origin is within the amplicon to a case with the origin just outside of the amplicon to a case with the origin at a great distance from the amplicon. The lower pair of amplicons are of a similar size (slightly smaller than the amplicons in the upper group), comparing a case where the origin is within the amplicon to a case with the origin at a very great distance from the amplicon. B) Induced amplification frequencies (mean ± SEM, n = 3) for strains with the indicated amplicon boundaries as defined by the position of the *URA3* fragments. Induced frequency is frequency after 3 hr re-replication minus frequency after 0 hr re-replication.
(PDF)

Table S1 Frequency of 1/2, 1/4, and 1/8 red sectored colonies observed in this work.
(PDF)

Table S2 aCGH analysis of red-sectored colony isolates.
(PDF)

Table S3 Frequency of uracil prototrophs from the selection assay observed in this work.
(PDF)

Table S4 aCGH analysis of uracil prototroph isolates.
(PDF)

Table S5 Yeast strains used in this study.
(PDF)

Table S6 Plasmids used in this study.
(PDF)

Table S7 Oligonucleotides used in this study.
(PDF)

Text S1 Detailed descriptions of all methods and materials used in this study.
(PDF)
