# Supplementary figures and images for: Single-Stranded Annealing Induced by Re-Initiation of Replication Origins Provides a Novel and Efficient Mechanism for Generating Copy Number Expansion via Non-Allelic Homologous Recombination
Source: PLoS Genet. 2013 Jan 3;9(1):e1003192. doi: 10.1371/journal.pgen.1003192 (PMC3536649; doi:10.1371/journal.pgen.1003192)

Figure S1

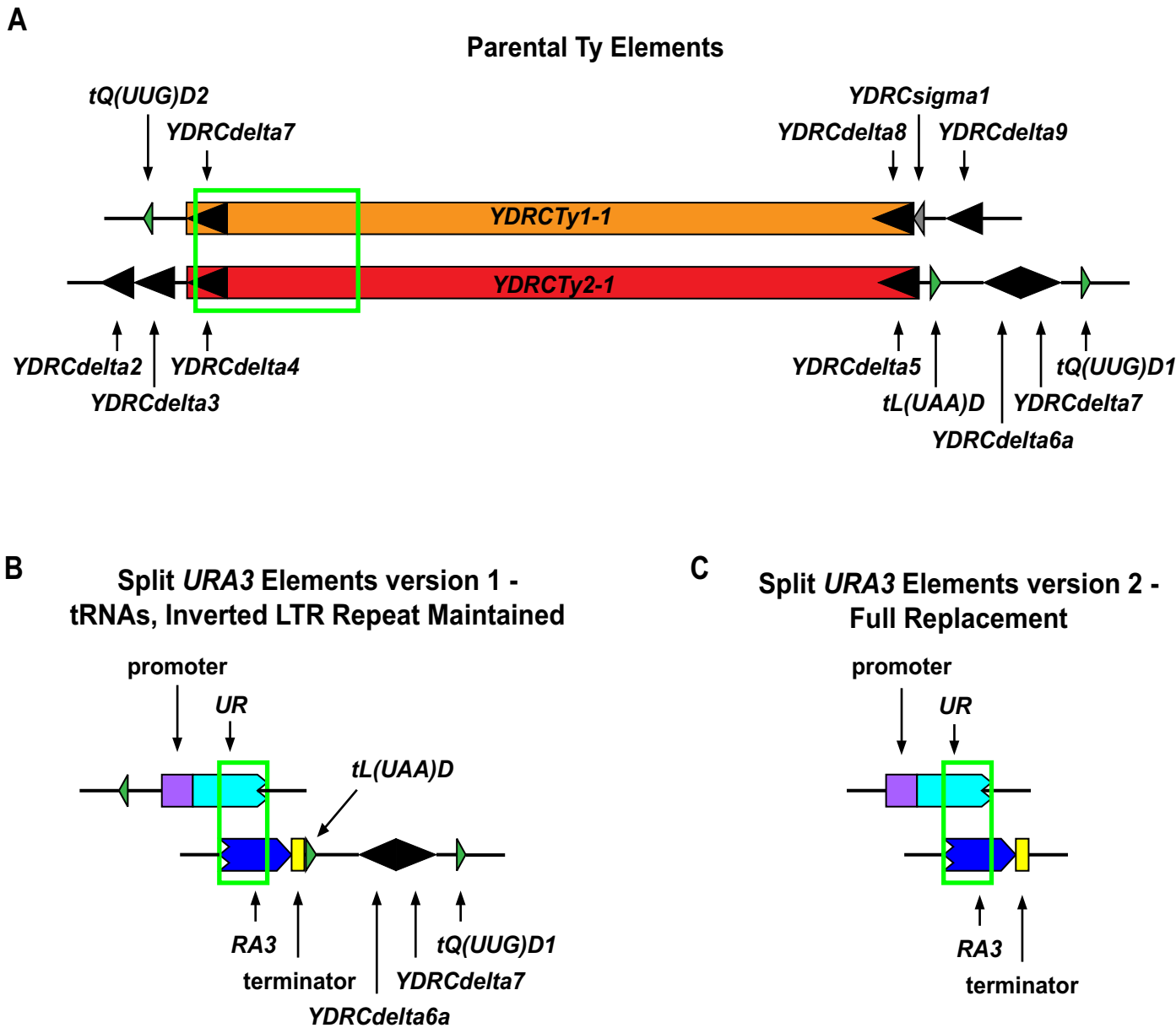

Supplement: Figure S1 — Detailed schematics of YDRCTy1-1, YDRCTy2-1, and URA3 gene fragment replacements. Schematic comparing the endogenous Ty elements to two versions of the URA3 fragment replacements. A) Zoomed in view of YDRCTy2-1 and YDRCTy1-1, along with the nearby LTRs and tRNA genes. The 1.3 kb region of 99% sequence identity shared by the two Ty elements is boxed in green. B) Zoomed in view of Version 1 of the URA3 fragment replacements. The core Ty elements and some of the LTRs are replaced, but the tRNA genes and an inverted LTR repeat are undisturbed. The 390 bp of overlapping sequence identity is boxed in green. C) Zoomed in view of Version 2 of the URA3 fragment replacements. All of the tRNA genes and LTRs shown in (A) are deleted by these URA3 fragment replacements. The 390 bp of overlapping sequence identity is boxed in green. (PDF) [file pgen.1003192.s001.pdf]

Figure S2

A

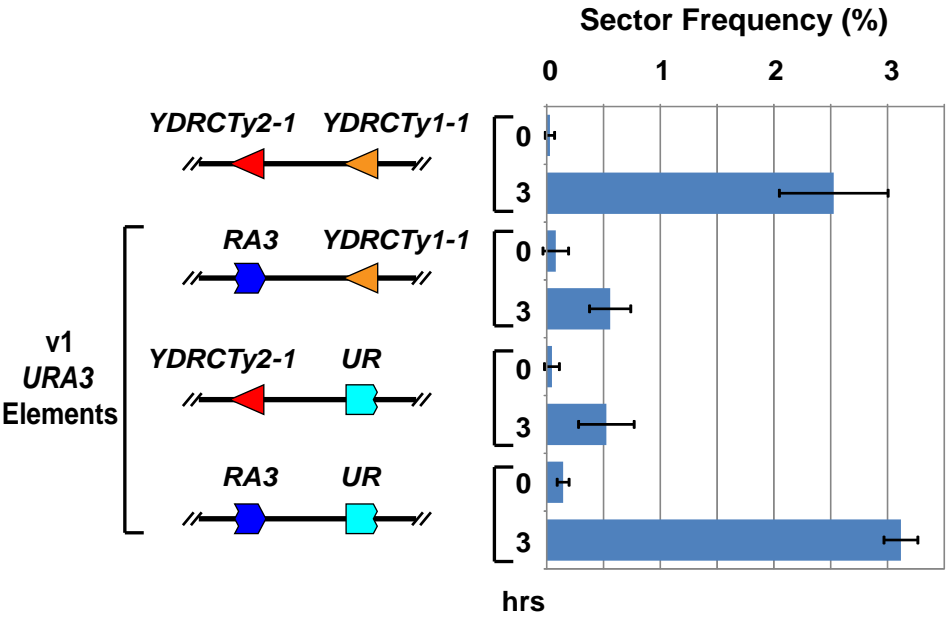

B

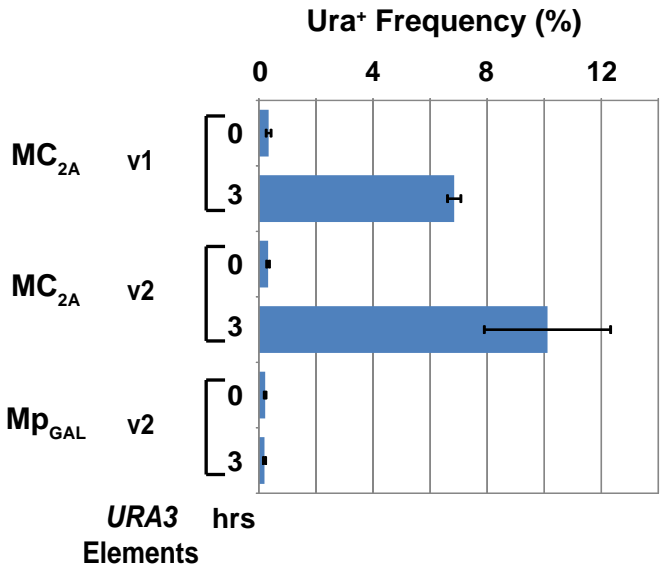

Supplement: Figure S2 — RRIGA requires homology in cis and is not enhanced by the presence of inverted LTR repeats or tRNA genes. A) Sectoring frequencies for strains with the endogenous Ty elements at 515 kb and 650 kb (YJL8100), with YDRCTy2-1 replaced by RA3 (YJL8104), with YDRCTy1-1 replaced by UR (YJL8108), or both Ty elements replaced (YJL8112). The URA3 gene fragment replacements used here are Version 1 (see Figure S1). The sectoring frequencies before (0 hr) and after (3 hr) induction of re-replication are shown. Data are presented as the average ± SD of 2–5 trials for each strain. B) Comparison of amplification frequencies for the Version 1 (YJL8112/8113) and Version 2 (YJL8363/8364) URA3 fragment replacements using the uracil prototrophy selection assay. A non-re-replicating strain (MpGAL = YJL9149-9151) is also included as a control. The amplification frequencies before (0 hr) and after (3 hr) induction of re-replication are shown. Data are presented as the average ± SD of 2–5 trials for each strain. (PDF) [file pgen.1003192.s002.pdf]

Figure S4

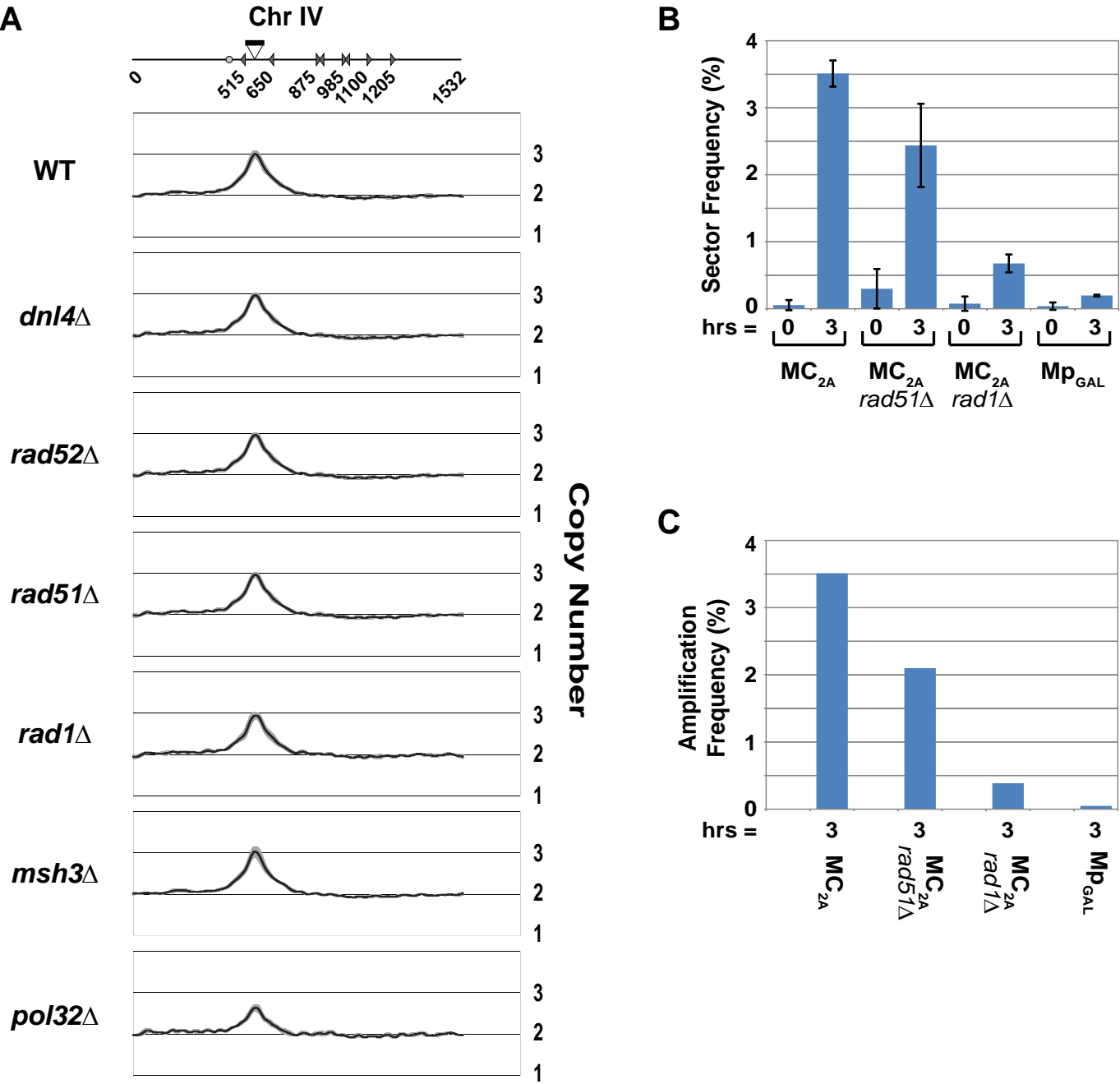

Supplement: Figure S4 — RRIGA is primarily mediated by single-stranded annealing (SSA). A) Re-replication profiles for strains with mutations in recombination factors. WT = YJL8363/8364; dnl4Δ = YJL8407/8408; rad52Δ = YJL8409/8410; rad51Δ = YJL8412/8413; rad51Δ = YJL8415/8416; msh3Δ = YJL8418/8419; pol32Δ = YJL8421/8422. Re-replication from a 3 hour induction was determined using aCGH for each strain. The black line shows the average from 3 independent trials (4 for wild-type). The thick gray band shows ±1 SD. Chromosome IV schematic shows positions of Ty elements (triangles, also showing orientation), centromere (circle), and ARS317-ade3-2p re-initiation cassette (bar). B) Amplification frequencies for various recombination mutants as determined by the sectoring assay. MC2A = YJL6558; MC2A rad51Δ = YJL7451; MC2A rad1Δ = YJL7445; MpGAL = YJL6974. The sectoring frequencies before (0 hr) and after (3 hr) induction of re-replication are shown. Data are presented as the average ± SD of 2 independent trials for each strain. C) Corrected amplification frequencies for the 3 hr timepoint. A subset of sector isolates isolated after induction of re-replication for each strain (10, 36, 28, and 4, respectively) were tested by aCGH to determine whether or not there is an amplification including the reporter cassette. The average sectoring frequency was then multiplied by the fraction of aCGH tested isolates bearing an amplification. (PDF) [file pgen.1003192.s004.pdf]

Figure S5

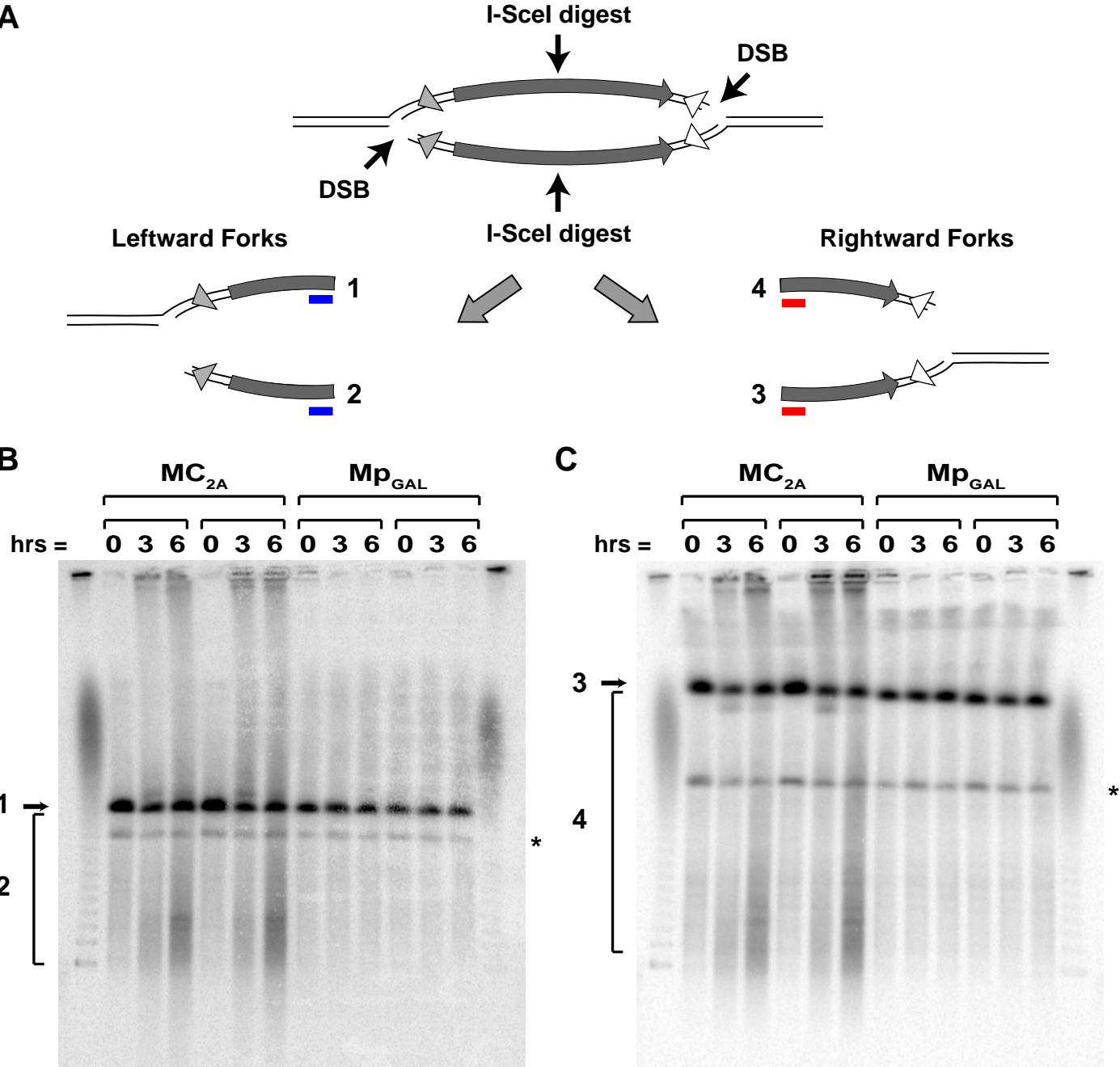

Supplement: Figure S5 — Re-replication induces double stranded DNA breaks distal to flanking repetitive elements on both sides of the origin. A) Following digestion with I-SceI, DSBs at each fork can be mapped by PFGE and Southern blotting using a probe that anneals to sequences to the left of the cleavage site (shown in blue) or to the right of the cleavage site (shown in red). B) Mapping DSBs at the leftward moving fork. Unbroken, full length molecules are indicated at the position labeled “1”. Molecules with DSBs that arose origin distal to YDRCTy2-1 lie within the bracketed area labeled “2”. C) Mapping DSBs at the rightward moving fork. Unbroken, full length molecules are indicated at the position labeled “3”. Molecules with DSBs that arose origin distal to YDRCTy1-1 lie within the bracketed area labeled “4”. For (B) and (C), two independent trials using sister isolates are shown (MC2A = YJL8425 and YJL8426; MpGAL = YJL8427 and YJL8428). Breaks are evident in the re-replicating strains (MC2A) induced to re-replicate, and these increase in number with increased length of induction. These breaks depend upon re-replication, as they are not observed in the non-re-replicating control strains (MpGAL). The bands indicated with an * are unexplained major species which are not dependent upon re-replication. (PDF) [file pgen.1003192.s005.pdf]

Figure S6

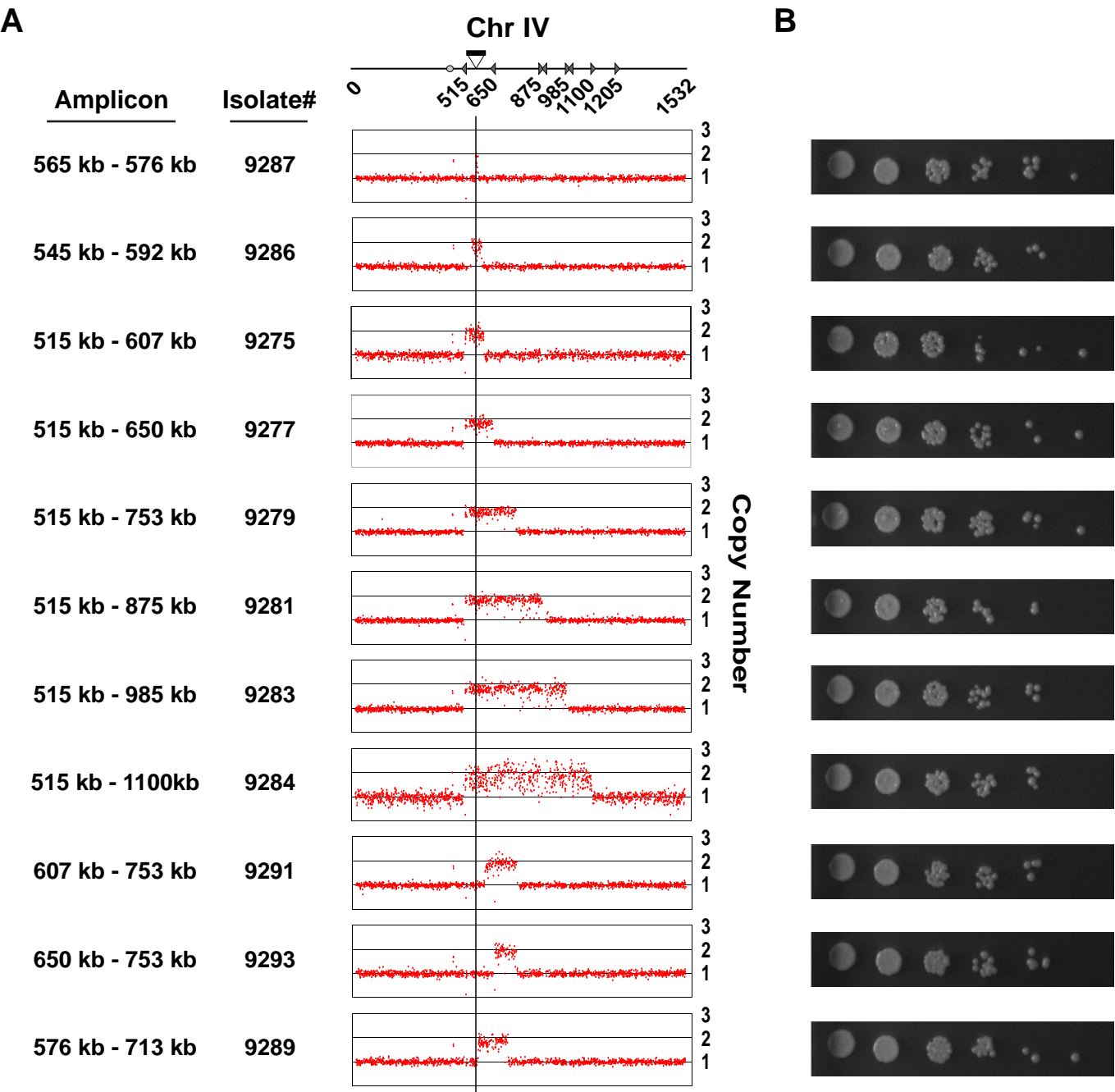

Supplement: Figure S6 — Decreased frequency of amplification observed for relocation of the amplicon boundaries is not caused by fitness defects. A) Uracil prototroph isolates for each combination of amplicon boundaries considered in Figure 5, Figure 6, and Figure S7 were analyzed using aCGH. Each isolate bears the expected amplification for the combination of amplicon boundaries present. B) Each isolate shown in panel (A) was tested for growth defects by a serial dilution spot test on SDC-Ura at 30°C (all on the same plate, 5-fold dilutions). All isolates grow with similar fitness. (PDF) [file pgen.1003192.s006.pdf]

Figure S7

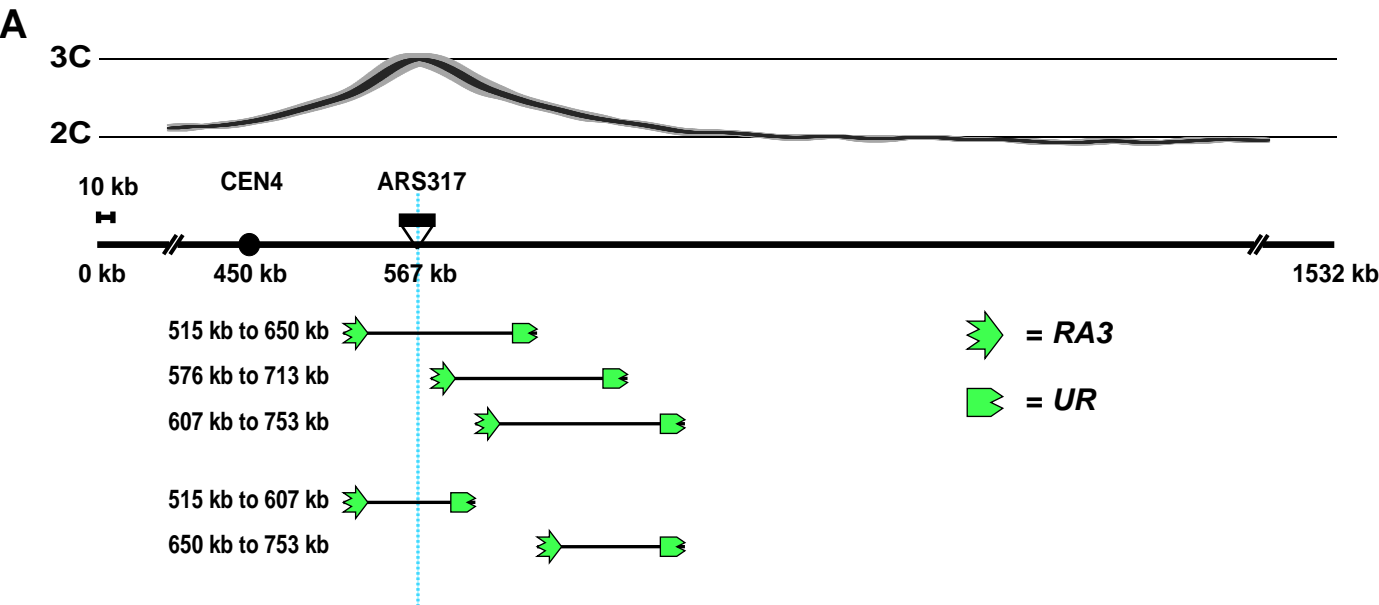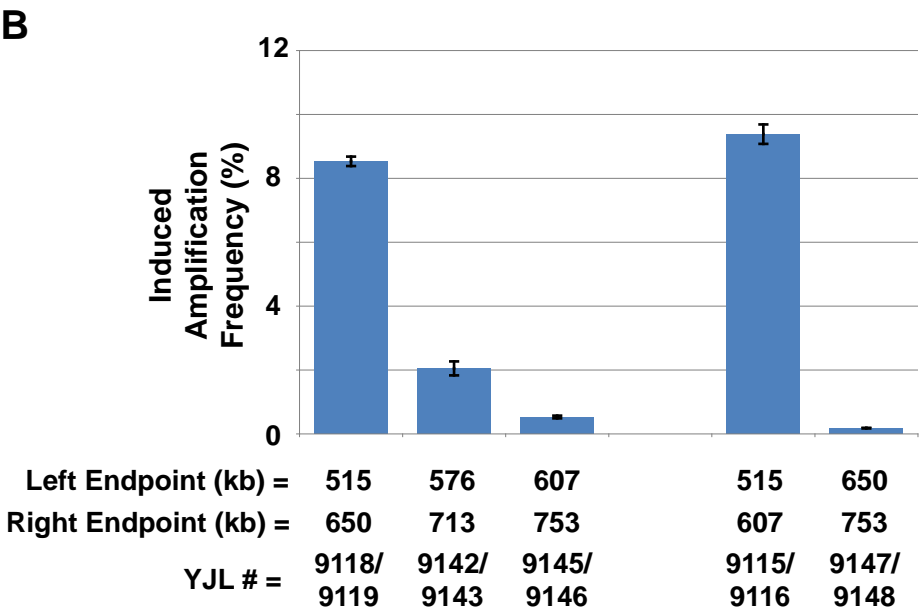

Supplement: Figure S7 — RRIGA proceeds most efficiently when the re-initiating origin lies within the amplicon. A) Schematic showing relocation of the RA3 and UR elements (3′ and 5′ portions of URA3, respectively) used in the URA3 RRIGA selection assay to change their position relative to the re-initiating origin (ARS317, light blue line) and the distribution of re-replication forks (as inferred from the re-replication profile). The upper group of amplicons are of a similar size, comparing a case where the origin is within the amplicon to a case with the origin just outside of the amplicon to a case with the origin at a great distance from the amplicon. The lower pair of amplicons are of a similar size (slightly smaller than the amplicons in the upper group), comparing a case where the origin is within the amplicon to a case with the origin at a very great distance from the amplicon. B) Induced amplification frequencies (mean ± SEM, n = 3) for strains with the indicated amplicon boundaries as defined by the position of the URA3 fragments. Induced frequency is frequency after 3 hr re-replication minus frequency after 0 hr re-replication. (PDF) [file pgen.1003192.s007.pdf]
