# Supplementary material for: Single-Stranded Annealing Induced by Re-Initiation of Replication Origins Provides a Novel and Efficient Mechanism for Generating Copy Number Expansion via Non-Allelic Homologous Recombination
Source: PLoS Genet. 2013 Jan 3;9(1):e1003192. doi: 10.1371/journal.pgen.1003192 (PMC3536649; doi:10.1371/journal.pgen.1003192)

Figure S3

# Isolates (from parent)					
Ty-Ty	*RA3-Ty*	*Ty-UR*	*RA3-UR*	Copy #	Boundaries (kb)
24	0	0	14	2	515 to 650
3	0	0	0	3	515 to 650
2	0	5	0	2	515 to 875
0	0	2	0	2	515 to Tel
0	0	1	0	2	435 to Tel
0	1	2	0	2	disomy
3	11	2	2	1	none

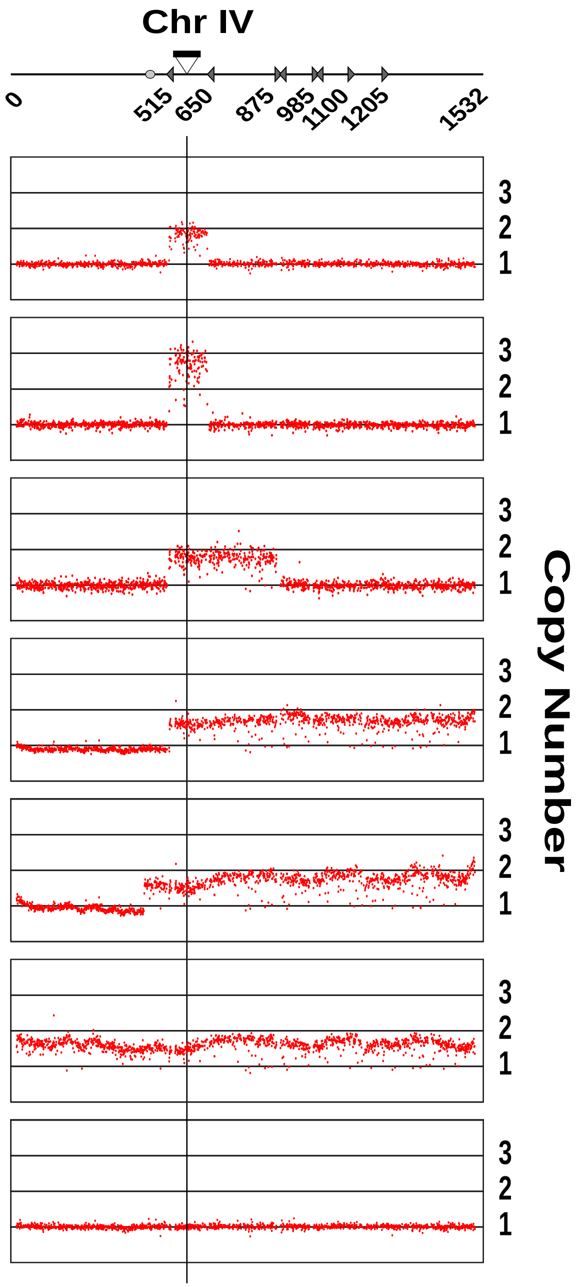

Supplement: Figure S3 — aCGH analysis of selected isolates from the sectoring assay. A subset of the post-induction (3 hr) isolates from the sectoring assay presented in Figure 1 were analyzed using aCGH. Representative aCGH profiles are shown with a tally of how frequently each profile was observed for each strain. Ty-Ty = YJL8100; RA3-Ty = YJL8355; Ty-UR = YJL8359; RA3-UR = YJL8363. Chromosome IV schematic shows positions of Ty elements (triangles, also showing orientation), centromere (circle), and ARS317-ade3-2p re-initiation cassette (bar and vertical line). (PDF) [file pgen.1003192.s003.pdf]
