# Supplementary material for: Single-Stranded Annealing Induced by Re-Initiation of Replication Origins Provides a Novel and Efficient Mechanism for Generating Copy Number Expansion via Non-Allelic Homologous Recombination
Source: PLoS Genet. 2013 Jan 3;9(1):e1003192. doi: 10.1371/journal.pgen.1003192 (PMC3536649; doi:10.1371/journal.pgen.1003192)
Supplement: Table S1 — Frequency of 1/2, 1/4, and 1/8 red sectored colonies observed in this work. (PDF) [file pgen.1003192.s008.pdf]

Table S1

Frequency of 1/2, 1/4, and 1/8 red sectored colonies observed in this work

Parent Strain	Genotype	Time	# Trials	Total Colonies Screened	Mean Sector Frequency	Standard Deviation	Standard Error of the Mean
YJL6974	*MCM7-2NLS pGAL YDRCTy2-1 YDRCTy1-1*	0 hr	2	2881	0.04%	0.05%	0.04%
YJL6974	*MCM7-2NLS pGAL YDRCTy2-1 YDRCTy1-1*	3 hr	2	7411	0.20%	0.01%	0.01%
YJL6558	*MCM7-2NLS pGAL-ΔntCDC6-cdk2A YDRCTy2-1 YDRCTy1-1*	0 hr	2	3731	0.05%	0.08%	0.05%
YJL6558	*MCM7-2NLS pGAL-ΔntCDC6-cdk2A YDRCTy2-1 YDRCTy1-1*	3 hr	2	3608	3.51%	0.20%	0.14%
YJL7445	*rad1Δ MCM7-2NLS pGAL-ΔntCDC6-cdk2A YDRCTy2-1 YDRCTy1-1*	0 hr	2	3648	0.08%	0.11%	0.08%
YJL7445	*rad1Δ MCM7-2NLS pGAL-ΔntCDC6-cdk2A YDRCTy2-1 YDRCTy1-1*	3 hr	2	5462	0.68%	0.13%	0.09%
YJL7451	*rad51Δ MCM7-2NLS pGAL-ΔntCDC6-cdk2A YDRCTy2-1 YDRCTy1-1*	0 hr	2	2888	0.30%	0.30%	0.21%
YJL7451	*rad51Δ MCM7-2NLS pGAL-ΔntCDC6-cdk2A YDRCTy2-1 YDRCTy1-1*	3 hr	2	5756	2.44%	0.62%	0.44%
YJL8100/ 8101	*MCM7-2NLS pGAL-ΔntCDC6-cdk2A YDRCTy2-1 YDRCTy1-1*	0 hr	5	8989	0.03%	0.04%	0.02%
YJL8100/ 8101	*MCM7-2NLS pGAL-ΔntCDC6-cdk2A YDRCTy2-1 YDRCTy1-1*	3 hr	5	7373	2.53%	0.48%	0.21%
YJL8104	*MCM7-2NLS pGAL-ΔntCDC6-cdk2A ydrcty2-1::RA3(v1) YDRCTy1-1*	0 hr	2	2976	0.08%	0.11%	0.08%

Table S1 (continued)

Frequency of 1/2, 1/4, and 1/8 red sectored colonies observed in this work

Parent Strain	Genotype	Time	# Trials	Total Colonies Screened	Mean Sector Frequency	Standard Deviation	Standard Error of the Mean
YJL8104	*MCM7-2NLS pGAL-ΔntCDC6-cdk2A ydrcty2-1::RA3(v1) YDRCTy1-1*	3 hr	2	6310	0.56%	0.18%	0.13%
YJL8108	*MCM7-2NLS pGAL-ΔntCDC6-cdk2A YDRCTy2-1 ydrcty1-1::UR(v1)*	0 hr	2	2834	0.05%	0.07%	0.05%
YJL8108	*MCM7-2NLS pGAL-ΔntCDC6-cdk2A YDRCTy2-1 ydrcty1-1::UR(v1)*	3 hr	2	7269	0.53%	0.24%	0.17%
YJL8112	*MCM7-2NLS pGAL-ΔntCDC6-cdk2A ydrcty2-1::RA3(v1) ydrcty1-1::UR(v1)*	0 hr	2	2961	0.14%	0.05%	0.04%
YJL8112	*MCM7-2NLS pGAL-ΔntCDC6-cdk2A ydrcty2-1::RA3(v1) ydrcty1-1::UR(v1)*	3 hr	2	2301	3.12%	0.15%	0.11%
YJL8355/ 8356	*MCM7-2NLS pGAL-ΔntCDC6-cdk2A ydrcty2-1::RA3(v2) YDRCTy1-1*	0 hr	3	6059	0.04%	0.03%	0.02%
YJL8355/ 8356	*MCM7-2NLS pGAL-ΔntCDC6-cdk2A ydrcty2-1::RA3(v2) YDRCTy1-1*	3 hr	3	12571	0.27%	0.09%	0.05%
YJL8359/ 8360	*MCM7-2NLS pGAL-ΔntCDC6-cdk2A YDRCTy2-1 ydrcty1-1::UR(v2)*	0 hr	3	5887	0.03%	0.05%	0.03%
YJL8359/ 8360	*MCM7-2NLS pGAL-ΔntCDC6-cdk2A YDRCTy2-1 ydrcty1-1::UR(v2)*	3 hr	3	13076	0.55%	0.18%	0.10%
YJL8363/ 8364	*MCM7-2NLS pGAL-ΔntCDC6-cdk2A ydrcty2-1::RA3(v2) ydrcty1-1::UR(v2)*	0 hr	4	7948	0.04%	0.03%	0.01%
YJL8363/ 8364	*MCM7-2NLS pGAL-ΔntCDC6-cdk2A ydrcty2-1::RA3(v2) ydrcty1-1::UR(v2)*	3 hr	4	6298	3.13%	0.38%	0.19%
