# Supplementary material for: Single-Stranded Annealing Induced by Re-Initiation of Replication Origins Provides a Novel and Efficient Mechanism for Generating Copy Number Expansion via Non-Allelic Homologous Recombination
Source: PLoS Genet. 2013 Jan 3;9(1):e1003192. doi: 10.1371/journal.pgen.1003192 (PMC3536649; doi:10.1371/journal.pgen.1003192)
Supplement: Table S2 — aCGH analysis of red-sectored colony isolates. (PDF) [file pgen.1003192.s009.pdf]

Table S2

aCGH analysis of red-sectored colony isolates.

Parent Strain	Isolate	Timepont	Copy Number	Amplicon Boundaries (kb)	Other CGH Changes
YJL6558	YJL7860	0 hr	2	515 to 650	none
YJL6558	YJL7782	3 hr	2	515 to 650	none
YJL6558	YJL7783	3 hr	2	515 to 875	none
YJL6558	YJL7784	3 hr	2	515 to 650	none
YJL6558	YJL7785	3 hr	2	515 to 650	none
YJL6558	YJL7786	3 hr	2	515 to 650	none
YJL6558	YJL7787	3 hr	2	515 to 650	ChrIII Disomy
YJL6558	YJL7861	3 hr	2	515 to 650	none
YJL6558	YJL7862	3 hr	2	515 to 650	none
YJL6558	YJL7863	3 hr	2	515 to 650	none
YJL6558	YJL7864	3 hr	2	515 to 650	none
YJL6974	YJL7788	3 hr	1	n/a	ChrIV Disomy*
YJL6974	YJL7789	3 hr	1	n/a	none
YJL6974	YJL7900	3 hr	2	515 to 875	none*
YJL6974	YJL7901	3 hr	1	n/a	none
YJL7445	YJL7865	0 hr	1	n/a	ChrV Disomy*
YJL7445	YJL7866	0 hr	1	n/a	none
YJL7445	YJL7867	0 hr	1	n/a	none
YJL7445	YJL7790	3 hr	2	515 to 650	none
YJL7445	YJL7791	3 hr	1	n/a	ChrV Disomy
YJL7445	YJL7792	3 hr	1	n/a	none
YJL7445	YJL7793	3 hr	2	515 to 650	none
YJL7445	YJL7794	3 hr	1	n/a	none
YJL7445	YJL7795	3 hr	2	515 to 650	none
YJL7445	YJL7796	3 hr	2	515 to 650	none
YJL7445	YJL7797	3 hr	1	n/a	Segmental Duplication of ChrIII_TEL-167kb; Segmental Duplication of ChrXII_688kb-TEL

Table S2 (continued)

YJL7445	YJL7798	3 hr	1	n/a	ChrV Disomy
YJL7445	YJL7799	3 hr	2	515 to 650	Segmental Duplication of ChrIII_150-167kb
YJL7445	YJL7800	3 hr	2	515 to 650	none
YJL7445	YJL7801	3 hr	1	n/a	none
YJL7445	YJL7802	3 hr	1	n/a	ChrV Disomy
YJL7445	YJL7803	3 hr	1	n/a	ChrV Disomy
YJL7445	YJL7804	3 hr	2	515 to 650	none
YJL7445	YJL7805	3 hr	2	515 to 650	none
YJL7445	YJL7806	3 hr	2	515 to 650	none
YJL7445	YJL7807	3 hr	2	515 to 650	none
YJL7445	YJL7808	3 hr	2	515 to 650	none
YJL7445	YJL7809	3 hr	2	515 to 650	none
YJL7445	YJL7868	3 hr	2	435 to 650	none*
YJL7445	YJL7869	3 hr	2	515 to 650	none
YJL7445	YJL7870	3 hr	1	n/a	none
YJL7445	YJL7871	3 hr	2	515 to 650	none
YJL7445	YJL7872	3 hr	1	n/a	ChrV Disomy
YJL7445	YJL7873	3 hr	1	n/a	none
YJL7445	YJL7874	3 hr	2	515 to 650	none
YJL7445	YJL7875	3 hr	1	n/a	ChrV Disomy
YJL7451	YJL7876	0 hr	1	n/a	none
YJL7451	YJL7877	0 hr	1	n/a	none
YJL7451	YJL7878	0 hr	1	n/a	none
YJL7451	YJL7879	0 hr	1	n/a	none
YJL7451	YJL7880	0 hr	1	n/a	none
YJL7451	YJL7881	0 hr	1	n/a	none
YJL7451	YJL7882	0 hr	1	n/a	none

Table S2 (continued)

YJL7451	YJL7883	0 hr	1	n/a	none
YJL7451	YJL7810	3 hr	2	515 to 650	ChrIII Disomy
YJL7451	YJL7811	3 hr	2	515 to 650	none
YJL7451	YJL7812	3 hr	2	515 to 650	none
YJL7451	YJL7813	3 hr	2	515 to 650	none
YJL7451	YJL7814	3 hr	2	515 to 650	none
YJL7451	YJL7815	3 hr	1	n/a	none
YJL7451	YJL7816	3 hr	2	515 to 650	none
YJL7451	YJL7817	3 hr	2	515 to 650	none
YJL7451	YJL7818	3 hr	2	515 to 650	none
YJL7451	YJL7819	3 hr	1	n/a	none
YJL7451	YJL7820	3 hr	2	515 to 650	none
YJL7451	YJL7821	3 hr	2	515 to 650	none
YJL7451	YJL7822	3 hr	1	n/a	ChrV Disomy
YJL7451	YJL7823	3 hr	2	515 to 875	none
YJL7451	YJL7824	3 hr	2	515 to 650	none
YJL7451	YJL7825	3 hr	2	515 to 875	none
YJL7451	YJL7826	3 hr	2	515 to 650	none
YJL7451	YJL7827	3 hr	2	515 to 650	none
YJL7451	YJL7828	3 hr	1	n/a	none
YJL7451	YJL7829	3 hr	2	515 to 650	none
YJL7451	YJL7884	3 hr	2	515 to 650	none
YJL7451	YJL7885	3 hr	2	515 to 650	none
YJL7451	YJL7886	3 hr	1	n/a	none
YJL7451	YJL7887	3 hr	2	515 to 650	none
YJL7451	YJL7888	3 hr	1	n/a	none
YJL7451	YJL7889	3 hr	2	515 to 875	none*

Table S2 (continued)

YJL7451	YJL7890	3 hr	2	515 to 650	none
YJL7451	YJL7891	3 hr	2	515 to 650	none
YJL7451	YJL7892	3 hr	2	515 to 650	none*
YJL7451	YJL7893	3 hr	2	515 to 650	none
YJL7451	YJL7894	3 hr	2	515 to 650	none
YJL7451	YJL7895	3 hr	2	515 to 650	Segmental Duplication of ChrIII_150-167kb*
YJL7451	YJL7896	3 hr	2	515 to 650	none
YJL7451	YJL7897	3 hr	2	515 to 875	none
YJL7451	YJL7898	3 hr	2	515 to 650	none
YJL7451	YJL7899	3 hr	2	515 to 650	none
YJL8100	YJL8283	0 hr	1	n/a	ChrV Disomy
YJL8100	YJL9482	0 hr	1	n/a	ChrV Disomy
YJL8100	YJL8128	3 hr	3	515 to 650	none
YJL8100	YJL8129	3 hr	3	515 to 650	none
YJL8100	YJL8130	3 hr	2	515 to 650	none
YJL8100	YJL8131	3 hr	2	515 to 650	none
YJL8100	YJL8132	3 hr	1	n/a	Segmental Duplication of ChrV_289-443kb
YJL8100	YJL8133	3 hr	2	515 to 650	none
YJL8100	YJL8134	3 hr	2	515 to 650	none
YJL8100	YJL8135	3 hr	2	515 to 875	none
YJL8100	YJL8136	3 hr	2	515 to 650	none
YJL8100	YJL8137	3 hr	3	515 to 650	none
YJL8100	YJL8138	3 hr	2	515 to 650	none
YJL8100	YJL8139	3 hr	2	515 to 650	none
YJL8100	YJL8287	3 hr	2	515 to 650	none
YJL8100	YJL8288	3 hr	2	515 to 650	none
YJL8100	YJL8289	3 hr	2	515 to 650	none*

Table S2 (continued)

YJL8100	YJL8290	3 hr	1	n/a	none
YJL8100	YJL8291	3 hr	3	515 to 650	none
YJL8100	YJL8292	3 hr	2	515 to 650	none
YJL8100	YJL8293	3 hr	2	515 to 650	none
YJL8100	YJL8294	3 hr	2	515 to 650	none
YJL8100	YJL9483	3 hr	2	515 to 650	none
YJL8100	YJL9484	3 hr	2	515 to 650	none
YJL8100	YJL9485	3 hr	2	515 to 650	none
YJL8100	YJL9486	3 hr	2	515 to 650	none
YJL8100	YJL9487	3 hr	2	515 to 650	none
YJL8100	YJL9488	3 hr	2	515 to 650	none
YJL8100	YJL9489	3 hr	2	515 to 650	none
YJL8100	YJL9490	3 hr	1	n/a	ChrXIII Disomy
YJL8100	YJL9491	3 hr	2	515 to 875	none
YJL8100	YJL9492	3 hr	2	515 to 650	none
YJL8100	YJL9493	3 hr	2	515 to 650	none
YJL8100	YJL9494	3 hr	2	515 to 650	none
YJL8104	YJL8140	0 hr	1	n/a	ChrXIII Disomy
YJL8104	YJL8141	0 hr	1	n/a	ChrII Disomy
YJL8104	YJL8142	0 hr	1	n/a	ChrII Disomy
YJL8104	YJL8143	3 hr	1	n/a	ChrXIII Disomy
YJL8104	YJL8144	3 hr	1	n/a	ChrIV Disomy
YJL8104	YJL8145	3 hr	2	515 to 875	none
YJL8104	YJL8146	3 hr	1	n/a	Segmental Duplication of ChrXIII_379-838kb
YJL8104	YJL8147	3 hr	1	n/a	ChrV Disomy
YJL8104	YJL8148	3 hr	2	435 to 650	none
YJL8104	YJL8149	3 hr	2	515 to 650	none

Table S2 (continued)

YJL8104	YJL8150	3 hr	2	515 to 875	none
YJL8104	YJL8151	3 hr	1	n/a	ChrXIII Disomy
YJL8104	YJL8152	3 hr	2	515 to 875	none
YJL8104	YJL8153	3 hr	2	567 to 875	none
YJL8104	YJL8154	3 hr	2	515 to 875	none
YJL8104	YJL8155	3 hr	2	515 to 805	none
YJL8104	YJL8156	3 hr	2	515 to 805	none
YJL8104	YJL8295	3 hr	1	n/a	none
YJL8104	YJL8296	3 hr	1	n/a	ChrXIII Disomy
YJL8104	YJL8297	3 hr	3	515 to 805	none
YJL8104	YJL8298	3 hr	1	n/a	ChrI Monosomy*
YJL8104	YJL8299	3 hr	1	n/a	ChrXIII Disomy*
YJL8104	YJL8300	3 hr	1	n/a	Segmental Duplication of ChrIV_650-875kb; Segmental Duplication of ChrXII_688-980kb
YJL8104	YJL8301	3 hr	1	n/a	none
YJL8104	YJL8302	3 hr	2	515 to TEL	Segmental Duplication of ChrX_TEL-537kb
YJL8104	YJL8303	3 hr	2	515 to 805	none
YJL8104	YJL8304	3 hr	1	n/a	ChrV Disomy
YJL8104	YJL8305	3 hr	1	n/a	ChrIII Disomy*
YJL8104	YJL8306	3 hr	1	n/a	Segmental Duplication of ChrII_TEL-260kb; Segmental Duplication of ChrXII_688kb-TEL
YJL8104	YJL8307	3 hr	1	n/a	ChrXIII Disomy
YJL8104	YJL8308	3 hr	3	515 to 985	none
YJL8108	YJL8284	0 hr	1	n/a	none
YJL8108	YJL8157	3 hr	1	n/a	ChrXIII Disomy
YJL8108	YJL8158	3 hr	1	n/a	none
YJL8108	YJL8159	3 hr	1	n/a	ChrXIII Disomy
YJL8108	YJL8160	3 hr	1	n/a	none
YJL8108	YJL8161	3 hr	2	515 to 805	none

Table S2 (continued)

YJL8108	YJL8162	3 hr	1	n/a	Segmental Duplication of ChrXIII_379-838kb
YJL8108	YJL8163	3 hr	2	515 to TEL	Segmental Duplication of ChrVI_144kb-TEL
YJL8108	YJL8164	3 hr	2	515 to 875	none
YJL8108	YJL8165	3 hr	2	515 to 805	none
YJL8108	YJL8166	3 hr	2	515 to 805	none
YJL8108	YJL8167	3 hr	3	515 to 650	none
YJL8108	YJL8168	3 hr	1	n/a	Segmental Duplication of ChrXII_TEL-221kb; Segmental Duplication of ChrXIII_379kb-TEL
YJL8108	YJL8169	3 hr	1	n/a	none
YJL8108	YJL8170	3 hr	2	515 to 875	none
YJL8108	YJL8309	3 hr	2	515 to 875	none
YJL8108	YJL8310	3 hr	2	515 to 805	none
YJL8108	YJL8311	3 hr	1	n/a	ChrIII Trisomy; ChrXVI Disomy
YJL8108	YJL8312	3 hr	2	515 to 875	none
YJL8108	YJL8313	3 hr	1	n/a	ChrI Monosomy*
YJL8108	YJL8314	3 hr	2	515 to 805	none
YJL8108	YJL8315	3 hr	2	515 to 805	none
YJL8108	YJL8316	3 hr	1	n/a	none
YJL8108	YJL8317	3 hr	1	n/a	ChrV Disomy
YJL8108	YJL8318	3 hr	2	515 to 875	none
YJL8108	YJL8319	3 hr	2	515 to 875	none
YJL8108	YJL8320	3 hr	2	515 to 875	none
YJL8108	YJL8321	3 hr	2	515 to 875	none
YJL8108	YJL8322	3 hr	2	515 to 805	none
YJL8112	YJL8171	0 hr	1	n/a	ChrII Disomy
YJL8112	YJL8172	0 hr	1	n/a	ChrIII Disomy
YJL8112	YJL8285	0 hr	1	n/a	ChrII Disomy
YJL8112	YJL8286	0 hr	1	n/a	ChrII Disomy

Table S2 (continued)

YJL8112	YJL8173	3 hr	2	515 to 650	none
YJL8112	YJL8174	3 hr	2	515 to 650	none
YJL8112	YJL8175	3 hr	2	515 to 650	none
YJL8112	YJL8176	3 hr	2	515 to 650	none
YJL8112	YJL8177	3 hr	2	515 to 650	none
YJL8112	YJL8178	3 hr	2	515 to 650	none
YJL8112	YJL8179	3 hr	2	515 to 650	none
YJL8112	YJL8180	3 hr	2	515 to 650	none
YJL8112	YJL8181	3 hr	2	515 to 650	none
YJL8112	YJL8182	3 hr	1	n/a	Segmental Duplication of ChrXIII_769-838kb
YJL8112	YJL8183	3 hr	1	n/a	none
YJL8112	YJL8184	3 hr	2	515 to 650	none
YJL8112	YJL8323	3 hr	2	515 to 650	none
YJL8112	YJL8324	3 hr	2	515 to 650	none
YJL8112	YJL8325	3 hr	1	n/a	none
YJL8112	YJL8326	3 hr	2	515 to 650	none
YJL8112	YJL8327	3 hr	2	515 to 650	none
YJL8112	YJL8328	3 hr	2	515 to 650	none
YJL8112	YJL8329	3 hr	3	515 to 650	none
YJL8112	YJL8330	3 hr	2	515 to 650	none
YJL8355	YJL9495	0 hr	1	n/a	ChrII Disomy
YJL8355	YJL9496	0 hr	1	n/a	none
YJL8355	YJL9497	3 hr	1	n/a	ChrXIV Monosomy*
YJL8355	YJL9498	3 hr	1	n/a	none
YJL8355	YJL9499	3 hr	1	n/a	none
YJL8355	YJL9500	3 hr	1	n/a	ChrV Disomy
YJL8355	YJL9501	3 hr	1	n/a	ChrIII Disomy

Table S2 (continued)

YJL8355	YJL9502	3 hr	1	n/a	Segmental Duplication of ChrIII_TEL-75kb; Segmental Duplication of ChrIX_325kb-TEL
YJL8355	YJL9503	3 hr	1	n/a	none
YJL8355	YJL9504	3 hr	1	n/a	ChrIII Disomy
YJL8355	YJL9505	3 hr	1	n/a	ChrV Disomy
YJL8355	YJL9506	3 hr	1	n/a	none
YJL8355	YJL9507	3 hr	1	n/a	ChrIV Disomy
YJL8355	YJL9508	3 hr	1	n/a	Segmental Triplication of ChrXII_534-656kb; Segmental Duplication of ChrXII_656-688kb
YJL8359	YJL9509	0 hr	1	n/a	none
YJL8359	YJL9518	0 hr	1	n/a	ChrV Disomy
YJL8359	YJL9519	0 hr	1	n/a	none
YJL8359	YJL9510	3 hr	3	515 to 875	none
YJL8359	YJL9511	3 hr	1	n/a	ChrV Disomy
YJL8359	YJL9512	3 hr	2	515 to TEL	Segmental Duplication of ChrIII_TEL-150kb
YJL8359	YJL9513	3 hr	2	515 to 875	none
YJL8359	YJL9514	3 hr	2	515 to 875	none
YJL8359	YJL9515	3 hr	2	515 to 875	none
YJL8359	YJL9516	3 hr	1	n/a	ChrII Disomy
YJL8359	YJL9517	3 hr	2	515 to 875	none
YJL8359	YJL9520	3 hr	2	515 to TEL	Segmental Duplication of ChrX_TEL-540kb
YJL8359	YJL9521	3 hr	1	n/a	ChrIV Disomy
YJL8359	YJL9522	3 hr	1	n/a	ChrIV Disomy
YJL8359	YJL9523	3 hr	2	435 to TEL	Segmental Duplication of ChrVII_TEL-110kb
YJL8363	YJL9524	0 hr	1	n/a	ChrXIII Disomy
YJL8363	YJL9525	0 hr	1	n/a	ChrIV Disomy
YJL8363	YJL9538	0 hr	2	515 to 650	none
YJL8363	YJL9526	3 hr	2	515 to 650	none
YJL8363	YJL9527	3 hr	2	515 to 650	none

Table S2 (continued)

YJL8363	YJL9528	3 hr	2	515 to 650	none
YJL8363	YJL9529	3 hr	2	515 to 650	none
YJL8363	YJL9530	3 hr	2	515 to 650	none
YJL8363	YJL9531	3 hr	2	515 to 650	none
YJL8363	YJL9532	3 hr	2	515 to 650	Chr II Disomy
YJL8363	YJL9533	3 hr	1	n/a	none
YJL8363	YJL9534	3 hr	1	n/a	ChrII Disomy
YJL8363	YJL9535	3 hr	2	515 to 650	none
YJL8363	YJL9536	3 hr	2	515 to 650	none
YJL8363	YJL9537	3 hr	2	515 to 650	none
YJL8363	YJL9539	3 hr	2	515 to 650	none
YJL8363	YJL9540	3 hr	2	515 to 650	none
YJL8363	YJL9541	3 hr	2	515 to 650	none
YJL8363	YJL9542	3 hr	2	515 to 650	none

* - aCGH suggests possible spontaneous diploid or mixed population. This is indicated either by the points in amplified region scattering at a non-quantile value (ie. 1.5) or by the points of an aneuploid chromosome scattering at a non-quantile value. aCGH will not be able to suggest possibly diploidization in cases of amplicons with quantile values or where there are no aneuploidies.
