# Supplementary material for: Single-Stranded Annealing Induced by Re-Initiation of Replication Origins Provides a Novel and Efficient Mechanism for Generating Copy Number Expansion via Non-Allelic Homologous Recombination
Source: PLoS Genet. 2013 Jan 3;9(1):e1003192. doi: 10.1371/journal.pgen.1003192 (PMC3536649; doi:10.1371/journal.pgen.1003192)
Supplement: Table S3 — Frequency of uracil prototrophs from the selection assay observed in this work. (PDF) [file pgen.1003192.s010.pdf]

Table S3

Frequency of uracil prototrophs from the selection assay observed in this work.

Parent Strain	Genotype	Time	# Trials	Total cfu Tested	Mean Ura$^+$ Frequency	Standard Deviation	Standard Error of the Mean
YJL8112/ 8113	*MCM7-2NLS pGAL-ΔntCDC6-cdk2A ydrcty2-1::RA3(v1) ydrcty1-1::UR(v1)*	0 hr	2	53750	0.34%	0.08%	0.06%
YJL8112/ 8113	*MCM7-2NLS pGAL-ΔntCDC6-cdk2A ydrcty2-1::RA3(v1) ydrcty1-1::UR(v1)*	3 hr	2	35500	6.85%	0.23%	0.17%
YJL8363/ 8364	*MCM7-2NLS pGAL-ΔntCDC6-cdk2A ydrcty2-1::RA3(v2) ydrcty1-1::UR(v2)*	0 hr	5	109850	0.32%	0.05%	0.02%
YJL8363/ 8364	*MCM7-2NLS pGAL-ΔntCDC6-cdk2A ydrcty2-1::RA3(v2) ydrcty1-1::UR(v2)*	3 hr	5	91000	10.12%	2.21%	0.99%
YJL9149- 9151	*MCM7-2NLS pGAL ydrcty2-1::RA3(v2) ydrcty1-1::UR(v2)*	0 hr	3	48150	0.22%	0.03%	0.02%
YJL9149- 9151	*MCM7-2NLS pGAL ydrcty2-1::RA3(v2) ydrcty1-1::UR(v2)*	3 hr	3	64950	0.20%	0.04%	0.02%
YJL8407/ 8408	*dnl4Δ MCM7-2NLS pGAL-ΔntCDC6-cdk2A ydrcty2-1::RA3(v2) ydrcty1-1::UR(v2)*	0 hr	2	42650	0.17%	0.01%	0.01%
YJL8407/ 8408	*dnl4Δ MCM7-2NLS pGAL-ΔntCDC6-cdk2A ydrcty2-1::RA3(v2) ydrcty1-1::UR(v2)*	3 hr	2	25800	10.13%	1.16%	0.82%
YJL8409/ 8410	*rad52Δ MCM7-2NLS pGAL-ΔntCDC6-cdk2A ydrcty2-1::RA3(v2) ydrcty1-1::UR(v2)*	0 hr	2	34850	0.00%	0.00%	0.00%
YJL8409/ 8410	*rad52Δ MCM7-2NLS pGAL-ΔntCDC6-cdk2A ydrcty2-1::RA3(v2) ydrcty1-1::UR(v2)*	3 hr	2	12050	0.04%	0.02%	0.02%

Table S3 (continued)

Frequency of uracil prototrophs from the selection assay observed in this work

Parent Strain	Genotype	Time	# Trials	Total cfu Tested	Mean Ura+ Frequency	Standard Deviation	Standard Error of the Mean
YJL8412/ 8413	*rad51Δ MCM7-2NLS pGAL-ΔntCDC6-cdk2A ydrcty2-1::RA3(v2) ydrcty1-1::UR(v2)*	0 hr	2	35150	0.15%	0.05%	0.04%
YJL8412/ 8413	*rad51Δ MCM7-2NLS pGAL-ΔntCDC6-cdk2A ydrcty2-1::RA3(v2) ydrcty1-1::UR(v2)*	3 hr	2	25000	8.42%	0.15%	0.11%
YJL8415/ 8416	*rad1Δ MCM7-2NLS pGAL-ΔntCDC6-cdk2A ydrcty2-1::RA3(v2) ydrcty1-1::UR(v2)*	0 hr	2	29100	0.00%	0.00%	0.00%
YJL8415/ 8416	*rad1Δ MCM7-2NLS pGAL-ΔntCDC6-cdk2A ydrcty2-1::RA3(v2) ydrcty1-1::UR(v2)*	3 hr	2	11550	0.41%	0.03%	0.02%
YJL8418/ 8419	*msh3Δ MCM7-2NLS pGAL-ΔntCDC6-cdk2A ydrcty2-1::RA3(v2) ydrcty1-1::UR(v2)*	0 hr	2	30900	0.01%	0.00%	0.00%
YJL8418/ 8419	*msh3Δ MCM7-2NLS pGAL-ΔntCDC6-cdk2A ydrcty2-1::RA3(v2) ydrcty1-1::UR(v2)*	3 hr	2	12450	1.19%	0.16%	0.11%
YJL8421/ 8422	*pol32Δ MCM7-2NLS pGAL-ΔntCDC6-cdk2A ydrcty2-1::RA3(v2) ydrcty1-1::UR(v2)*	0 hr	2	20100	0.04%	0.01%	0.01%
YJL8421/ 8422	*pol32Δ MCM7-2NLS pGAL-ΔntCDC6-cdk2A ydrcty2-1::RA3(v2) ydrcty1-1::UR(v2)*	3 hr	2	8850	4.82%	0.03%	0.02%
YJL9115/ 9116	*MCM7-2NLS pGAL-ΔntCDC6-cdk2A ydrcty2-1Δ ydrcty1-1Δ ChrIV_515kb::RA3 ChrIV_607kb::UR*	0 hr	3	46950	0.48%	0.10%	0.06%
YJL9115/ 9116	*MCM7-2NLS pGAL-ΔntCDC6-cdk2A ydrcty2-1Δ ydrcty1-1Δ ChrIV_515kb::RA3 ChrIV_607kb::UR*	3 hr	3	42750	9.86%	0.51%	0.29%

Table S3 (continued)

Frequency of uracil prototrophs from the selection assay observed in this work

Parent Strain	Genotype	Time	# Trials	Total cfu Tested	Mean Ura$^+$ Frequency	Standard Deviation	Standard Error of the Mean
YJL9118/ 9119	*MCM7-2NLS pGAL-ΔntCDC6-cdk2A ydrcty2-1Δ ydrcty1-1Δ ChrIV_515kb::RA3 ChrIV_650kb::UR*	0 hr	3	49500	0.25%	0.01%	0.00%
YJL9118/ 9119	*MCM7-2NLS pGAL-ΔntCDC6-cdk2A ydrcty2-1Δ ydrcty1-1Δ ChrIV_515kb::RA3 ChrIV_650kb::UR*	3 hr	3	45300	8.78%	0.26%	0.15%
YJL9121/ 9122	*MCM7-2NLS pGAL-ΔntCDC6-cdk2A ydrcty2-1Δ ydrcty1-1Δ ChrIV_515kb::RA3 ChrIV_753kb::UR*	0 hr	3	49350	0.12%	0.01%	0.01%
YJL9121/ 9122	*MCM7-2NLS pGAL-ΔntCDC6-cdk2A ydrcty2-1Δ ydrcty1-1Δ ChrIV_515kb::RA3 ChrIV_753kb::UR*	3 hr	3	42450	3.06%	0.55%	0.32%
YJL9127/ 9128	*MCM7-2NLS pGAL-ΔntCDC6-cdk2A ydrcty2-1Δ ydrcty1-1Δ ChrIV_515kb::RA3 ChrIV_875kb::UR*	0 hr	3	50100	0.05%	0.02%	0.01%
YJL9127/ 9128	*MCM7-2NLS pGAL-ΔntCDC6-cdk2A ydrcty2-1Δ ydrcty1-1Δ ChrIV_515kb::RA3 ChrIV_875kb::UR*	3 hr	3	43200	1.15%	0.12%	0.07%
YJL9130/ 9131	*MCM7-2NLS pGAL-ΔntCDC6-cdk2A ydrcty2-1Δ ydrcty1-1Δ ChrIV_515kb::RA3 ChrIV_985kb::UR*	0 hr	3	50550	0.01%	0.01%	0.01%
YJL9130/ 9131	*MCM7-2NLS pGAL-ΔntCDC6-cdk2A ydrcty2-1Δ ydrcty1-1Δ ChrIV_515kb::RA3 ChrIV_985kb::UR*	3 hr	3	38400	0.15%	0.05%	0.03%
YJL9133/ 9134	*MCM7-2NLS pGAL-ΔntCDC6-cdk2A ydrcty2-1Δ ydrcty1-1Δ ChrIV_515kb::RA3 ChrIV_1100kb::UR*	0 hr	3	49500	0.00%	0.01%	0.00%
YJL9133/ 9134	*MCM7-2NLS pGAL-ΔntCDC6-cdk2A ydrcty2-1Δ ydrcty1-1Δ ChrIV_515kb::RA3 ChrIV_1100kb::UR*	3 hr	3	40650	0.08%	0.03%	0.01%

Table S3 (continued)

Frequency of uracil prototrophs from the selection assay observed in this work

Parent Strain	Genotype	Time	# Trials	Total cfu Tested	Mean Ura$^+$ Frequency	Standard Deviation	Standard Error of the Mean
YJL9136/ 9137	*MCM7-2NLS pGAL-ΔntCDC6-cdk2A ydrcty2-1Δ ydrcty1-1Δ ChrIV_545kb::RA3 ChrIV_592kb::UR*	0 hr	3	46950	0.79%	0.22%	0.12%
YJL9136/ 9137	*MCM7-2NLS pGAL-ΔntCDC6-cdk2A ydrcty2-1Δ ydrcty1-1Δ ChrIV_545kb::RA3 ChrIV_592kb::UR*	3 hr	3	44100	13.29%	0.40%	0.23%
YJL9139/ 9140	*MCM7-2NLS pGAL-ΔntCDC6-cdk2A ydrcty2-1Δ ydrcty1-1Δ ChrIV_565kb::RA3 ChrIV_576kb::UR*	0 hr	3	46800	0.44%	0.10%	0.06%
YJL9139/ 9140	*MCM7-2NLS pGAL-ΔntCDC6-cdk2A ydrcty2-1Δ ydrcty1-1Δ ChrIV_565kb::RA3 ChrIV_576kb::UR*	3 hr	3	43500	11.47%	1.32%	0.76%
YJL9142/ 9143	*MCM7-2NLS pGAL-ΔntCDC6-cdk2A ydrcty2-1Δ ydrcty1-1Δ ChrIV_576kb::RA3 ChrIV_713kb::UR*	0 hr	3	50850	0.04%	0.01%	0.00%
YJL9142/ 9143	*MCM7-2NLS pGAL-ΔntCDC6-cdk2A ydrcty2-1Δ ydrcty1-1Δ ChrIV_576kb::RA3 ChrIV_713kb::UR*	3 hr	3	38850	2.09%	0.37%	0.22%
YJL9145/ 9146	*MCM7-2NLS pGAL-ΔntCDC6-cdk2A ydrcty2-1Δ ydrcty1-1Δ ChrIV_607kb::RA3 ChrIV_753kb::UR*	0 hr	3	50100	0.02%	0.01%	0.01%
YJL9145/ 9146	*MCM7-2NLS pGAL-ΔntCDC6-cdk2A ydrcty2-1Δ ydrcty1-1Δ ChrIV_607kb::RA3 ChrIV_753kb::UR*	3 hr	3	42300	0.55%	0.08%	0.05%
YJL9147/ 9148	*MCM7-2NLS pGAL-ΔntCDC6-cdk2A ydrcty2-1Δ ydrcty1-1Δ ChrIV_650kb::RA3 ChrIV_753kb::UR*	0 hr	3	46050	0.05%	0.04%	0.02%
YJL9147/ 9148	*MCM7-2NLS pGAL-ΔntCDC6-cdk2A ydrcty2-1Δ ydrcty1-1Δ ChrIV_650kb::RA3 ChrIV_753kb::UR*	3 hr	3	41550	0.23%	0.04%	0.02%
