# Supplementary material for: Single-Stranded Annealing Induced by Re-Initiation of Replication Origins Provides a Novel and Efficient Mechanism for Generating Copy Number Expansion via Non-Allelic Homologous Recombination
Source: PLoS Genet. 2013 Jan 3;9(1):e1003192. doi: 10.1371/journal.pgen.1003192 (PMC3536649; doi:10.1371/journal.pgen.1003192)
Supplement: Table S4 — aCGH analysis of uracil prototroph isolates. (PDF) [file pgen.1003192.s011.pdf]

Table S4

aCGH analysis of uracil prototroph isolates.

Parent Strain	Isolate	Timepont	Copy Number	Amplicon Boundaries (kb)	Other CGH Changes
YJL8112	YJL8431	0 hr	2	515 to 650	none
YJL8112	YJL8432	0 hr	2	515 to 650	ChrV Disomy*
YJL8112	YJL8433	0 hr	2	515 to 650	none
YJL8113	YJL8434	0 hr	2	515 to 650	none
YJL8113	YJL8435	0 hr	3	515 to 650	none
YJL8113	YJL8436	0 hr	2	515 to 650	ChrII Disomy; ChrVII Disomy
YJL8363	YJL8437	0 hr	2	515 to 650	none
YJL8363	YJL8438	0 hr	2	515 to 650	none
YJL8363	YJL8439	0 hr	2	515 to 650	none
YJL8364	YJL8440	0 hr	2	515 to 650	none
YJL8364	YJL8441	0 hr	2	515 to 650	none
YJL8364	YJL8442	0 hr	2	515 to 650	none
YJL8112	YJL8443	3 hr	2	515 to 650	none
YJL8112	YJL8444	3 hr	2	515 to 650	none
YJL8112	YJL8445	3 hr	2	515 to 650	ChrX Disomy
YJL8112	YJL8446	3 hr	3	515 to 650	none
YJL8112	YJL8447	3 hr	2	515 to 650	none
YJL8112	YJL8448	3 hr	3	515 to 650	none
YJL8112	YJL8449	3 hr	2	515 to 650	none
YJL8112	YJL8450	3 hr	2	515 to 650	none
YJL8113	YJL8451	3 hr	2	515 to 650	none
YJL8113	YJL8452	3 hr	2	515 to 650	none
YJL8113	YJL8453	3 hr	2	515 to 650	none
YJL8113	YJL8454	3 hr	2‡	515 to 650	ChrI Disomy; ChrIX Disomy; ChrXIII Disomy(?)*
YJL8113	YJL8455	3 hr	2	515 to 650	none
YJL8113	YJL8456	3 hr	2	515 to 650	none

Table S4 (continued)

aCGH analysis of uracil prototroph isolates.

YJL8113	YJL8457	3 hr	2‡	515 to 650	ChrXVI Disomy*
YJL8113	YJL8458	3 hr	2	515 to 650	none
YJL8363	YJL8459	3 hr	2	515 to 650	none
YJL8363	YJL8460	3 hr	2	515 to 650	none
YJL8363	YJL8461	3 hr	1.5†	515 to 650	Segmental Duplication of ChrIII_TEL-167kb; Segmental Duplication of ChrVII_541-820kb*
YJL8363	YJL8462	3 hr	1.5†	515 to 650	ChrXV Disomy*
YJL8363	YJL8463	3 hr	2	515 to 650	none
YJL8363	YJL8464	3 hr	2	515 to 650	none
YJL8363	YJL8465	3 hr	2	515 to 650	none
YJL8363	YJL8466	3 hr	2‡	515 to 650	Segmental Duplication(?) of ChrIV_TEL-515kb; Segmental Duplication(?) of ChrIV_650-875kb; Segmental Duplication(?) of ChrIV_985kb-TEL; Segmental Duplication(?) of ChrXII_947kb-TEL; Segmental Duplication(?) of ChrXIII_TEL-363 kb*
YJL8364	YJL8467	3 hr	2	515 to 650	none
YJL8364	YJL8468	3 hr	2	515 to 650	none
YJL8364	YJL8469	3 hr	1.5†	515 to 650	none*
YJL8364	YJL8470	3 hr	1.5†	515 to 650	ChrIV Disomy; ChrXIII Disomy*
YJL8364	YJL8471	3 hr	2	515 to 650	none
YJL8364	YJL8472	3 hr	2	515 to 650	none
YJL8364	YJL8473	3 hr	2	515 to 650	none
YJL8364	YJL8474	3 hr	2	515 to 650	none
YJL9139	YJL9287	3 hr	2	565 to 576	none
YJL9136	YJL9286	3 hr	2	545 to 592	none
YJL9115	YJL9275	3 hr	2	515 to 607	none
YJL9118	YJL9277	3 hr	2	515 to 650	none
YJL9121	YJL9279	3 hr	2	515 to 753	none
YJL9127	YJL9281	3 hr	2	515 to 875	none
YJL9130	YJL9283	3 hr	2	515 to 985	none

Table S4 (continued)

aCGH analysis of uracil prototroph isolates.

YJL9133	YJL9284	3 hr	2	515 to 1100	none
YJL9145	YJL9291	3 hr	2	607 to 753	none
YJL9147	YJL9293	3 hr	2	650 to 753	none
YJL9142	YJL9289	3 hr	2	576 to 713	none

* - aCGH suggests possible spontaneous diploid or mixed population. This is indicated either by the points in amplified region scattering at a non-quantile value (ie. 1.5) or by the points of an aneuploid chromosome scattering at a non-quantile value. aCGH will not be able to suggest possibly diploidization in cases of amplicons with quantile values or where there are no aneuploidies.

† - We suspect these isolates are diploids in which one copy of Chromosome IV bears an amplification

‡ - We suspect these isolates are diploids in which both copies of Chromosome IV bear an amplification
