# Supplementary material for: Single-Stranded Annealing Induced by Re-Initiation of Replication Origins Provides a Novel and Efficient Mechanism for Generating Copy Number Expansion via Non-Allelic Homologous Recombination
Source: PLoS Genet. 2013 Jan 3;9(1):e1003192. doi: 10.1371/journal.pgen.1003192 (PMC3536649; doi:10.1371/journal.pgen.1003192)
Supplement: Table S5 — Yeast strains used in this study. (PDF) [file pgen.1003192.s012.pdf]

Table S5

Yeast strains used in this study.

Strain	Genotype	Source
YJL6558	*MATa MCM7-2NLS ura3-52::{pGAL-ΔntCDC6,cdk2A, URA3} ORC2 orc6(S116A) leu2 trp1-289 ade2 ade3 bar1::LEU2 ChrIV*$_{567kb}$*::{kanMX, ade3-2p, ARS317} ars317::natMX*	Green et al.[1]
YJL6974	*MATa MCM7-2NLS ura3-52::{pGAL, URA3} ORC2 orc6(S116A) leu2 trp1-289 ade2 ade3 bar1::LEU2 ChrIV*$_{567kb}$*::{kanMX, ade3-2p, ARS317} ars317::natMX*	Green et al.[1]
YJL7445	*MATa rad1::TRP1 MCM7-2NLS ura3-52::{pGAL-ΔntCDC6,cdk2A, URA3} ORC2 orc6(S116A) leu2 trp1-289 ade2 ade3 bar1::LEU2 ChrIV*$_{567kb}$*::{kanMX, ade3-2p, ARS317} ars317::natMX*	This Study
YJL7451	*MATa rad51::TRP1 MCM7-2NLS ura3-52::{pGAL-ΔntCDC6,cdk2A, URA3} ORC2 orc6(S116A) leu2 trp1-289 ade2 ade3 bar1::LEU2 ChrIV*$_{567kb}$*::{kanMX, ade3-2p, ARS317} ars317::natMX*	This Study
YJL7607	*MATa MCM7-2NLS ura3-52 ORC2 orc6(S116A) leu2 trp1-289 ade2 ade3 bar1::LEU2 ChrIV*$_{567kb}$*::{kanMX, ade3-2p, ARS317} ars317::natMX*	This Study
YJL7852	*MATa MCM7-2NLS ura3-52::{Upstream URA3, URA3, Downstream URA3} ORC2 orc6(S116A) leu2 trp1-289 ade2 ade3 bar1::LEU2 ChrIV*$_{567kb}$*::{kanMX, ade3-2p, ARS317} ars317::natMX*	This Study
YJL7906	*MATa MCM7-2NLS ura3-ΔORF ORC2 orc6(S116A) leu2 trp1-289 ade2 ade3 bar1::LEU2 ChrIV*$_{567kb}$*::{kanMX, ade3-2p, ARS317} ars317::natMX*	This Study
YJL7929	*MATa MCM7-2NLS ura3-ΔORF ORC2 orc6(S116A) leu2 trp1-289 ade2 ade3 bar1::LEU2 ChrIV567kb::{kanMX, ade3-2p, ARS317} ars317::natMX YDRCTy2-1::{YDRCTy2-1 5' homology - RA3 - YDRCTy2-1 3' homology(v1), URA3}*	This Study
YJL7931	*MATa MCM7-2NLS ura3-ΔORF ORC2 orc6(S116A) leu2 trp1-289 ade2 ade3 bar1::LEU2 ChrIV567kb::{kanMX, ade3-2p, ARS317} ars317::natMX YDRCTy1-1::{YDRCTy1-1 5' homology(v1) - UR - YDRCTy1-1 3' homology, URA3}*	This Study
YJL7954	*MATa MCM7-2NLS ura3-ΔORF ORC2 orc6(S116A) leu2 trp1-289 ade2 ade3 bar1::LEU2 ChrIV567kb::{kanMX, ade3-2p, ARS317} ars317::natMX <YDRCdelta2, YDRCdelta3, YDRCdelta4, YDRCTy2-1, YDRCdelta5>Δ::RA3(v1)*	This Study

Table S5 (continued)

Yeast strains used in this study.

YJL7964	*MATa MCM7-2NLS ura3-ΔORF ORC2 orc6(S116A) leu2 trp1-289 ade2 ade3 bar1::LEU2 ChrIV567kb::{kanMX, ade3-2p, ARS317} ars317::natMX <YDRCdelta7, YDRCTy1-1, YDRCdelta8, YDRCsigma1, YDRCdelta9>Δ::UR(v1)*	This Study
YJL7986	*MATa MCM7-2NLS ura3-ΔORF ORC2 orc6(S116A) leu2 trp1-289 ade2 ade3 bar1::LEU2 ChrIV567kb::{kanMX, ade3-2p, ARS317} ars317::natMX <YDRCdelta2, YDRCdelta3, YDRCdelta4, YDRCTy2-1, YDRCdelta5>Δ::RA3(v1) YDRCTy1-1::{YDRCTy1-1 5' homology(v1) - UR - YDRCTy1-1 3' homology, URA3}*	This Study
YJL7993	*MATa MCM7-2NLS ura3-ΔORF ORC2 orc6(S116A) leu2 trp1-289 ade2 ade3 bar1::LEU2 ChrIV567kb::{kanMX, ade3-2p, ARS317} ars317::natMX <YDRCdelta2, YDRCdelta3, YDRCdelta4, YDRCTy2-1, YDRCdelta5>Δ::RA3(v1) YDRCdelta7, YDRCTy1-1, YDRCdelta8, YDRCsigma1, YDRCdelta9>Δ::UR(v1)*	This Study
YJL8027	*MATa MCM7-2NLS ura3- ΔORF::{tACT1-pGAL1/10-Δ ntCDC6,cdk2A-tCDC6, URA3} ORC2 orc6(S116A) leu2 trp1-289 ade2 ade3 bar1::LEU2 ChrIV*$_{567kb}$*::{kanMX, ade3-2p, ARS317} ars317::natMX*	This Study
YJL8031	*MATa MCM7-2NLS ura3-ΔORF::{tACT1-pGAL1/10-ΔntCDC6,cdk2A-tCDC6, URA3} ORC2 orc6(S116A) leu2 trp1-289 ade2 ade3 bar1::LEU2 ChrIV567kb::{kanMX, ade3-2p, ARS317} ars317::natMX <YDRCdelta2, YDRCdelta3, YDRCdelta4, YDRCTy2-1, YDRCdelta5>Δ::RA3(v1)*	This Study
YJL8035	*MATa MCM7-2NLS ura3-ΔORF::{tACT1-pGAL1/10-ΔntCDC6,cdk2A-tCDC6, URA3} ORC2 orc6(S116A) leu2 trp1-289 ade2 ade3 bar1::LEU2 ChrIV567kb::{kanMX, ade3-2p, ARS317} ars317::natMX <YDRCdelta7, YDRCTy1-1, YDRCdelta8, YDRCsigma1, YDRCdelta9>Δ::UR(v1)*	This Study
YJL8040	*MATa MCM7-2NLS ura3-ΔORF::{tACT1-pGAL1/10-ΔntCDC6,cdk2A-tCDC6, URA3} ORC2 orc6(S116A) leu2 trp1-289 ade2 ade3 bar1::LEU2 ChrIV567kb::{kanMX, ade3-2p, ARS317} ars317::natMX <YDRCdelta2, YDRCdelta3, YDRCdelta4, YDRCTy2-1, YDRCdelta5>Δ::RA3(v1) YDRCdelta7, YDRCTy1-1, YDRCdelta8, YDRCsigma1, YDRCdelta9>Δ::UR(v1)*	This Study
YJL8100/8101	*MATa MCM7-2NLS ura3 ::tACT1-pGAL1/10-Δ ntCDC6,cdk2A-tCDC6 ORC2 orc6(S116A) leu2 trp1-289 ade2 ade3 bar1::LEU2 ChrIV*$_{567kb}$*::{kanMX, ade3-2p, ARS317} ars317::natMX*	This Study
YJL8104	*MATa MCM7-2NLS ura3::tACT1-pGAL1/10-ΔntCDC6,cdk2A-tCDC6 ORC2 orc6(S116A) leu2 trp1-289 ade2 ade3 bar1::LEU2 ChrIV567kb::{kanMX, ade3-2p, ARS317} ars317::natMX <YDRCdelta2, YDRCdelta3, YDRCdelta4, YDRCTy2-1, YDRCdelta5>Δ::RA3(v1)*	This Study

Table S5 (continued)

Yeast strains used in this study.

YJL8108	*MATa MCM7-2NLS ura3::tACT1-pGAL1/10-ΔntCDC6,cdk2A-tCDC6 ORC2 orc6(S116A) leu2 trp1-289 ade2 ade3 bar1::LEU2 ChrIV567kb::{kanMX, ade3-2p, ARS317} ars317::natMX <YDRCdelta7, YDRCTy1-1, YDRCdelta8, YDRCsigma1, YDRCdelta9>Δ::UR(v1)*	This Study
YJL8112/8113	*MATa MCM7-2NLS ura3::tACT1-pGAL1/10-ΔntCDC6,cdk2A-tCDC6 ORC2 orc6(S116A) leu2 trp1-289 ade2 ade3 bar1::LEU2 ChrIV567kb::{kanMX, ade3-2p, ARS317} ars317::natMX <YDRCdelta2, YDRCdelta3, YDRCdelta4, YDRCTy2-1, YDRCdelta5>Δ::RA3(v1) YDRCdelta7, YDRCTy1-1, YDRCdelta8, YDRCsigma1, YDRCdelta9>Δ::UR(v1)*	This Study
YJL8230	*MATa MCM7-2NLS ura3-ΔORF ORC2 orc6(S116A) leu2 trp1-289 ade2 ade3 bar1::LEU2 ChrIV567kb::{kanMX, ade3-2p, ARS317} ars317::natMX YDRCTy2-1::{YDRCTy2-1 5' homology - RA3 - YDRCTy2-1 3' homology(v2), URA3}*	This Study
YJL8259	*MATa MCM7-2NLS ura3-ΔORF ORC2 orc6(S116A) leu2 trp1-289 ade2 ade3 bar1::LEU2 ChrIV567kb::{kanMX, ade3-2p, ARS317} ars317::natMX <YDRCdelta2, YDRCdelta3, YDRCdelta4, YDRCTy2-1, YDRCdelta5, tL(UAA)D, YDRCdelta6a, YDRCdelta7, YDRW034C-A, YDRCdelta6b, tQ(UUG)D1>Δ::RA3(v2)*	This Study
YJL8261	*MATa MCM7-2NLS ura3-ΔORF ORC2 orc6(S116A) leu2 trp1-289 ade2 ade3 bar1::LEU2 ChrIV567kb::{kanMX, ade3-2p, ARS317} ars317::natMX YDRCTy1-1::{YDRCTy1-1 5' homology(v2) - UR - YDRCTy1-1 3' homology, URA3}*	This Study
YJL8264	*MATa MCM7-2NLS ura3-ΔORF ORC2 orc6(S116A) leu2 trp1-289 ade2 ade3 bar1::LEU2 ChrIV567kb::{kanMX, ade3-2p, ARS317} ars317::natMX <YDRCdelta2, YDRCdelta3, YDRCdelta4, YDRCTy2-1, YDRCdelta5, tL(UAA)D, YDRCdelta6a, YDRCdelta7, YDRW034C-A, YDRCdelta6b, tQ(UUG)D1>Δ::RA3(v2) YDRCTy1-1::{YDRCTy1-1 5' homology(v2) - UR - YDRCTy1-1 3' homology, URA3}*	This Study
YJL8271	*MATa MCM7-2NLS ura3-ΔORF ORC2 orc6(S116A) leu2 trp1-289 ade2 ade3 bar1::LEU2 ChrIV567kb::{kanMX, ade3-2p, ARS317} ars317::natMX <tQ(UUG)D2, YDRCdelta7, YDRCTy1-1, YDRCdelta8, YDRCsigma1, YDRCdelta9>Δ::UR(v2)*	This Study

Table S5 (continued)

Yeast strains used in this study.

YJL8274	*MATa MCM7-2NLS ura3-ΔORF ORC2 orc6(S116A) leu2 trp1-289 ade2 ade3 bar1::LEU2 ChrIV567kb::{kanMX, ade3-2p, ARS317} ars317::natMX <YDRCdelta2, YDRCdelta3, YDRCdelta4, YDRCTy2-1, YDRCdelta5, tL(UAA)D, YDRCdelta6a, YDRCdelta7, YDRW034C-A, YDRCdelta6b, tQ(UUG)D1>Δ::RA3(v2) <tQ(UUG)D2, YDRCdelta7, YDRCTy1-1, YDRCdelta8, YDRCsigma1, YDRCdelta9>Δ::UR(v2)*	This Study
YJL8340	*MATa MCM7-2NLS URA3 ORC2 orc6(S116A) leu2 trp1-289 ade2 ade3 bar1::LEU2 ChrIV567kb::{kanMX, ade3-2p, ARS317} ars317::natMX <YDRCdelta2, YDRCdelta3, YDRCdelta4, YDRCTy2-1, YDRCdelta5, tL(UAA)D, YDRCdelta6a, YDRCdelta7, YDRW034C-A, YDRCdelta6b, tQ(UUG)D1>Δ::RA3(v2)*	This Study
YJL8342	*MATa MCM7-2NLS URA3 ORC2 orc6(S116A) leu2 trp1-289 ade2 ade3 bar1::LEU2 ChrIV567kb::{kanMX, ade3-2p, ARS317} ars317::natMX <tQ(UUG)D2, YDRCdelta7, YDRCTy1-1, YDRCdelta8, YDRCsigma1, YDRCdelta9>Δ::UR(v2)*	This Study
YJL8344	*MATa MCM7-2NLS URA3 ORC2 orc6(S116A) leu2 trp1-289 ade2 ade3 bar1::LEU2 ChrIV567kb::{kanMX, ade3-2p, ARS317} ars317::natMX <YDRCdelta2, YDRCdelta3, YDRCdelta4, YDRCTy2-1, YDRCdelta5, tL(UAA)D, YDRCdelta6a, YDRCdelta7, YDRW034C-A, YDRCdelta6b, tQ(UUG)D1>Δ::RA3(v2) <tQ(UUG)D2, YDRCdelta7, YDRCTy1-1, YDRCdelta8, YDRCsigma1, YDRCdelta9>Δ::UR(v2)*	This Study
YJL8355	*MATa MCM7-2NLS ura3::tACT1-pGAL1/10-ΔntCDC6,cdk2A-tCDC6 ORC2 orc6(S116A) leu2 trp1-289 ade2 ade3 bar1::LEU2 ChrIV567kb::{kanMX, ade3-2p, ARS317} ars317::natMX <YDRCdelta2, YDRCdelta3, YDRCdelta4, YDRCTy2-1, YDRCdelta5, tL(UAA)D, YDRCdelta6a, YDRCdelta7, YDRW034C-A, YDRCdelta6b, tQ(UUG)D1>Δ::RA3(v2)*	This Study
YJL8359	*MATa MCM7-2NLS ura3::tACT1-pGAL1/10-ΔntCDC6,cdk2A-tCDC6 ORC2 orc6(S116A) leu2 trp1-289 ade2 ade3 bar1::LEU2 ChrIV567kb::{kanMX, ade3-2p, ARS317} ars317::natMX <tQ(UUG)D2, YDRCdelta7, YDRCTy1-1, YDRCdelta8, YDRCsigma1, YDRCdelta9>Δ::UR(v2)*	This Study
YJL8363/8364	*MATa MCM7-2NLS ura3::tACT1-pGAL1/10-ΔntCDC6,cdk2A-tCDC6 ORC2 orc6(S116A) leu2 trp1-289 ade2 ade3 bar1::LEU2 ChrIV567kb::{kanMX, ade3-2p, ARS317} ars317::natMX <YDRCdelta2, YDRCdelta3, YDRCdelta4, YDRCTy2-1, YDRCdelta5, tL(UAA)D, YDRCdelta6a, YDRCdelta7, YDRW034C-A, YDRCdelta6b, tQ(UUG)D1>Δ::RA3(v2) <tQ(UUG)D2, YDRCdelta7, YDRCTy1-1, YDRCdelta8, YDRCsigma1, YDRCdelta9>Δ::UR(v2)*	This Study

Table S5 (continued)

Yeast strains used in this study.

YJL8407/8408	*MATa dnl4::TRP1 MCM7-2NLS ura3::tACT1-pGAL1/10-ΔntCDC6,cdk2A-tCDC6 ORC2 orc6(S116A) leu2 trp1-289 ade2 ade3 bar1::LEU2 ChrIV567kb::{kanMX, ade3-2p, ARS317} ars317::natMX <YDRCdelta2, YDRCdelta3, YDRCdelta4, YDRCTy2-1, YDRCdelta5, tL(UAA)D, YDRCdelta6a, YDRCdelta7, YDRW034C-A, YDRCdelta6b, tQ(UUG)D1>Δ::RA3(v2) <tQ(UUG)D2, YDRCdelta7, YDRCTy1-1, YDRCdelta8, YDRCsigma1, YDRCdelta9>Δ::UR(v2)*	This Study
YJL8409/8410	*MATa rad52::TRP1 MCM7-2NLS ura3::tACT1-pGAL1/10-ΔntCDC6,cdk2A-tCDC6 ORC2 orc6(S116A) leu2 trp1-289 ade2 ade3 bar1::LEU2 ChrIV567kb::{kanMX, ade3-2p, ARS317} ars317::natMX <YDRCdelta2, YDRCdelta3, YDRCdelta4, YDRCTy2-1, YDRCdelta5, tL(UAA)D, YDRCdelta6a, YDRCdelta7, YDRW034C-A, YDRCdelta6b, tQ(UUG)D1>Δ::RA3(v2) <tQ(UUG)D2, YDRCdelta7, YDRCTy1-1, YDRCdelta8, YDRCsigma1, YDRCdelta9>Δ::UR(v2)*	This Study
YJL8412/8413	*MATa rad51::TRP1 MCM7-2NLS ura3::tACT1-pGAL1/10-ΔntCDC6,cdk2A-tCDC6 ORC2 orc6(S116A) leu2 trp1-289 ade2 ade3 bar1::LEU2 ChrIV567kb::{kanMX, ade3-2p, ARS317} ars317::natMX <YDRCdelta2, YDRCdelta3, YDRCdelta4, YDRCTy2-1, YDRCdelta5, tL(UAA)D, YDRCdelta6a, YDRCdelta7, YDRW034C-A, YDRCdelta6b, tQ(UUG)D1>Δ::RA3(v2) <tQ(UUG)D2, YDRCdelta7, YDRCTy1-1, YDRCdelta8, YDRCsigma1, YDRCdelta9>Δ::UR(v2)*	This Study
YJL8415/8416	*MATa rad1::TRP1 MCM7-2NLS ura3::tACT1-pGAL1/10-ΔntCDC6,cdk2A-tCDC6 ORC2 orc6(S116A) leu2 trp1-289 ade2 ade3 bar1::LEU2 ChrIV567kb::{kanMX, ade3-2p, ARS317} ars317::natMX <YDRCdelta2, YDRCdelta3, YDRCdelta4, YDRCTy2-1, YDRCdelta5, tL(UAA)D, YDRCdelta6a, YDRCdelta7, YDRW034C-A, YDRCdelta6b, tQ(UUG)D1>Δ::RA3(v2) <tQ(UUG)D2, YDRCdelta7, YDRCTy1-1, YDRCdelta8, YDRCsigma1, YDRCdelta9>Δ::UR(v2)*	This Study
YJL8418/8419	*MATa msh3::TRP1 MCM7-2NLS ura3::tACT1-pGAL1/10-ΔntCDC6,cdk2A-tCDC6 ORC2 orc6(S116A) leu2 trp1-289 ade2 ade3 bar1::LEU2 ChrIV567kb::{kanMX, ade3-2p, ARS317} ars317::natMX <YDRCdelta2, YDRCdelta3, YDRCdelta4, YDRCTy2-1, YDRCdelta5, tL(UAA)D, YDRCdelta6a, YDRCdelta7, YDRW034C-A, YDRCdelta6b, tQ(UUG)D1>Δ::RA3(v2) <tQ(UUG)D2, YDRCdelta7, YDRCTy1-1, YDRCdelta8, YDRCsigma1, YDRCdelta9>Δ::UR(v2)*	This Study
YJL8421/8422	*MATa pol32::TRP1 MCM7-2NLS ura3::tACT1-pGAL1/10-ΔntCDC6,cdk2A-tCDC6 ORC2 orc6(S116A) leu2 trp1-289 ade2 ade3 bar1::LEU2 ChrIV567kb::{kanMX, ade3-2p, ARS317} ars317::natMX <YDRCdelta2, YDRCdelta3, YDRCdelta4, YDRCTy2-1, YDRCdelta5, tL(UAA)D, YDRCdelta6a, YDRCdelta7, YDRW034C-A, YDRCdelta6b, tQ(UUG)D1>Δ::RA3(v2) <tQ(UUG)D2, YDRCdelta7, YDRCTy1-1, YDRCdelta8, YDRCsigma1, YDRCdelta9>Δ::UR(v2)*	This Study

Table S5 (continued)

Yeast strains used in this study.

YJL8425/8426	*MATa MCM7-2NLS ura3-52::{pGAL-ΔntCDC6-cdk2A, URA3} ORC2 orc6(S116A) leu2 trp1-289 ade2 ade3 bar1::LEU2 ChrIV* $_{567kb}$ *::{hphMX, I-SceI cut site, ade3-2p, ARS317} ars317::natMX*	This Study
YJL8427/8428	*MATa MCM7-2NLS ura3-52::{pGAL, URA3} ORC2 orc6(S116A) leu2 trp1-289 ade2 ade3 bar1::LEU2 ChrIV* $_{567kb}$ *::{hphMX, I-SceI cut site, ade3-2p, ARS317} ars317::natMX*	This Study
YJL8799	*MATa MCM7-2NLS ura3::tACT1-pGAL1/10-Δ ntCDC6,cdk2A-tCDC6 ORC2 orc6(S116A) leu2 trp1-289 ade2 ade3 bar1::LEU2 ChrIV* $_{567kb}$ *::{kanMX, ade3-2p, ARS317} ars317::natMX YDRCTy2-1::{YDRCTy2-1 5' homology-YDRCTy2-1 3' homology(v2), URA3}*	This Study
YJL8807	*MATa MCM7-2NLS ura3::tACT1-pGAL1/10-ΔntCDC6,cdk2A-tCDC6 ORC2 orc6(S116A) leu2 trp1-289 ade2 ade3 bar1::LEU2 ChrIV* $_{567kb}$ *::{kanMX, ade3-2p, ARS317} ars317::natMX <YDRCdelta2, YDRCdelta3, YDRCdelta4, YDRCTy2-1, YDRCdelta5, tL(UAA)D, YDRCdelta6a, YDRCdelta7, YDRW034C-A, YDRCdelta6b, tQ(UUG)D1>Δ*	This Study
YJL8824	*MATa MCM7-2NLS ura3::tACT1-pGAL1/10-ΔntCDC6,cdk2A-tCDC6 ORC2 orc6(S116A) leu2 trp1-289 ade2 ade3 bar1::LEU2 ChrIV* $_{567kb}$ *::{kanMX, ade3-2p, ARS317} ars317::natMX <YDRCdelta2, YDRCdelta3, YDRCdelta4, YDRCTy2-1, YDRCdelta5, tL(UAA)D, YDRCdelta6a, YDRCdelta7, YDRW034C-A, YDRCdelta6b, tQ(UUG)D1>Δ YDRCTy1-1::{YDRCTy1-1 5' homology(v2)-YDRCTy1-1 3' homology, URA3}*	This Study
YJL8842	*MATa MCM7-2NLS ura3::tACT1-pGAL1/10-ΔntCDC6,cdk2A-tCDC6 ORC2 orc6(S116A) leu2 trp1-289 ade2 ade3 bar1::LEU2 ChrIV* $_{567kb}$ *::{kanMX, ade3-2p, ARS317} ars317::natMX <YDRCdelta2, YDRCdelta3, YDRCdelta4, YDRCTy2-1, YDRCdelta5, tL(UAA)D, YDRCdelta6a, YDRCdelta7, YDRW034C-A, YDRCdelta6b, tQ(UUG)D1>Δ <tQ(UUG)D2, YDRCdelta7, YDRCTy1-1, YDRCdelta8, YDRCsigma1, YDRCdelta9>Δ*	This Study
YJL9054	*MATa MCM7-2NLS ura3::tACT1-pGAL1/10-ΔntCDC6,cdk2A-tCDC6 ORC2 orc6(S116A) leu2 trp1-289 ade2 ade3 bar1::LEU2 ChrIV567kb::{kanMX, ade3-2p, ARS317} ars317::natMX <YDRCdelta2, YDRCdelta3, YDRCdelta4, YDRCTy2-1, YDRCdelta5, tL(UAA)D, YDRCdelta6a, YDRCdelta7, YDRW034C-A, YDRCdelta6b, tQ(UUG)D1>Δ <tQ(UUG)D2, YDRCdelta7, YDRCTy1-1, YDRCdelta8, YDRCsigma1, YDRCdelta9>Δ ChrIV515kb::{hphMX, RA3}*	This Study

Table S5 (continued)

Yeast strains used in this study.

YJL9056	*MATa MCM7-2NLS ura3::tACT1-pGAL1/10-ΔntCDC6,cdk2A-tCDC6 ORC2 orc6(S116A) leu2 trp1-289 ade2 ade3 bar1::LEU2 ChrIV567kb::{kanMX, ade3-2p, ARS317} ars317::natMX <YDRCdelta2, YDRCdelta3, YDRCdelta4, YDRCTy2-1, YDRCdelta5, tL(UAA)D, YDRCdelta6a, YDRCdelta7, YDRW034C-A, YDRCdelta6b, tQ(UUG)D1>Δ <tQ(UUG)D2, YDRCdelta7, YDRCTy1-1, YDRCdelta8, YDRCsigma1, YDRCdelta9>Δ ChrIV545kb::{hphMX, RA3}*	This Study
YJL9057	*MATa MCM7-2NLS ura3::tACT1-pGAL1/10-ΔntCDC6,cdk2A-tCDC6 ORC2 orc6(S116A) leu2 trp1-289 ade2 ade3 bar1::LEU2 ChrIV567kb::{kanMX, ade3-2p, ARS317} ars317::natMX <YDRCdelta2, YDRCdelta3, YDRCdelta4, YDRCTy2-1, YDRCdelta5, tL(UAA)D, YDRCdelta6a, YDRCdelta7, YDRW034C-A, YDRCdelta6b, tQ(UUG)D1>Δ <tQ(UUG)D2, YDRCdelta7, YDRCTy1-1, YDRCdelta8, YDRCsigma1, YDRCdelta9>Δ ChrIV565kb::{hphMX, RA3}*	This Study
YJL9060	*MATa MCM7-2NLS ura3::tACT1-pGAL1/10-ΔntCDC6,cdk2A-tCDC6 ORC2 orc6(S116A) leu2 trp1-289 ade2 ade3 bar1::LEU2 ChrIV567kb::{kanMX, ade3-2p, ARS317} ars317::natMX <YDRCdelta2, YDRCdelta3, YDRCdelta4, YDRCTy2-1, YDRCdelta5, tL(UAA)D, YDRCdelta6a, YDRCdelta7, YDRW034C-A, YDRCdelta6b, tQ(UUG)D1>Δ <tQ(UUG)D2, YDRCdelta7, YDRCTy1-1, YDRCdelta8, YDRCsigma1, YDRCdelta9>Δ ChrIV576kb::{hphMX, RA3}*	This Study
YJL9062	*MATa MCM7-2NLS ura3::tACT1-pGAL1/10-ΔntCDC6,cdk2A-tCDC6 ORC2 orc6(S116A) leu2 trp1-289 ade2 ade3 bar1::LEU2 ChrIV567kb::{kanMX, ade3-2p, ARS317} ars317::natMX <YDRCdelta2, YDRCdelta3, YDRCdelta4, YDRCTy2-1, YDRCdelta5, tL(UAA)D, YDRCdelta6a, YDRCdelta7, YDRW034C-A, YDRCdelta6b, tQ(UUG)D1>Δ <tQ(UUG)D2, YDRCdelta7, YDRCTy1-1, YDRCdelta8, YDRCsigma1, YDRCdelta9>Δ ChrIV607kb::{hphMX, RA3}*	This Study
YJL9067	*MATa MCM7-2NLS ura3::tACT1-pGAL1/10-ΔntCDC6,cdk2A-tCDC6 ORC2 orc6(S116A) leu2 trp1-289 ade2 ade3 bar1::LEU2 ChrIV567kb::{kanMX, ade3-2p, ARS317} ars317::natMX <YDRCdelta2, YDRCdelta3, YDRCdelta4, YDRCTy2-1, YDRCdelta5, tL(UAA)D, YDRCdelta6a, YDRCdelta7, YDRW034C-A, YDRCdelta6b, tQ(UUG)D1>Δ <tQ(UUG)D2, YDRCdelta7, YDRCTy1-1, YDRCdelta8, YDRCsigma1, YDRCdelta9>Δ ChrIV650kb::{hphMX, RA3}*	This Study
YJL9115/9116	*MATa MCM7-2NLS ura3::tACT1-pGAL1/10-ΔntCDC6,cdk2A-tCDC6 ORC2 orc6(S116A) leu2 trp1-289 ade2 ade3 bar1::LEU2 ChrIV567kb::{kanMX, ade3-2p, ARS317} ars317::natMX <YDRCdelta2, YDRCdelta3, YDRCdelta4, YDRCTy2-1, YDRCdelta5, tL(UAA)D, YDRCdelta6a, YDRCdelta7, YDRW034C-A, YDRCdelta6b, tQ(UUG)D1>Δ <tQ(UUG)D2, YDRCdelta7, YDRCTy1-1, YDRCdelta8, YDRCsigma1, YDRCdelta9>Δ ChrIV515kb::{hphMX, RA3} ChrIV607kb::{UR, TRP1}*	This Study

Table S5 (continued)

Yeast strains used in this study.

YJL9118/9119	*MATa MCM7-2NLS ura3::tACT1-pGAL1/10-∆ntCDC6,cdk2A-tCDC6 ORC2 orc6(S116A) leu2 trp1-289 ade2 ade3 bar1::LEU2 ChrIV567kb::{kanMX, ade3-2p, ARS317} ars317::natMX <YDRCdelta2, YDRCdelta3, YDRCdelta4, YDRCTy2-1, YDRCdelta5, tL(UAA)D, YDRCdelta6a, YDRCdelta7, YDRW034C-A, YDRCdelta6b, tQ(UUG)D1>∆ <tQ(UUG)D2, YDRCdelta7, YDRCTy1-1, YDRCdelta8, YDRCsigma1, YDRCdelta9>∆ ChrIV515kb::{hphMX, RA3} ChrIV650kb::{UR, TRP1}*	This Study
YJL9121/9122	*MATa MCM7-2NLS ura3::tACT1-pGAL1/10-∆ntCDC6,cdk2A-tCDC6 ORC2 orc6(S116A) leu2 trp1-289 ade2 ade3 bar1::LEU2 ChrIV567kb::{kanMX, ade3-2p, ARS317} ars317::natMX <YDRCdelta2, YDRCdelta3, YDRCdelta4, YDRCTy2-1, YDRCdelta5, tL(UAA)D, YDRCdelta6a, YDRCdelta7, YDRW034C-A, YDRCdelta6b, tQ(UUG)D1>∆ <tQ(UUG)D2, YDRCdelta7, YDRCTy1-1, YDRCdelta8, YDRCsigma1, YDRCdelta9>∆ ChrIV515kb::{hphMX, RA3} ChrIV753kb::{UR, TRP1}*	This Study
YJL9127/9128	*MATa MCM7-2NLS ura3::tACT1-pGAL1/10-∆ntCDC6,cdk2A-tCDC6 ORC2 orc6(S116A) leu2 trp1-289 ade2 ade3 bar1::LEU2 ChrIV567kb::{kanMX, ade3-2p, ARS317} ars317::natMX <YDRCdelta2, YDRCdelta3, YDRCdelta4, YDRCTy2-1, YDRCdelta5, tL(UAA)D, YDRCdelta6a, YDRCdelta7, YDRW034C-A, YDRCdelta6b, tQ(UUG)D1>∆ <tQ(UUG)D2, YDRCdelta7, YDRCTy1-1, YDRCdelta8, YDRCsigma1, YDRCdelta9>∆ ChrIV515kb::{hphMX, RA3} ChrIV875kb::{UR, TRP1}*	This Study
YJL9130/9131	*MATa MCM7-2NLS ura3::tACT1-pGAL1/10-∆ntCDC6,cdk2A-tCDC6 ORC2 orc6(S116A) leu2 trp1-289 ade2 ade3 bar1::LEU2 ChrIV567kb::{kanMX, ade3-2p, ARS317} ars317::natMX <YDRCdelta2, YDRCdelta3, YDRCdelta4, YDRCTy2-1, YDRCdelta5, tL(UAA)D, YDRCdelta6a, YDRCdelta7, YDRW034C-A, YDRCdelta6b, tQ(UUG)D1>∆ <tQ(UUG)D2, YDRCdelta7, YDRCTy1-1, YDRCdelta8, YDRCsigma1, YDRCdelta9>∆ ChrIV515kb::{hphMX, RA3} ChrIV985kb::{UR, TRP1}*	This Study
YJL9133/9134	*MATa MCM7-2NLS ura3::tACT1-pGAL1/10-∆ntCDC6,cdk2A-tCDC6 ORC2 orc6(S116A) leu2 trp1-289 ade2 ade3 bar1::LEU2 ChrIV567kb::{kanMX, ade3-2p, ARS317} ars317::natMX <YDRCdelta2, YDRCdelta3, YDRCdelta4, YDRCTy2-1, YDRCdelta5, tL(UAA)D, YDRCdelta6a, YDRCdelta7, YDRW034C-A, YDRCdelta6b, tQ(UUG)D1>∆ <tQ(UUG)D2, YDRCdelta7, YDRCTy1-1, YDRCdelta8, YDRCsigma1, YDRCdelta9>∆ ChrIV515kb::{hphMX, RA3} ChrIV1100kb::{UR, TRP1}*	This Study
YJL9136/9137	*MATa MCM7-2NLS ura3::tACT1-pGAL1/10-∆ntCDC6,cdk2A-tCDC6 ORC2 orc6(S116A) leu2 trp1-289 ade2 ade3 bar1::LEU2 ChrIV567kb::{kanMX, ade3-2p, ARS317} ars317::natMX <YDRCdelta2, YDRCdelta3, YDRCdelta4, YDRCTy2-1, YDRCdelta5, tL(UAA)D, YDRCdelta6a, YDRCdelta7, YDRW034C-A, YDRCdelta6b, tQ(UUG)D1>∆ <tQ(UUG)D2, YDRCdelta7, YDRCTy1-1, YDRCdelta8, YDRCsigma1, YDRCdelta9>∆ ChrIV545kb::{hphMX, RA3} ChrIV592kb::{UR, TRP1}*	This Study

Table S5 (continued)

Yeast strains used in this study.

YJL9139/9140	*MATa MCM7-2NLS ura3::tACT1-pGAL1/10-ΔntCDC6,cdk2A-tCDC6 ORC2 orc6(S116A) leu2 trp1-289 ade2 ade3 bar1::LEU2 ChrIV567kb::{kanMX, ade3-2p, ARS317} ars317::natMX <YDRCdelta2, YDRCdelta3, YDRCdelta4, YDRCTy2-1, YDRCdelta5, tL(UAA)D, YDRCdelta6a, YDRCdelta7, YDRW034C-A, YDRCdelta6b, tQ(UUG)D1>Δ <tQ(UUG)D2, YDRCdelta7, YDRCTy1-1, YDRCdelta8, YDRCsigma1, YDRCdelta9>Δ ChrIV565kb::{hphMX, RA3} ChrIV576kb::{UR, TRP1}*	This Study
YJL9142/9143	*MATa MCM7-2NLS ura3::tACT1-pGAL1/10-ΔntCDC6,cdk2A-tCDC6 ORC2 orc6(S116A) leu2 trp1-289 ade2 ade3 bar1::LEU2 ChrIV567kb::{kanMX, ade3-2p, ARS317} ars317::natMX <YDRCdelta2, YDRCdelta3, YDRCdelta4, YDRCTy2-1, YDRCdelta5, tL(UAA)D, YDRCdelta6a, YDRCdelta7, YDRW034C-A, YDRCdelta6b, tQ(UUG)D1>Δ <tQ(UUG)D2, YDRCdelta7, YDRCTy1-1, YDRCdelta8, YDRCsigma1, YDRCdelta9>Δ ChrIV576kb::{hphMX, RA3} ChrIV713kb::{UR, TRP1}*	This Study
YJL9145/9146	*MATa MCM7-2NLS ura3::tACT1-pGAL1/10-ΔntCDC6,cdk2A-tCDC6 ORC2 orc6(S116A) leu2 trp1-289 ade2 ade3 bar1::LEU2 ChrIV567kb::{kanMX, ade3-2p, ARS317} ars317::natMX <YDRCdelta2, YDRCdelta3, YDRCdelta4, YDRCTy2-1, YDRCdelta5, tL(UAA)D, YDRCdelta6a, YDRCdelta7, YDRW034C-A, YDRCdelta6b, tQ(UUG)D1>Δ <tQ(UUG)D2, YDRCdelta7, YDRCTy1-1, YDRCdelta8, YDRCsigma1, YDRCdelta9>Δ ChrIV607kb::{hphMX, RA3} ChrIV753kb::{UR, TRP1}*	This Study
YJL9147/9148	*MATa MCM7-2NLS ura3::tACT1-pGAL1/10-ΔntCDC6,cdk2A-tCDC6 ORC2 orc6(S116A) leu2 trp1-289 ade2 ade3 bar1::LEU2 ChrIV567kb::{kanMX, ade3-2p, ARS317} ars317::natMX <YDRCdelta2, YDRCdelta3, YDRCdelta4, YDRCTy2-1, YDRCdelta5, tL(UAA)D, YDRCdelta6a, YDRCdelta7, YDRW034C-A, YDRCdelta6b, tQ(UUG)D1>Δ <tQ(UUG)D2, YDRCdelta7, YDRCTy1-1, YDRCdelta8, YDRCsigma1, YDRCdelta9>Δ ChrIV650kb::{hphMX, RA3} ChrIV753kb::{UR, TRP1}*	This Study
YJL9149-9151	*MATa MCM7-2NLS ura3::tACT1-pGAL1/10-tCDC6 ORC2 orc6(S116A) leu2 trp1-289 ade2 ade3 bar1::LEU2 ChrIV567kb::{kanMX, ade3-2p, ARS317} ars317::natMX <YDRCdelta2, YDRCdelta3, YDRCdelta4, YDRCTy2-1, YDRCdelta5, tL(UAA)D, YDRCdelta6a, YDRCdelta7, YDRW034C-A, YDRCdelta6b, tQ(UUG)D1>Δ::RA3(v2) <tQ(UUG)D2, YDRCdelta7, YDRCTy1-1, YDRCdelta8, YDRCsigma1, YDRCdelta9>Δ::UR(v2)*	This Study

[1] Green, B.M. et al. *Science* **329,** 943–946 (2010)
