# Supplementary material for: Single-Stranded Annealing Induced by Re-Initiation of Replication Origins Provides a Novel and Efficient Mechanism for Generating Copy Number Expansion via Non-Allelic Homologous Recombination
Source: PLoS Genet. 2013 Jan 3;9(1):e1003192. doi: 10.1371/journal.pgen.1003192 (PMC3536649; doi:10.1371/journal.pgen.1003192)
Supplement: Table S6 — Plasmids used in this study. (PDF) [file pgen.1003192.s013.pdf]

Table S6

Plasmids used in this study.

Name	Description	Source
pRS304	*TRP1*	Sikorski, R.S. et al. *Genetics* **122(1),** 19-27 (1989)
pRS305	*LEU2*	Sikorski, R.S. et al. *Genetics* **122(1),** 19-27 (1989)
pRS306	*URA3*	Sikorski, R.S. et al. *Genetics* **122(1),** 19-27 (1989)
pAG26	*hphMX4, CEN-ARS, URA3*	Goldstein, A.L. et al. *Yeast* **15,** 1541-1553 (1999)
pSK179	*13kb EcoRI fragment containing URA3 in pBR322*	Natsoulis, G. et al. *Genetics* **123(2),** 269-279 (1989)
pJL124	*URA3 in pRS305*	This Study
pJL806	*pGAL1/10, URA3*	Nguyen, V.Q. et al. *Nature* **411,** 1068-1073 (2001)
pJL1488	*pGAL1/10-Δ ntCDC6,cdk2A-tCDC6, URA3*	Green, B.M. et al. *Mol Biol Cell* **17,** 2401-2414 (2005)
pKJF013	*YDRCTy2-1 replacement with RA3(v1)*	This Study
pKJF014	*YDRCTy1-1 replacement with UR(v1)*	This Study
pKJF017	*URA3 locus deletion (ura3-Δ ORF)*	This Study
pKJF019	*tACT1-pGAL1/10-Δ ntCDC6,cdk2A-tCDC6 (replace URA3 locus)*	This Study
pKJF020	*tACT1-pGAL1/10-tCDC6 (replace URA3 locus)*	This Study
pKJF021	*YDRCTy2-1 replacement with RA3(v2)*	This Study
pKJF022	*YDRCTy1-1 replacement with UR(v2)*	This Study
pKJF026	*YDRCdelta2* to *tQ(UUG)D1* deletion	This Study
pKJF027	*tQ(UUG)D2* to *YDRCdelta9* deletion	This Study
pKJF028	*UR+TRP1 Module*	This Study
pKJF029	*hphMX+RA3 Module*	This Study
