# Supplementary material for: Single-Stranded Annealing Induced by Re-Initiation of Replication Origins Provides a Novel and Efficient Mechanism for Generating Copy Number Expansion via Non-Allelic Homologous Recombination
Source: PLoS Genet. 2013 Jan 3;9(1):e1003192. doi: 10.1371/journal.pgen.1003192 (PMC3536649; doi:10.1371/journal.pgen.1003192)
Supplement: Table S7 — Oligonucleotides used in this study. (PDF) [file pgen.1003192.s014.pdf]

Table S7

Oligonucleotides used in this study. Listed 5' to 3', left to right.
Uppercase letters indicate sequence that anneals to the template during PCR or sequencing.
Lowercase letters indicate sequence added by PCR, either to provide homology for genomic integration or to provide restriction sites for cloning.
The bold, underlined sequence for OJL2317 us the cleavage site for I-SceI.
Subscript numbers are nucleotide coordinates provided by the Saccharomyces Genome Database (version R64-1-1)

Name	Sequence	Purpose
OJL2177	gtcatctaggccgcggAGATGCTAAGAGATAGTGATG	$ChrV_{115801-116166}$ for Homology Left in pKJF017
OJL2178	gtcatctagggaattcGATTTATCTTCGTTTCCTGC	$ChrV_{115801-116166}$ for Homology Left in pKJF017
OJL2180	gtcatctagggtcgacCTTTACAGTCCTGTCTTATTG	$ChrV_{116971-117211}$ for Homology Right in pKJF017
OJL2181	gtcatctagggaattcAAAACTGTATTATAAGTAAATGCATG	$ChrV_{116971-117211}$ for Homology Right in pKJF017
OJL2204	gtcatctagagccggcAGATGCTAAGAGATAGTGATG	$ChrV_{115801-116166}$ for Homology Left in pKJF019 and pKJF020
OJL2205	gtcatctagggagctcGATTTATCTTCGTTTCCTGC	$ChrV_{115801-116166}$ for Homology Left in pKJF019 and pKJF020
OJL2202	gtcatctagggagctcTATGATACACGGTCCAATGG	$ChrVI_{52968-53259}$ for for *ACT1* terminator in pKJF019 and pKJF020
OJL2203	gtcatctaggccgcggTCTCTGCTTTTGTGCGCG	$ChrVI_{52968-53259}$ for for *ACT1* terminator in pKJF019 and pKJF020
OJL2206	gtcatctaggctcgagAAAACTGTATTATAAGTAAATGCATG	$ChrV_{116971-117211}$ for Homology Right in pKJF019 and pKJF020
OJL2207	gtcatctaggggtaccCTTTACAGTCCTGTCTTATTG	$ChrV_{116971-117211}$ for Homology Right in pKJF019 and pKJF020
OJL2158	gtcatctaggccgcggGGAAGAAACAGGGAGATAAGG	$ChrIV_{512151-5129128}$ for Homology Left in pKJF013, pKJF021 and pKJF026
OJL2159	gctcaaatgggatccGTAGCCTTCTAAAATCTGAGG	$ChrIV_{512151-5129128}$ for Homology Left in pKJF013, pKJF021 and pKJF026
OJL2167	gtcatctagcccgggGCATCTGTGCGGTATTTCAC	*RA3* element in pKJF013, pKJF021, and pKJF029
OJL2170	gtcatctagggatccACAGTTAAGCCGCTAAAGGC	*RA3* element in pKJF013, pKJF021, and pKJF029
OJL2160	cgtcatatggatccgctcaaatgcccgggGCAAGTGATTTTACGGAGGG	$ChrIV_{519721-520163}$ for Homology Right in pKJF013
OJL2161	gctcaaatggtcgacCAAGAATAGGTGGATAATACGG	$ChrIV_{519721-520163}$ for Homology Right in pKJF013
OJL2265	cgtcatatggcccgggTTTCTATTTTTTAACCCTCTTG	$ChrIV_{521044-521567}$ for Homology Right in pKJF021, pKJF026, and pKJF029
OJL2266	gctcaaatggtcgacATGTACGCATAAATTTGAGAAG	$ChrIV_{521044-521567}$ for Homology Right in pKJF021, pKJF026, and pKJF029
OJL2162	gtcatctaggccgcggACTGATGGTTCAACAGAGAAGC	$ChrIV_{645119-645467}$ for Homology Left in pKJF014
OJL2163	cgtcatatggaattcgctcaaatgggatccCTTCCCTAGCTGAACTACCC	$ChrIV_{645119-645467}$ for Homology Left in pKJF014
OJL2267	gtcatctaggccgcggCGTCCGCATCAATGGATAAG	$ChrIV_{644738-645152}$ for Homology Left in pKJF022, pKJF027, and pKJF028
OJL2268	ccatatgacgggatccTTTTTAACTGTGGCTTCTCTG	$ChrIV_{644738-645152}$ for Homology Left in pKJF022, pKJF027, and pKJF028
OJL2168	gtcatctagggatccGAGCAGATTGTACTGAGAGTG	*UR* element in pKJF014, pKJF024, and pKJF029
OJL2171	gctcaaatggaattctcaGGGTGTCATAATCAACCAATCG	*UR* element in pKJF014, pKJF024, and pKJF029
OJL2164	gtcatctaggaattcCAATGAGCGCGCCATTATCG	$ChrIV_{651992-652532}$ for Homology Right in pKJF014, pKJF022, and pKJF027
OJL2165	gctcaaatggtcgacCTTCTCGTCACGGAAGACCG	$ChrIV_{651992-652532}$ for Homology Right in pKJF014, pKJF022, and pKJF027
OJL2543	gtcatctagggaattcGAGCAGATTGTACTGAGAGTG	*TRP1* selectable marker in pKJF028
OJL2544	gtcatctagggtcgacGCATCTGTGCGGTATTTCAC	*TRP1* selectable marker in pKJF028
OJL2097	gaactgaaggaaatagtaacggattatttaggtGAGCAGATTGTACTGAGAGTG	*dnl4::TRP1,* Step 1
OJL2098	caaaaaattaagcctccgcaaaacgcaccaGCATCTGTGCGGTATTTCAC	*dnl4::TRP1,* Step 1
OJL2099	gtggaaaataaatactaaaataaaaatctaGAACTGAAGGAAATAGTAACGG	*dnl4::TRP1,* Step 2
OJL2100	tacatatgtaggatagtattaaataaacttCAAAAAATTAAGCCTCCGC	*dnl4::TRP1,* Step 2
OJL2117	gaaggttctggtggctttggtgtgttgttgGAGCAGATTGTACTGAGAGTG	*rad52::TRP1,* Step 1
OJL2118	aatgatgcaaattttttatttgtttcggccaggaagcgttGCATCTGTGCGGTATTTCAC	*rad52::TRP1,* Step 1
OJL2119	atgcaaacaaggaggttgccaagaactgctGAAGGTTCTGGTGGCTTTGG	*rad52::TRP1,* Step 2
OJL2120	aactagaggattttggagtaataaatAATGATGCAAATTTTTTATTTGTTTCGGC	*rad52::TRP1,* Step 2

Table S7 (continued)

OJL2105	aatattgcactatcctgttgaaaatatctttccagGAGCAGATTGTACTGAGAGTG	*rad1::TRP1*, Step 1
OJL2106	tgatgttttaacagggttcgttaaattaaacaatattGCATCTGTGCGGTATTTCAC	*rad1::TRP1*, Step 1
OJL2107	tggtagagcatttgctaaatgtgtaaaaatAATATTGCACTATCCTGTTG	*rad1::TRP1*, Step 2
OJL2108	attttcactatagttaatcgcattttatacTGATGTTTTAACAGGGTTCG	*rad1::TRP1*, Step 2
OJL2113	gttaaaggcctactaatttgttatcgtcatGAGCAGATTGTACTGAGAGTG	*rad51::TRP1*, Step 1
OJL2114	cctgtgtaaataaatagagacaagagaccaaatacGCATCTGTGCGGTATTTCAC	*rad51::TRP1*, Step 1
OJL2115	tgtagcgacaaagagcagacgtagttatttGTTAAAGGCCTACTAATTTG	*rad51::TRP1*, Step 2
OJL2116	cctaagaaaaagaggagaattgaaagtaaaCCTGTGTAAATAAATAGAGACAAG	*rad51::TRP1*, Step 2
OJL2182	caaccagaaataggctttagttaactcaatcggtaattaGAGCAGATTGTACTGAGAGTG	*pol32::TRP1*, Step 1
OJL2183	cacaattagtaatggaaagtgtttggaaaaaaaaagaagaGCATCTGTGCGGTATTTCAC	*pol32::TRP1*, Step 1
OJL2184	cagatcagctcgaaataatatttcacattaactaaCAACCAGAAATAGGCTTTAGTTAAC	*pol32::TRP1*, Step 2
OJL2185	atcacgtaagttgacatttgtattatacattacatCACAATTAGTAATGGAAAGTGTTTG	*pol32::TRP1*, Step 2
OJL2192	agtgcaaatagatttattttgttgaatctattaacaataGAGCAGATTGTACTGAGAGTG	*msh3::TRP1*, Step 1
OJL2193	gtctgataatgctgcatttagaacatacgtaccatccgcaGCATCTGTGCGGTATTTCAC	*msh3::TRP1*, Step 1
OJL2194	aatgaacctaaactggtacttttgagagccaaaagcAGTGCAAATAGATTTATTTTGTTG	*msh3::TRP1*, Step 2
OJL2195	acgagaaagtgaatgaaaaatataatatcatttattGTCTGATAATGCTGCATTTAGAAC	*msh3::TRP1*, Step 2
OJL2317	gcgataacatacaatggaaaatcacgttcagcgctccgcggccgtccgcttgcttttccaacaatctgtgctttagctcacgtaagt actccggatcgat**ctatattaccctgttatccctagcgtaact**CCCGGGTTAATTAAGGCG	hphMX + I-SceI cut site (replaces kanMX in the reporter cassette)
OJL2318	CTGCGCACTTAACTTCGC	hphMX + I-SceI cut site (replaces kanMX in the reporter cassette)
OJL2636	atcctcagattttagaaggctacttcaaaaGGATGGCGGCGTTAGTATCG	*ChrIV* $_{515kb}$ *::{hphMX, RA3}*, Step 1
OJL2637	agaaaaataaaacaagagggttaaaaaataGCATCTGTGCGGTATTTCAC	*ChrIV* $_{515kb}$ *::{hphMX, RA3}*, Step 1
OJL2638	gacatacattagtgacatatgctagaaattATCCTCAGATTTTAGAAGGC	*ChrIV* $_{515kb}$ *::{hphMX, RA3}*, Step 2
OJL2639	gcgaggggctgtgcaattcaaagccaagaaAGAAAAATAAAACAAGAGGG	*ChrIV* $_{515kb}$ *::{hphMX, RA3}*, Step 2
OJL2640	aaactaactttgtcgacacctattttaaacGGATGGCGGCGTTAGTATCG	*ChrIV* $_{545kb}$ *::{hphMX, RA3}*, Step 1
OJL2641	cataaaaattatagcccgcgtttgatacttGCATCTGTGCGGTATTTCAC	*ChrIV* $_{545kb}$ *::{hphMX, RA3}*, Step 1
OJL2642	gtcatattgtaaaaactaaatggaagacagAAACTAACTTTGTCGACACC	*ChrIV* $_{545kb}$ *::{hphMX, RA3}*, Step 2
OJL2643	ggaaatagactccattgcattgattgctctCATAAAAATTATAGCCCGCG	*ChrIV* $_{545kb}$ *::{hphMX, RA3}*, Step 2
OJL2644	tatgtattcaaatattttcgggaaagagatGGATGGCGGCGTTAGTATCG	*ChrIV* $_{565kb}$ *::{hphMX, RA3}*, Step 1
OJL2645	ttttttaaatttttaagtgtcgttacttttGCATCTGTGCGGTATTTCAC	*ChrIV* $_{565kb}$ *::{hphMX, RA3}*, Step 1
OJL2646	gaacaaataaaaacatttatttaaaaatttTATGTATTCAAATATTTTCG	*ChrIV* $_{565kb}$ *::{hphMX, RA3}*, Step 2
OJL2647	tcaaaagactgagtaaataaagtattgtgaTTTTTTAAATTTTTAAGTGT	*ChrIV* $_{565kb}$ *::{hphMX, RA3}*, Step 2
OJL2648	tatcatagccagttagcgggaacgacttcaGGATGGCGGCGTTAGTATCG	*ChrIV* $_{576kb}$ *::{hphMX, RA3}*, Step 1
OJL2649	tctgcctaaagcatggatagtccatttagcGCATCTGTGCGGTATTTCAC	*ChrIV* $_{576kb}$ *::{hphMX, RA3}*, Step 1; *ChrIV* $_{576kb}$ *::{UR, TRP1}*, Step 1
OJL2650	atctgttatactacaaaacaattacttctaTATCATAGCCAGTTAGCGGG	*ChrIV* $_{576kb}$ *::{hphMX, RA3}*, Step 2; *ChrIV* $_{576kb}$ *::{UR, TRP1}*, Step 2
OJL2651	tgatgttacacccaatcaccgcgcttcgccTCTGCCTAAAGCATGGATAG	*ChrIV* $_{576kb}$ *::{hphMX, RA3}*, Step 2; *ChrIV* $_{576kb}$ *::{UR, TRP1}*, Step 2
OJL2652	aagtaaaattaatagaatccagtttccagtGGATGGCGGCGTTAGTATCG	*ChrIV* $_{607kb}$ *::{hphMX, RA3}*, Step 1
OJL2653	gagatatttattatttagcctgtaccgatcGCATCTGTGCGGTATTTCAC	*ChrIV* $_{607kb}$ *::{hphMX, RA3}*, Step 1; *ChrIV* $_{607kb}$ *::{UR, TRP1}*, Step 1
OJL2654	aaaaaaagaaaaagatatcttggtttgaatAAGTAAAATTAATAGAATCC	*ChrIV* $_{607kb}$ *::{hphMX, RA3}*, Step 2; *ChrIV* $_{607kb}$ *::{UR, TRP1}*, Step 2
OJL2655	acaagaaaacgaaaagaataaataaaagtaGAGATATTTATTATTTAGCC	*ChrIV* $_{607kb}$ *::{hphMX, RA3}*, Step 2; *ChrIV* $_{607kb}$ *::{5'URA3, TRP1}*, Step 2
OJL2656	gatggttcaacagagaagccacagttaaaaGGATGGCGGCGTTAGTATCG	*ChrIV* $_{650kb}$ *::{hphMX, RA3}*, Step 1
OJL2657	ttacagaacgataatggcgcgctcattgtaGCATCTGTGCGGTATTTCAC	*ChrIV* $_{650kb}$ *::{hphMX, RA3}*, Step 1; *ChrIV* $_{650kb}$ *::{UR, TRP1}*, Step 1
OJL2658	cacattctctgaaaaaaaaaaaaagtaactGATGGTTCAACAGAGAAGCC	*ChrIV* $_{650kb}$ *::{hphMX, RA3}*, Step 2; *ChrIV* $_{650kb}$ *::{UR, TRP1}*, Step 2
OJL2659	attagctcgttatgtgaatgactatttactTTACAGAACGATAATGGCGC	*ChrIV* $_{650kb}$ *::{hphMX, RA3}*, Step 2; *ChrIV* $_{650kb}$ *::{UR, TRP1}*, Step 2

Table S7 (continued)

OJL2697	tatcatagccagttagcgggaacgacttcaTGAGAGTGCACCATACCACC	*ChrIV* $_{576kb}$ *::{UR, TRP1},* Step 1
OJL2698	cgtggccaactgaagtaatctttagccgttTGAGAGTGCACCATACCACC	*ChrIV* $_{592kb}$ *::{UR, TRP1},* Step 1
OJL2699	caagtgcaattgccgttttgcgcggcgataGCATCTGTGCGGTATTTCAC	*ChrIV* $_{592kb}$ *::{UR, TRP1},* Step 1
OJL2700	gcgtaatccactcgggaagggcgcagctgcCGTGGCCAACTGAAGTAATC	*ChrIV* $_{592kb}$ *::{UR, TRP1},* Step 2
OJL2701	ataccctgctgcacagcgccgttggggtctCAAGTGCAATTGCCGTTTTG	*ChrIV* $_{592kb}$ *::{UR, TRP1},* Step 2
OJL2702	aagtaaaattaatagaatccagtttccagtTGAGAGTGCACCATACCACC	*ChrIV* $_{607kb}$ *::{UR, TRP1},* Step 1
OJL2703	gatggttcaacagagaagccacagttaaaaTGAGAGTGCACCATACCACC	*ChrIV* $_{650kb}$ *::{UR, TRP1},* Step 1
OJL2704	tagaggcatgatagaagacgacaacggaatTGAGAGTGCACCATACCACC	*ChrIV* $_{713kb}$ *::{UR, TRP1},* Step 1
OJL2705	ccattgtaaaaatgcctccaacgctcaatgGCATCTGTGCGGTATTTCAC	*ChrIV* $_{713kb}$ *::{UR, TRP1},* Step 1
OJL2706	aagtagtttatttacgattttttaatccagTAGAGGCATGATAGAAGACG	*ChrIV* $_{713kb}$ *::{UR, TRP1},* Step 2
OJL2707	ttccacctacttttattcgtgattactgagCCATTGTAAAAATGCCTCCA	*ChrIV* $_{713kb}$ *::{UR, TRP1},* Step 2
OJL2708	ttaagagaataaattctttcaaaaatgtagTGAGAGTGCACCATACCACC	*ChrIV* $_{753kb}$ *::{UR, TRP1},* Step 1
OJL2709	aatattcttttgatttccacataacttgctGCATCTGTGCGGTATTTCAC	*ChrIV* $_{753kb}$ *::{UR, TRP1},* Step 1
OJL2710	tttgaagcaaaatgtaaaaaaagaatattaTTAAGAGAATAAATTCTTTC	*ChrIV* $_{753kb}$ *::{UR, TRP1},* Step 2
OJL2711	ataactatttattatataatagaatcaactAATATTCTTTTGATTTCCAC	*ChrIV* $_{753kb}$ *::{UR, TRP1},* Step 2
OJL2716	attccattgttgacaaaggctatgatattaTGAGAGTGCACCATACCACC	*ChrIV* $_{875kb}$ *::{UR, TRP1},* Step 1
OJL2717	attttgggaaaattgtcgggatattagtgtGCATCTGTGCGGTATTTCAC	*ChrIV* $_{875kb}$ *::{UR, TRP1},* Step 1
OJL2718	aaatggatgaaatttgagataattgttgggATTCCATTGTTGACAAAGGC	*ChrIV* $_{875kb}$ *::{UR, TRP1},* Step 2
OJL2719	aaaaaatagctctttctgagaattctttgaATTTTGGGAAAATTGTCGGG	*ChrIV* $_{875kb}$ *::{UR, TRP1},* Step 2
OJL2720	acacacatgcaacttgttggaataaaaatcTGAGAGTGCACCATACCACC	*ChrIV* $_{985kb}$ *::{UR, TRP1},* Step 1
OJL2721	actaaaccacttgcgctcacaattgttggaGCATCTGTGCGGTATTTCAC	*ChrIV* $_{985kb}$ *::{UR, TRP1},* Step 1
OJL2722	tattatgacacacatctaaacgttaataaaACACACATGCAACTTGTTGG	*ChrIV* $_{985kb}$ *::{UR, TRP1},* Step 2
OJL2723	ggcccaacgatggcaacgttggattttaccACTAAACCACTTGCGCTCAC	*ChrIV* $_{985kb}$ *::{UR, TRP1},* Step 2
OJL2724	aagtgttgggattgcattggtgataaatccTGAGAGTGCACCATACCACC	*ChrIV* $_{1100kb}$ *::{UR, TRP1},* Step 1
OJL2725	tttaaaagcaactgaaacgcacggattgatGCATCTGTGCGGTATTTCAC	*ChrIV* $_{1100kb}$ *::{UR, TRP1},* Step 1
OJL2726	atttaattaagataggtaactcttgtgaatAAGTGTTGGGATTGCATTGG	*ChrIV* $_{1100kb}$ *::{UR, TRP1},* Step 2
OJL2727	tgacataaatgataagaatgcgttatcaaaTTTAAAAGCAACTGAAACGC	*ChrIV* $_{1100kb}$ *::{UR, TRP1},* Step 2
OJL2228	GCCAGAAAACAGATCTTATACC	Primer 1 for Junction PCR
OJL2269	CCGATGTGACTAATACTTGAGC	Primer 2 for Junction PCR
OJL2311	CGGAATTGACTGAGTTCTTGC	Primer 3 for Junction PCR
OJL2227	AGTAGAAATGAGGGAAGAAGC	Primer 4 for Junction PCR
OJL2449	GAGGACATACGTCGTGAAGC	*MAK21* Probe
OJL2450	AGGCTTCTACTCGTTCAACG	*MAK21* Probe
OJL2231	AGGACGACTGGAGTGATAGG	*YOS9* Probe
OJL2232	AAACACATGAGTCACCAGGC	*YOS9* Probe
