# Supplementary material for: Single-Stranded Annealing Induced by Re-Initiation of Replication Origins Provides a Novel and Efficient Mechanism for Generating Copy Number Expansion via Non-Allelic Homologous Recombination
Source: PLoS Genet. 2013 Jan 3;9(1):e1003192. doi: 10.1371/journal.pgen.1003192 (PMC3536649; doi:10.1371/journal.pgen.1003192)
Supplement: Text S1 — Detailed descriptions of all methods and materials used in this study. (PDF) [file pgen.1003192.s015.pdf]

<u>Text S1</u>

Oligonucleotides

Oligonucleotides used in the plasmid and yeast strain constructions described below are listed in Table S7.

Plasmids

All plasmids used for strain construction are listed in Table S6.

pJL124 contains a BglII-BamHI *URA3* fragment sub-cloned from pSK179 [1] into pRS305 [2]. The BglII end of the fragment is ligated to the BamHI site in pRS305 polylinker, and the BamHI end of the fragment is filled in and blunt-end ligated to a filled in XhoI site in the polylinker.

pKJF017 is used to replace *ura3-52* with *ura3-ΔORF* by loop-in/loop-out replacement. It effectively consists of the following elements: Homology Left (SacII to EcoRI of PCR product from YJL6558 [3] genomic DNA using OJL2177 and OJL2178), Homology Right (EcoRI-SalI of PCR product from YJL6558 genomic DNA using OJL2180 and OJL2181), and vector backbone (SalI to SacII of pRS306 [2]).

pKJF019 is used to replace *ura3-ΔORF* or *URA3* with *tACT1-pGAL1/10-ΔntCDC6,cdk2A-tCDC6* by loop-in/loop-out or direct replacement, respectively. It was generated from pJL1488 [4] by introducing a Homology Left element (NgoMIV to SacI of PCR product from pKJF017 plasmid DNA using OJL2204 and OJL2205) and an *ACT1* terminator element (SacI to SacII of PCR product from YJL6974 [3] genomic DNA using OJL2202 and OJL2203) between the NgoMIV and SacII sites of pJL1488. Then a Homology Right element

(XhoI to Asp718I of PCR product from pKJF017 plasmid DNA using OJL2206 and OJL2207) was inserted between the XhoI and Asp718I sites of the resulting plasmid.

pKJF020 is used to replace *ura3-ΔORF* or *URA3* with *tACT1-pGAL1/10-tCDC6* by loop-in/loop-out or direct replacement, respectively. It was generated from pKJF019 by digesting with BamHI and SpeI, filling in the overhangs with Klenow, then ligating the blunted ends together to re-circularize. This removes the Δ*ntCDC6,cdk2A* element.

pKJF013 and pKJF021 replace *YDRCTy2-1* with version 1 or version 2 of the *3'URA3* element (*RA3*), respectively. These effectively consist of the following elements: Homology Left (SacII to BamHI of PCR product from YJL6974 genomic DNA using OJL2158 and OJL2159), *RA3* (BamHI to XmaI of PCR product from pRS306 plasmid DNA using OJL2167 and OJL2170), Homology Right (XmaI to SalI of PCR product from YJL6974 genomic DNA using OJL2160 and OJL2161 for pKJF013, and OJL2265 and OJL2266 for pKJF021), and vector backbone (SalI to SacII of pRS306).

pKJF014 and pKJF022 replace *YDRCTy1-1* with version 1 or version 2 of the 5'*URA3* element (*UR*), respectively. These effectively consist of the following elements: Homology Left (SacII to BamHI of PCR product from YJL6974 genomic DNA using OJL2162 and OJL2163 for pKJF014, and OJL2267 and OJL2268 for pKJF022), *UR* (BamHI to EcoRI of PCR product from pRS306 plasmid DNA using OJL2168 and OJL2171), Homology Right (EcoRI to SalI of PCR product from YJL6974 genomic DNA using OJL2164 and OJL2165), and vector backbone (SalI to SacII of pRS306).

pKJF026 deletes the sequence between sequence from *YDRCdelta2* to *tQ(UUG)D1* through a loop-in/loop-out replacement. It effectively consists of the following elements: Homology Left (SacII to BamHI of PCR product from YJL6974 genomic DNA using OJL2158

and OJL2159), a BamHI-XmaI linker sequence (5'-GGATCCGCTCAAATGCCCGGG-3'), Homology Right (XmaI to SalI of PCR product from YJL6974 genomic DNA using OJL2265 and OJL2266), and vector backbone (SalI to SacII of pRS306).

pKJF027 deletes the sequence between sequence from *tQ(UUG)D2* to *YDRCdelta9* through a loop-in/loop-out replacement. It effectively consists of the following elements: Homology Left (SacII to BamHI of PCR product from YJL6974 genomic DNA using OJL2267 and OJL2268), a BamHI-EcoRI linker sequence (5'-GGATCCCATTTGAGCGAATTC-3'), Homology Right (EcoRI to SalI of PCR product from YJL6974 genomic DNA using OJL2164 and OJL2165), and vector backbone (SalI to SacII of pRS306).

pKJF028 is used as a PCR template for the *UR* element + *TRP1* selectable marker module for integration at various genomic locations. It effectively consists of the following elements: Homology Left (SacII to BamHI of PCR product from YJL6974 genomic DNA using OJL2267 and OJL2268), *UR* (BamHI to EcoRI of PCR product from pRS306 plasmid DNA using OJL2168 and OJL2171), *TRP1* (EcoRI to SalI of PCR product from pRS304[2] plasmid DNA using OJL2543 and OJL2544), and vector backbone (SalI to SacII of pRS306).

pKJF029 is used as a PCR template for the *RA3* element + *hphMX* selectable marker module for integration at various genomic locations. It effectively consists of the following elements: *hphMX* (SacII to BamHI of pAG26[5]), *RA3* (BamHI to XmaI of PCR product from pRS306 plasmid DNA using OJL2167 and OJL2170), Homology Right (XmaI to SalI of PCR product from YJL6974 genomic DNA using OJL2265 and OJL2266), and vector backbone (SalI to SacII of pRS306).

Strains

All strains used in this study have their genotypes listed in Table S5. All strains used in this study are derived from YJL6558 or YJL6974 (see below for details). As we reported in Green et al. 2010, the *ORC6* gene in these strains has a recently discovered mutation that changes serine 116 to alanine. This *orc6(S116A)* allele is not necessary for the preferential re-initiation of *ARS317* in our "MC_{2A}" strains (i.e. strains with *MCM7-2NLS* and *pGAL-ΔntCDC6,cdk2A*) but enhances the level of initiation 2-3x relative to the wild type *ORC6* allele (data not shown).

Strains used to determine whether or not homology in *cis* is necessary and sufficient for RRIGA (Figure 1, Figure S2, Figure S3) were derived from YJL7906, which was in turn derived as follows from YJL6974 (*MATa MCM7-2NLS ura3-52::{pGAL, URA3} ORC2 orc6(S116A) leu2 trp1-289 ade2 ade3 bar1::LEU2* $ChrIV_{567kb}$*::{kanMX, ade3-2p, ARS317} ars317::natMX*) [3]. YJL7607 was derived from YJL6974 by selection on 5-flouroortic acid for loss of pJL806 integrated at *ura3-52*. YJL7906 was derived from YJL7607 by loop-in/loop-out gene replacement of *ura3-52* with *ura3-ΔORF* using SmaI-linearized pKJF017.

YJL8100, YJL8104, YJL8108, and YJL8112 were derived from YJL7906, YJL7954, YJL7964, and YJL7993, respectively, by loop-in/loop-out gene replacement of *ura3-ΔORF* with *tACT1-pGAL1/10-ΔntCDC6,cdk2A-tCDC6* using SmaI-linearized pKJF019. YJL7954 was derived from YJL7906 by loop-in/loop-out gene replacement of *YDRCTy2-1* with version 1 of *RA3* using BglII-linearized pKJF013. YJL7964 and YJL7993 were derived from YJL7906 and YJL7954, respectively, by loop-in/loop-out gene replacement of *YDRCTy1-1* with version 1 of UR using SpeI-linearized pKJF014.

YJL8355, YJL8359, and YJL8363/8364 were derived from YJL8259, YJL8271, and YJL8274, respectively, in two steps. An 1162 bp HindIII restriction fragment of pJL124

containing the *URA3* gene was used to convert *ura3-ΔORF* to *URA3*. Then a 3350 bp NgoMIV-Asp781I restriction fragment of pKJF019 was used to replace *URA3* with *tACT1-pGAL1/10-ΔntCDC6,cdk2A-tCDC6*. YJL8259 was derived from YJL7906 by loop-in/loop-out gene replacement of *YDRCTy2-1* with version 2 of *RA3* using PacI-linearized pKJF021. YJL8271 and YJL8274 were derived from YJL7906 and YJL8259, respectively, by loop-in/loop-out gene replacement of *YDRCTy1-1* with version 2 of *UR* using EagI-linearized pKJF022.

The non-re-replicating control strain (YJL9149/9150/9151) used in the uracil prototrophy assay (Figure S2) was derived from YJL8274 in 2 steps. An 1162 bp HindIII restriction fragment of pJL124 containing the *URA3* gene was used to convert *ura3-ΔORF* to *URA3.* Then a 2113 bp NgoMIV-Asp781I restriction fragment of pKJF020 was used to replace *URA3* with *tACT1-pGAL1/10 -tCDC6.*

Strains used to determine which recombination factors are involved in RRIGA using the uracil prototrophy assay (Figure 3) are all derived from YJL8363. YJL8407/8408, YJL8409/8410, YJL8412/8413, YJL8415/8416, YJL8418/8419, and YJL8421/8422 were generated by replacing *DNL4*, *RAD52*, *RAD51*, *RAD1*, *MSH3*, or *POL32*, respectively, with *TRP1*. Disruption fragments were generated using PCR in two steps. Step 1 primers (see Table S7) were used to amplify *TRP1* from pRS304 and add short regions of homology flanking the target gene. Step 2 primers extended the region of homology, using the PCR product obtained in Step 1 as a template.

Strains used to determine which recombination factors are involved in RRIGA using the sectoring assay (Figure S4) are all derived from YJL6558 (*MATa MCM7-2NLS ura3-52::{pGAL-ΔntCDC6-cdk2A, URA3} ORC2 orc6(S116A) leu2 trp1-289 ade2 ade3 bar1::LEU2* $ChrIV_{567kb}$*::{kanMX, ade3-2p, ARS317} ars317::natMX*) [3]. YJL7445 and YJL7451 were

generated by replacing *RAD1* or *RAD51*, respectively, with *TRP1*. Disruption fragments were generated using PCR in two steps. Step 1 primers (see Table S7) were used to amplify *TRP1* from pRS304 and add short regions of homology flanking the target gene. Step 2 primers extended the region of homology, using the PCR product obtained in Step 1 as a template.

Re-replicating strains (YJL8425/8426) for mapping DSBs by PFGE and Southern blotting (Figure 4, Figure S5) and the non-re-replicating controls (YJL8427/8428) were generated by replacing the *kanMX* drug resistance marker with an *hphMX* drug resistance marker along with an I-SceI recognition sequence in YJL6558 and YJL6974, respectively. The replacement fragment was generated using PCR to amplify the *hphMX* marker from pAG26 (see Table S7 for primers). A long tailed primer was used to add the I-SceI site and homology to the *ade3-2p* element in the reporter cassette upstream of *pTEF* in the *hphMX* marker.

All of the strains in which the location of *URA3* gene fragments at the amplicon endpoints were varied (Figure 5, Figure 6, Figure S7) were derived from YJL8842. YJL8842 was in turn derived from YJL8807 by deleting sequence from *tQ(UUG)D2* to *YDRCdelta9* through a loop-in/loop-out replacement using EagI-linearized pKJF027. YJL8807 was in turn derived from YJL8100 by deleting sequence from *YDRCdelta2* to *tQ(UUG)D1* through a loop-in/loop-out replacement using PacI-linearized pKJF026.

YJL9115/9116, YJL9118/9119, YJL9121/9122, YJL9127/9128, YJL9130/9131, YJL9133/9134 were all derived from YJL9054 by integrating a PCR product containing the *UR* element and a *TRP1* gene at $ChrIV_{607kb}$, $ChrIV_{650kb}$, $ChrIV_{753kb}$, $ChrIV_{875kb}$, $ChrIV_{985kb}$, or $ChrIV_{1100kb}$, respectively. YJL9136/9137 was derived from YJL9056 by integrating a PCR product containing the *UR* element and a *TRP1* gene at $ChrIV_{592kb}$. YJL9139/9140 was derived from YJL9057 by integrating a PCR product containing the *UR* element and a *TRP1* gene at

ChrIV$_{576kb}$. YJL9142/9143 was derived from YJL9060 by integrating a PCR product containing the *UR* element and a *TRP1* gene at ChrIV$_{713kb}$. YJL9145/9146 was derived from YJL9062 by integrating a PCR product containing the *UR* element and a *TRP1* gene at ChrIV$_{753kb}$. YJL9147/9148 was derived from YJL9067 by integrating a PCR product containing the *UR* element and a *TRP1* gene at ChrIV$_{753kb}$. Integration constructs were generated using PCR in two steps. Step 1 primers (see Table S7) were used to amplify the *UR* element and *TRP1* from pKJF028 and add short regions of homology flanking the target site. Step 2 primers extended the region of homology, using the PCR product obtained in Step 1 as a template.

YJL9054, YJL9056, YJL9057, YJL9060, YJL9062, and YJL9067 were derived from YJL8842 by integrating a PCR product containing the *RA3* element and an *hphMX* drug resistance marker at ChrIV$_{515kb}$, ChrIV$_{545kb}$, ChrIV$_{565kb}$, ChrIV$_{576kb}$, ChrIV$_{607kb}$, or ChrIV$_{650kb}$, respectively. Integration constructs were generated using PCR in two steps. Step 1 primers (see Table S7) were used to amplify the *RA3* element and *hphMX* from pKJF029 and add short regions of homology flanking the target site. Step 2 primers extended the region of homology, using the PCR product obtained in Step 1 as a template.

Strain Growth and Induction of Re-Replication

For synthetic medium, 1x amino acid concentrations were as described [6], except the amount of leucine was doubled to 60 μg/mL and the amount of serine was halved to 200 μg/mL. "C" indicates complete medium (all amino acids added). With the exception of plates for red/pink colony color development for the sectoring screen assay, all synthetic medium contained 2x amino acids. Color development plates contained 1x amino acids except 0.5x adenine (10 μg/mL). Synthetic medium contained 2% wt/vol dextrose. For SDC+5-FOA plates

5-flouroortic acid was added to a final concentration of 1 mg/mL. All cell growth was performed at 30°C.

To obtain reproducible induction of re-replication, cells were diluted from a fresh unsaturated culture grown in YEPD (YEP + 2% Dextrose) into YEPRaf (YEP + 3% wt/vol raffinose + 0.05% wt/vol dextrose) and allowed to grow exponentially for 12–15 hr overnight until they reached an OD_{600} of 0.2 -0.8. At this cell density, 15 μg/mL nocodazole (Sigma M1404 or US Biological N3000) was added for 120-150 min to arrest cells in G2/M phase. The *GAL1* promoter (*pGAL1*) was then induced by addition of 2-3% galactose for 3 hr (or 6 hours for break mapping experiments). Tight maintenance of the arrest was confirmed by analyzing the distribution of total DNA content by flow cytometry as previously described. For the cultures used for mapping DSBs, alpha-factor was also added to a final concentration of 50 ng/mL in order to prevent cells that escape arrest from entering the next S-phase. Following induction cells were plated onto SDC or SDC-Ura for the uracil prototrophy selection assay, or onto color development plates (described above) for the sectoring screen assay.

Colony Sectoring Assay

To score the frequency of red sectors, ~200 colonies were plated per color development plate (see Strain Growth above). Plates were incubated in the dark at 30°C for 5 days, then in the dark at room temperature for 2-6 days until colony color development was optimal. Plates were randomized and scored blind. Red sectors were counted if: 1) the sectors were greater than 1/8 of the colony, 2) darker red than the neighboring colonies (ie, not a pink sector in a nearly white colony) and 3) the junctions between the red sector and pink colony were largely straight, to minimize sectors due to poor growth. The frequency of sectored colonies was determined by

dividing the total sector counts by the total number of viable colonies. This frequency was measured in at least two independent experiments and the mean and standard error of the mean (when 3 or more trials were conducted), or the mean and the standard deviation (when only 2 trials were conducted) are reported (see Table S1).

Uracil Prototrophy Assay

To score the frequency of uracil prototrophs, following induction of re-replication ~5,000 cfu were plated onto each SDC-Ura plate (to isolate uracil prototrophs) and ~250 cfu onto each SDC plate (to determine an accurate cfu plated onto the SDC-Ura plates). Plates were incubated at 30°C for 3-5 days, then colonies were counted. The frequency of uracil prototrophs was determined by dividing the total number of colonies on the SDC-Ura plates by the number of cfus plated on the SDC-Ura plates. This frequency was measured in at least two independent experiments and the mean and standard error of the mean (when 3 or more trials were conducted), or the mean and the standard deviation (when only 2 trials were conducted) are reported (see Table S3). We note that this assay would score colonies that would not satisfy the criteria of red sector morphology described above, and consequently this assay consistently gave higher frequencies than the sectoring assay.

Pulsed Field Gel Electrophoresis and Southern Blotting

To make plugs for PFGE, 6×10^8 cells were transferred to a 50 mL conical tube and NaN_3 was added to a final concentration of 0.1%. Cells were then pelleted, washed twice with ice-cold 50 mM EDTA, then resuspended to 500 μL with 50°C SCE (1 M sorbitol, 0.1 M Na citrate, and 10 mM EDTA). Lyticase (L5263; Sigma) was added to a final concentration of 150

U/mL, and 475 µL of the sample was mixed with 475 µl of molten, 50°C 1% InCert agarose (Lonza, Rockland, ME), and then aliquoted into disposal plug molds (170-3713; Bio-Rad). The plug molds were allowed to solidify at 4°C, and then placed in SCEM + lyticase (1 M sorbitol, 0.1 M Na citrate, 10 mM EDTA, 5% β-mercaptoethanol (vol/vol), and 160 U/mL lyticase) for ~40 hr at 37°C. Plugs were then washed three times in $T_{10}E_1$ (10 mM Tris, pH 8.0, and 1 mM EDTA) for 1-2 hr each wash and resuspended in proteinase K solution (1% sarcosyl (wt/vol), 0.5 M EDTA, and 2 mg/ml proteinase K (Roche)) for >48 h at 55°C. Finally, plugs were washed five times in $T_{10}E_1$ for 1-3 hr each wash. The third $T_{10}E_1$ wash contained 1 mM PMSF to inactivate residual proteinase K. Plugs were then stored at 4°C in 0.5 M EDTA until used.

1/3 of each plug was cut off and washed twice with $T_{10}E_1$ for 1-3 hr on ice each wash. Plugs were then soaked in fresh $T_{10}E_1$ at 37°C for 16.5 hr (removes background fluorescence during ethidium bromide visualization of the gel). Plugs were then cooled to 4°C. Plugs were then soaked in 1x I-SceI digest buffer (Roche) on ice for 45 min. Then plugs were placed in 50 uL of 1x I-SceI digest buffer + 1 uL of I-SceI Enhancer (20 µg/mL, Roche) + 4 uL of I-SceI (10 U/µL, Roche) on ice for 1 hr. Digestion was started by adding $MgCl_2$ fo a final concentration of 5 mM and placing the plugs at 37°C. Digest proceeded for 1 hr, then plugs were placed in $T_{10}E_1$.

Plugs were loaded on a 1% SeaKem LE agarose (wt/vol) gel in 0.5x TBE (45 mM Tris, 45 mM borate, and 1 mM EDTA). A Lambda concatamer ladder (170-3635, Bio-Rad) was included for sizing purposes. The gel was electrophoresed in 14°C 0.5x TBE on a CHEF DR-III system (Bio-Rad) with initial switch time of 50 s, final switch time of 95 s, run time of 26 h, voltage of 6 V/cm, and angle of 120°. The gel was stained with 1 µg/mL ethidium bromide in 0.5x TBE for 30 min, then exposed to 130 mJ/cm^2 UV light using a Stratalinker 2400 (Stratagene, La Jolla, CA) to nick chromosomal DNA. The gel was then destained in deionized

water for 30 min, and quickly imaged with an AlphaImager. The gel was then soaked in two changes of denaturation buffer (0.5 N NaOH, 1.5 M NaCl) at RT for 15 min each. After rinsing with deionized water the gel was soaked in two changes of neutralization buffer (1.0 M Tris-Cl, 1.5 M NaCl, pH 7.5) at RT for 15 min each. The DNA was then transferred to a Roche positively charged nylon membrane using neutral downward capillary transfer (20X SSC, pH 7.0 used as transfer buffer). DNA was cross-linked to the membrane with 120 mJ/cm^2 of UV light in a Stratalinker 2400. The membrane was probed with a *MAK21* probe generated by PCR from yeast genomic DNA with oligonucleotides OJL2449 and OJL2450, and a *YOS9* probe generated by PCR from yeast genomic DNA with oligonucleotides OJL2231 and OJL2232. A Lambda probe was used to detect the sizing ladder. Images were collected using a Typhoon 9400 (GE Healthcare).

Break Mapping and Quantification

In our system a portion of Chromosome IV molecules at the G2/M boundary were induced to re-replicate *ARS317*, and a portion of these re-replicated chromosomes experienced breaks at various distances from *ARS317*. We wished to estimate the fraction of G2/M Chromosome IV that ended up acquiring a break within each approximately 2.5 kb distance interval from *ARS317.* To do this our analysis of the signal intensity from the southern blot had to correct or normalize for lane to lane differences in sample loading and run distance, for background lane signal from the T=0 hr (pre-induction MC_{2A}) sample, and for nonlinearity of fragment migration versus fragment size. Our starting point was a quantification of intensities obtained with ImageJ software (NIH) using a line trace about half the width of each lane. ImageJ divides each line trace vertically into a stack of thin lane slices each with a height

of 0.2 mm along the length of the lane. The software then assigns an intensity value for that slice (an average of the intensity across the width of the slice). This list of intensities versus lane slices was exported to Excel (Microsoft Corp.) for analysis.

To correct for slight lane to lane variability in electrophoretic migration, we offset all the lists so that a minor re-replication independent band (labeled in Figure S5 with an asterisk) aligned across all the lanes. This only required shifting lanes up or down by at most four slices relative to the adjacent lane(s).

To normalize for sample loading variability we assumed that the total signal in each lane (which comes from hybridization to a probe within 5 kb of *ARS317*) should be proportional to the amount of DNA in the *ARS317* re-replication peak as determined by aCGH. The aCGH peaks at the 3 hr and 6 hr time-points were 1.40625 and 1.84375 times the 2C DNA content at 0 hr (pre-induction) (data not shown). Hence the normalization scalar used for the list of 3 hr signal intensities was [(Total 0 hr Lane Signal)/(Total 3 hr Lane Signal)]*1.40625 and that used for the list of 6 hr signal intensities was [(Total 0 hr Lane Signal)/(Total 6 hr Lane Signal)]*1.84375. The total signal in each lane included all signal intensity from the well at the top of the lane to the end of the smear of fragmented DNA near the bottom of the lane so that all chromosome structures and fragments contributed to this normalization.

To correct for the background signal (T=0 hr lane) and thereby isolate the re-replication dependent signal at 3 hr and 6 hr, we subtracted the intensity for each lane slice at T=0 hr from the equivalently positioned slice at T=3 hr and T=6 hr. These re-replication induced signal intensities were then divided by the total signal in the T=0hr lane (including molecules trapped in the well) to obtain for each slice an amount of re-replication induced fragmentation as a percent of starting G2 chromosomes.

A Lambda DNA concatamer ladder run in parallel was used to assign the migration positions of fragments that increase in size steps of 48.5 kb. The physical distance separating each concatamer interval only underwent small changes between 97 kb to 485 kb for the southern analysis of breaks to the left of *ARS317* and between 145.5 kb to 485 kb for the southern analysis of breaks to the right of *ARS317.* Thus, within each concatamer interval in these size ranges, we presumed that the relationship between fragment size and migration distance was almost linear. The number of ImageJ lane slices assigned to each concatamer interval was between 16 and 22 in these size ranges. We separated the physical distance between each concatamer interval (representing a 48.5 kb range of fragment sizes) into 20 equal subintervals (representing a 2.425 kb range of fragment sizes), lined them up against the ImageJ lane slices, and identified the lane slice that overlapped the center of each subinterval. The fragmentation percent of that lane slice was then assigned to the subinterval after normalizing for the difference in physical height. For example, if the subintervals were only 0.18 mm in height (as opposed to the 0.2 mm height of the lane slices) the fragmentation percent was corrected by a factor of 0.18/0.20.

The relationship between fragment size and distance migrated became substantially non-linear for smaller fragments (between 48.5 kb and 97 kb for breaks to the left of *ARS317* and between 48.5 and 145.5 kb for breaks to the right of *ARS317*). Hence a polynomial fit for migration distance versus fragment size was calculated using the three smallest lambda concatamers (48.5kb, 97 kb, and 145.5 kb) and used to determine the center and height of each 2.425 kb subinterval in these regions. With the center and height, a fragmentation percent could be assigned to each subinterval as described above.

Finally, the fragmentation percent for each subinterval was plotted (using the midpoint of each subinterval) as a function of distance in kb from *ARS317*. This required one last adjustment because the fragment sizes from the Southern analysis map the distance of breaks relative to the I-SceI cut site. Those distances were adjusted by 5579 bp to account for the distance between *ARS317* and the I-SceI cut site.

Because our assumptions of linearity between fragment size and migration distance within each concatamer interval only approximated the true relationship between these two parameters, there are discernable discontinuities in the plot, especially at positions corresponding to the concatamer fragment sizes. Nonetheless the plot provides a good overall representation of the decline in break frequency as a function of distance from *ARS317*.

Our calculations also implicitly assume that every truncated linear chromosomal fragment comes from a separate chromosome; that is, no individual chromosome molecule gives rise to more than one fragment. If both the leading and lagging strands of a given fork were to break, however, this assumption would attribute these fragments to two separate re-replicating chromosomes when only one was actually involved. Nonetheless, in the unlikely case where every fork breaks on both strands, we would overestimate the fraction of chromosomes acquiring a break by at most two-fold. Hence, even if we corrected for such an extreme scenario, our data would still indicate a significant number of DSBs arise as a function of re-replication at positions compatible with our SSA model.

Genomic DNA Preparation for aCGH Analysis

Method 1: Isolates from the sectoring assay were cultured in YEP + 8% Dextrose until they reached saturation (so that most cells were in stationary phase with a 1C DNA content). Isolates from the uracil prototrophy assay were cultured in SDC-Ura media until they reached saturation

(so that most cells were in stationary phase with a 1C DNA content), or alpha factor was added to a final concentration of 100 ng/uL (to arrest cells with a 1C DNA content). For all isolates, ~25 OD units of cells were harvested and suspended in 1 mL of sterile water in microfuge tubes. Cells were then vortexed to wash and pelleted. The water was then aspirated off and the cell pellets were snap-frozen in liquid nitrogen. Cell pellets were thawed and resuspended in 200 uL of lysis buffer (2% Triton X-100, 1% SDS, 100 mM NaCl, 10 mM Tris-Cl, 1 mM EDTA (pH 8.0)). To this suspension 200 uL of phenol:chloroform:isoamyl alcohol (25:24:1) and 200 uL of acid washed glass beads (0.5 mm) were added (BioSpec Products, Inc., Bartlesville, OK). Cells were then lysed at room temperature using a Vortex-Genie 2 (Scientific Industries, NY) at top speed for 10 minutes. To the lysate 450 uL of 10 mM Tris-Cl, 1 mM EDTA (pH 7.5) was added, and the mixture was vortexed for 30 seconds. The mixture was then centrifuged at room temperature for 3 minutes at 20,800 x g. 500 uL of the aqueous phase was transferred to a new microfuge tube, to which 1 uL of 100 mg/mL RNase A (Qiagen, CA) was added. Samples were incubated at 37°C for 1-5 hours to allow digestion of the RNA. 300 uL of phenol:$CHCl_3$:isoamyl alcohol (25:24:1) was added to each sample, which were then mixed at room temperature for 5 minutes on a multi-mixer (Tomy Tech USA, CA), then centrifuged at room temperature for 3 minutes at 20,800 x g. 400 uL of the aqueous phase was transferred to a new microfuge tube. 300 uL of $CHCl_3$:isoamyl alcohol (24:1) was added to each sample. Samples were mixed by shaking at room temperature and were then centrifuged at room temperature for 3 minutes at 20,800 x g. 300 uL of the aqueous phase was transferred to a new microfuge tube, to which 750 uL of 100% Ethanol and 3 uL of 10N NH_4OAC (pH 7). Samples were vortexed then centrifuged at room temperature for 7 minutes at 20,800 x g to pellet the

DNA. Pellets were washed with 70% Ethanol the air dried. The DNA was suspended in 50 uL of 2 mM Tris-Cl (pH 7.8).

Method 2: To measure re-replication peaks (see Figure S4A) NaN_3 was added to a final concentration of 0.1% to 250 mL cultures (arrested with nocodazole and induced to re-replicate, as described above). To prepare reference DNA for all arrays (copy number as well as re-replication analysis), NaN_3 was added to a final concentration of 0.1% to 250 mL or 450 cultures that were arrested with alpha factor (final concentration = 100 ng/mL) or nocodazole (final concentration = 15 μg/mL). NaN_3 treated cultures were added to 25 mL or 50 mL (for 250 mL or 450 mL cultures, respectively) of frozen, -80°C, 0.2 M EDTA, 0.1% NaN_3. Cells were pelleted, washed with 50 mL of 4°C TE (10 mM Tris-Cl, 1 mM EDTA pH 7.5) and stored frozen at -80°C. Pellets were resuspended in 4 mL lysis buffer (2% Triton X-100, 1% SDS, 100 mM NaCl, 10 mM Tris-Cl, 1 mM EDTA pH 8.0) and mixed with 4 mL of phenol:$CHCl_3$:isoamyl alcohol (25:24:1) and 8 mL of acid-washed 0.5 mm glass beads (BioSpec Products, Inc., Bartlesville, OK). The suspension was vortexed seven times for 2-3 min separated by 2-3 min intervals at RT to get at least 95% of the cells lysed. The lysate was diluted with 8 mL phenol:$CHCl_3$:isoamyl alcohol (25:24:1) and 8 mL TE, vortexed once more**,** and then centrifuged at 18,500 x g for 15 min at RT. After collecting the aqueous phase, the interphase was re-extracted with 8 mL TE, and the second aqueous phase from this re-extraction pooled with the first. The combined aqueous phases were extracted with an equal volume of $CHCl_3$. The bulk of the RNA in the extract was selectively precipitated by addition of 0.01 volume 5 M NaCl (to a final concentration of 50 mM) and 0.4 volumes isopropanol followed by centrifugation at 9,000 x g for 15 min at RT. The RNA pellet was discarded and an additional 0.4 volumes of

isopropanol was added to the supernatant to precipitate the DNA. Following centrifugation at 9,000 x g for 15 min at RT, the pellet was washed with 70% ethanol, dried, and resuspended with 3.5 mL of 10 mM Tris-Cl (pH 8), 1 mM EDTA. RNase A (Qiagen, Valencia, CA) was added to 340 µg/mL and the sample incubated at 37ºC for 30-60 min. Then Proteinase K was added to 555 µg/mL followed by another incubation at 55°C for 30-60 min. Finally, 0.5 mL of 10% (w/v) Cetyltrimethylammonium Bromide (CTAB, Sigma H6269), 0.9 M NaCl (prewarmed to 65°C) and 0.9 mL of 5 M NaCl was added. The sample was incubated for 20 min at 65°C before being extracted with 8 mL $CHCl_3$:isoamyl alcohol (24:1) and centrifuged at 6000 x g for 15-180 min at RT. The DNA in the aqueous phase was precipitated with 0.8 volumes isopropanol at RT, washed with 70% ethanol, dried, and resuspended in 6 mL of 25 mM Tris-Cl (pH 7), 1 mM EDTA. RNase A (Qiagen, Valencia, CA) was added to 33 µg/mL and the sample incubated at 37ºC for 15 min. Then the following were added to the sample in the order listed: 1) 1.5 mL of 5 M NaCl; 2) 0.5 mL of 1M MOPS (pH 7); 3) 0.5 mL of Triton X-100 (3% vol/vol); 4) 1.5 mL of isopropanol. The sample was then mixed by vortexing, then purified on a Qiagen Genomic-tip 100/G column as per the manufacturer's instructions (Qiagen, Valencia, CA). The eluted DNA was precipitated with 0.8 volumes isopropanol at 4°C, washed with 70% ethanol, dried, and resuspended in 275 µL of 2 mM Tris-Cl pH 7.8. Genomic DNA was then sheared by sonication with a Branson Sonifier 450 to an average fragment size of 500 bp.

aCGH Analysis of Gene Amplification

50-100% of each DNA sample (prepared using Method 1 above) was labeled with Cy3 or Cy5 and 1.5-2ug of purified reference DNA from YJL6974 or YJL7695 (prepared using Method 2 above) was labeled with Cy5 or Cy3 (whichever was not used for the test sample) essentially

as described. The labeled DNA was isolated using one of two previously described methods (low-throughput [7] or high-throughput [8]). All samples were hybridized and analyzed as described [7]. Detailed results for aCGH of isolates from the sectoring screen assay and the uracil prototrophy assay are listed in Table S2 and Table S4, respectively.

aCGH Analysis of Re-replication

2-2.5μg of each DNA sample (prepared using Method 2 above) was labeled with Cy5 and 1.5-2μg of purified reference DNA from YJL7695 (prepared using Method 2 above) was labeled with Cy3 essentially as described [7], and labeled DNA was isolated as previously described [7]. All samples were hybridized and analyzed as described [7].

Error Calculation for RRIGA in Mutant Strains

RRIGA frequency is dependent upon how much re-replication occurs in a given strain. Since deletion of certain genes impacts the amount of re-replication from *ARS317* in our MC_{2A} strain background, the RRIGA frequencies reported in Figure 3 are normalized against a given strains amount of re-replication.

The amount of re-replication for a given strain for a 3 hr induction was measured using aCGH for 3 independent cultures for each strain (4 cultures for wild-type). The average peak value over the 2C baseline +/- 1 standard deviation was determined.

The frequency of uracil prototrophs measured is also subject to error, so this error was combined with the error from re-replication measurements to generate a "Combined Error" value as follows:

$$Combined\ Error\ =\ (Relative\ Combined\ Error) \times (Normalized\ Mean\ Amp.Freq.)$$

where $$Relative\ Combined\ Error\ =\ \sqrt{(a/b)^2 + (c/d)^2}$$

where $$a\ = Standard\ Deviation\ of\ Re - replication\ Peak\ Value$$

$$b =\ Mean\ Re - replication\ Peak\ Value$$

$$c = Standard\ Deviation\ of\ Amplification\ Frequency$$

$$d =\ Mean\ Amplification\ Frequency$$

and where

$$Normalized\ Mean\ Amp.\ Freq. = (Mean\ Amp. Freq.)/(Mean\ Re - replication\ Peak\ Value)$$

The data shown in Figure 3B represents the Normalized Mean Amplification Frequency +/- Combined Error. Note that the amplification used in this figure is the "Induced Amplification Frequency", which is computed as the frequency post induction minus the frequency pre-induction.

Junction PCR

Primers used for junction PCR to determine amplicon orientation and preservation of parental junctions are listed in Table S7. PCR was performed using Phusion DNA polymerase (Finnzymes) according to the manufacturer's instructions. DNA used for junction PCR was prepared using a spheoplasting mini-prep as follows. ~5x10^8 cells were suspended in 200 uL of 1 M Sorbitol, 0.1 M Sodium Citrate (pH 7.0), 60 mM EDTA, 0.8% (vol/vol) β-Mercaptoethanol, 2 mg/mL Zymolyase 20-T (MP Biomedicals) and incubated at 37°C for 70 min. Then 200 uL of 100 mM Tris-Cl (pH 9.0), 50 mM EDTA, 2% SDS was added to each sample, inverted to mix, then incubated at 65°C for 5 min. Then 200 uL of 5 M KAc was added to each sample, inverted to mix, then centrifuged at 20,800 x g for 10 min. 350 uL of the supernatant was transferred to a

new microfuge tube with 800 uL of 100% Ethanol. Samples were inverted to mix, the DNA was pelleted at RT at 3,800 x g for 2 min. Pellets were washed with 70% Ethanol, air dried, the resuspended in 200 uL of 0.5x TE (pH 8.0). PCR was performed using 0.25 uL of this DNA as template (in a 25 uL reaction).

References

1. Natsoulis G, Thomas W, Roghmann MC, Winston F, Boeke JD (1989) Ty1 transposition in Saccharomyces cerevisiae is nonrandom. Genetics 123: 269–279.

2. Sikorski RS, Hieter P (1989) A system of shuttle vectors and yeast host strains designed for efficient manipulation of DNA in Saccharomyces cerevisiae. Genetics 122: 19–27.

3. Green BM, Finn KJ, Li JJ (2010) Loss of DNA replication control is a potent inducer of gene amplification. Science 329: 943–946. doi:10.1126/science.1190966.

4. Green BM, Li JJ (2005) Loss of rereplication control in Saccharomyces cerevisiae results in extensive DNA damage. Mol Biol Cell 16: 421–432. doi:10.1091/mbc.E04-09-0833.

5. Goldstein AL, McCusker JH (1999) Three new dominant drug resistance cassettes for gene disruption in Saccharomyces cerevisiae. Yeast 15: 1541–1553. doi:10.1002/(SICI)1097-0061(199910)15:14<1541::AID-YEA476>3.0.CO;2-K.

6. Sherman F (2002) Getting started with yeast. Meth Enzymol 350: 3–41.

7. Green BM, Morreale RJ, Ozaydin B, DeRisi JL, Li JJ (2006) Genome-wide Mapping of DNA Synthesis in Saccharomyces cerevisiae Reveals That Mechanisms Preventing Reinitiation of DNA Replication Are Not Redundant. Mol Biol Cell 17: 2401–2414. doi:10.1091/mbc.E05-11-1043.

8. Pleiss JA, Whitworth GB, Bergkessel M, Guthrie C (2007) Transcript specificity in yeast pre-mRNA splicing revealed by mutations in core spliceosomal components. PLoS Biol 5: e90. doi:10.1371/journal.pbio.0050090.
